# Culture and gene expression profiling of Schwann cells from human sural nerve

**DOI:** 10.1038/s42003-026-11023-z

**Published:** 2026-09-28

**Authors:** Patricia García-Fernández, Thorsten Bischler, Annemarie Sodmann, Julia Grüner, Maximilian Koch, Eva Meller, Tom Gräfenhan, Robert Blum, Nurcan Üçeyler, Claudia Sommer

**Affiliations:** 1 Department of Neurology, University Hospital Würzburg, Würzburg, Germany; 2 Core Unit Systems Medicine, University of Würzburg, Würzburg, Germany; 3 Department of Neurosurgery, University Hospital Würzburg, Würzburg, Germany

**Keywords:** Cellular neuroscience, Mechanisms of disease, Neurodegeneration

## Abstract

Polyneuropathies (PNP) are diseases of the peripheral nervous system (PNS) whose pathophysiology remains poorly understood. Schwann cells (SC) are the principle glial cells in the PNS, and their dysfunction can lead to demyelination and secondary axonal degeneration, causing or exacerbating PNP. The study of SC can thus shed light on the molecular and cellular mechanisms involved in PNP. Protocols have been developed to isolate and culture human and animal SC from large amounts of starting material. The isolation of human SC from patients, where only small biosamples are available, remains a challenge. In this study, we present a protocol to culture human SC from ~4-mm diagnostic sural nerve biopsies and a comprehensive transcriptome analysis to characterize their phenotype. Our protocol allows the reproducible establishment of cultures containing proliferative human Schwann cells that can be stored, expanded, and used as tools for diagnostic applications.

## Introduction

Polyneuropathies (PNP) are systemic disorders of the peripheral nervous system (PNS)^1^. Schwann cells (SC) are the principal glial cells in the PNS. They derive from neural crest cells and develop according to a well-defined differentiation program^2^. In adult peripheral nerves, SC display specific functions in close association with axons. In myelinated nerves, SC form layers of myelin around axons in order to promote axonal health and the proper transmission of action potentials^3^. Many neuropathies are caused or exacerbated by dysfunctional SC, leading to demyelination and secondary axonal degeneration^4–6^. The study of SC biology can help identify basic molecular and cellular mechanisms involved in PNP.

In the last decades, animal models have helped to better understand the physiological and pathophysiological functions of SC^7–10^. Due to their ability to dedifferentiate and divide in culture, SC culture models, mostly harvested from rodent nerves, have been widely used^11^. One of the primary challenges in culturing SC is the unwanted proliferation of other cell types, such as fibroblasts. Fibroblasts from isolated nerves proliferate at a high speed in vitro and may overgrow the SC. To overcome this problem, multiple protocols have been established. These include, among others^12–14^, the use of mitotic agents^15–17^, immunopanning^18^, complement- or antibody-mediated cytolysis^19,20^, magnetic-activated cell sorting^21^, fluorescence-activated cell sorting^22^, separation based on adhesion properties^23^ or the use of D-valine media^24^. Some of these options, such as the use of mitotic agents, can, however, affect the survival, proliferation and/or phenotype of SC, while others require high amounts of primary culture material to achieve sufficient cell yield through cell sorting or separation. The use of D-valine media has proven to be safe and effective in the isolation of rat or chicken SC^24–26^. This medium replaces L-valine, an essential amino acid necessary for cell survival and growth, with its enantiomer, D-valine. Fibroblasts do not express the D-amino acid oxidase (DAAO) required to use up D-valine and therefore survive in this particular medium. This results in their elimination and promotes a heterogeneous SC culture. Notably, the use of D-valine media to isolate human SC has only been mentioned in a “letter to the editor” from 1990^27^ and has not been further tested.

In the last years, modifications of SC culture protocols have been used to culture and propagate human SC, which were harvested from post-mortem samples^28^, amputations^29^, or large nerve biopsies^30–33^. Such large amounts of material are usually not available from diagnostic biopsy samples. Hence, the isolation of human SC from patients, where only small biosamples are available, remains challenging. In this study, we describe a protocol that incorporates key elements of previously published culture conditions while enabling the successful establishment and expansion of primary cell cultures derived from ~4 mm diagnostic human sural nerve biopsies. In addition, we present a comprehensive transcriptomic analysis to characterize the Schwann cell populations present in these cultures and identify their phenotypes.

## Results

### Development of a SC isolation protocol

Six patients with painful PNP who were seen for diagnostic work-up and who underwent a diagnostic sural nerve biopsy were included. Diagnosis was made based on medical history, neurological examination, laboratory, and nerve conduction studies (Table 1). The median age of the patients (2 F/4 M) was 68.5 years (range 57–87), and the median disease duration was 3.5 years (range 0.017–15 years).

**Table 1:** Diagnoses and clinical characteristics

	Sex	Age	DD^a^ (years)	BMI	Diagnosis	Etiology
**Patient 1**	F	78	15	28.3	Sensorimotor neuropathy with IgM gammopathy	Waldenstrom’s macroglobulinemia (indolent B cell lymphoma)
**Patient 2**	M	84	0.17	30.1	Vasculitic neuropathy	Small vessel vasculitis and IgM kappa gammopathy
**Patient 3**	M	57	0.08	24.5	Vasculitic neuropathy	Granulomatosis with polyangiitis (ANCA-positive)
**Patient 4**	M	73	3	24.8	Distal symmetric axonal neuropathy	Idiopathic
**Patient 5**	M	64	3	30.1	Progressive idiopathic motor and sensory axonal neuropathy	Inflammatory
**Patient 6**	F	61	4	24.8	Axonal sensorimotor neuropathy	Idiopathic

^a^*BMI* body mass index, *DD* disease duration, *Ig* immunoglobulin.

**Fig. 1:**
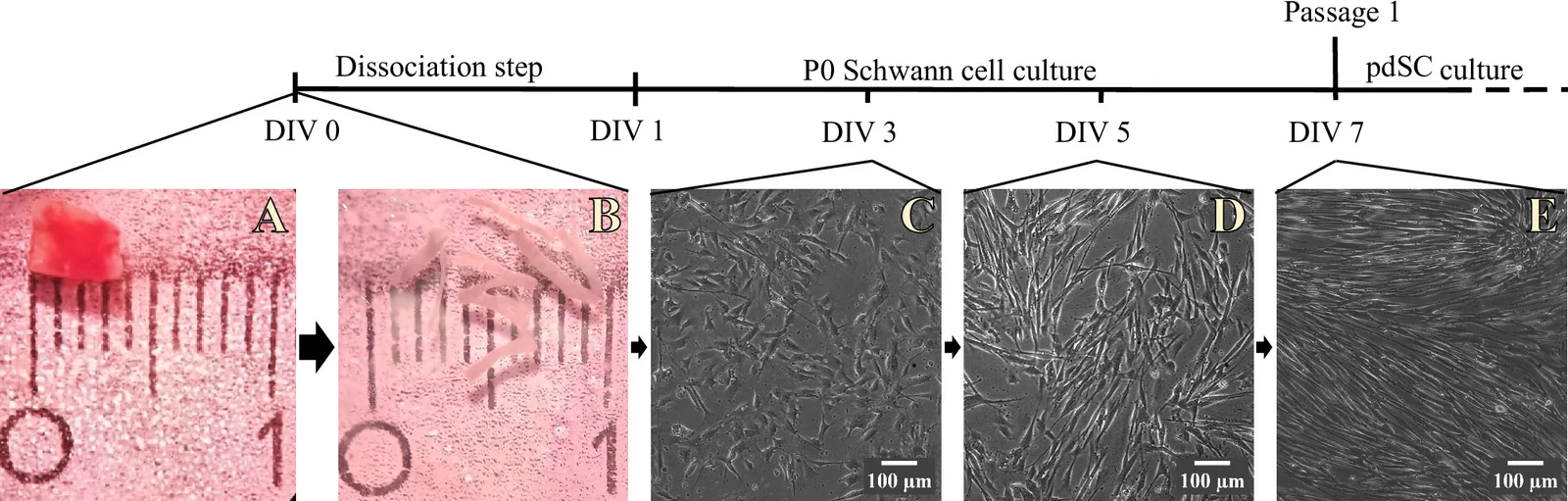
Isolation protocol of patient-derived SC. **A** Sural nerve biopsy specimen. **B** Extracted nerve fascicles. **C**–**E** Cultured SC. DIV days in vitro, P0/P1 passage 0/1, pdSC patient-derived Schwann cells.

From the sural nerve biopsies (one ~4 mm piece per patient), nerve fascicles were pulled out manually using sterile tweezers and incubated in dissociation medium (Fig. 1A, B, and “Methods”). After 20–24 h, fascicles were mechanically dissociated, and cells were seeded with supplemented D-Val medium. Cells grew in monolayers until confluence (Fig. 1C–E and “Methods”). When 80–90% confluence was reached, cells could be split.

### Characterization of SC

**Fig. 2:**
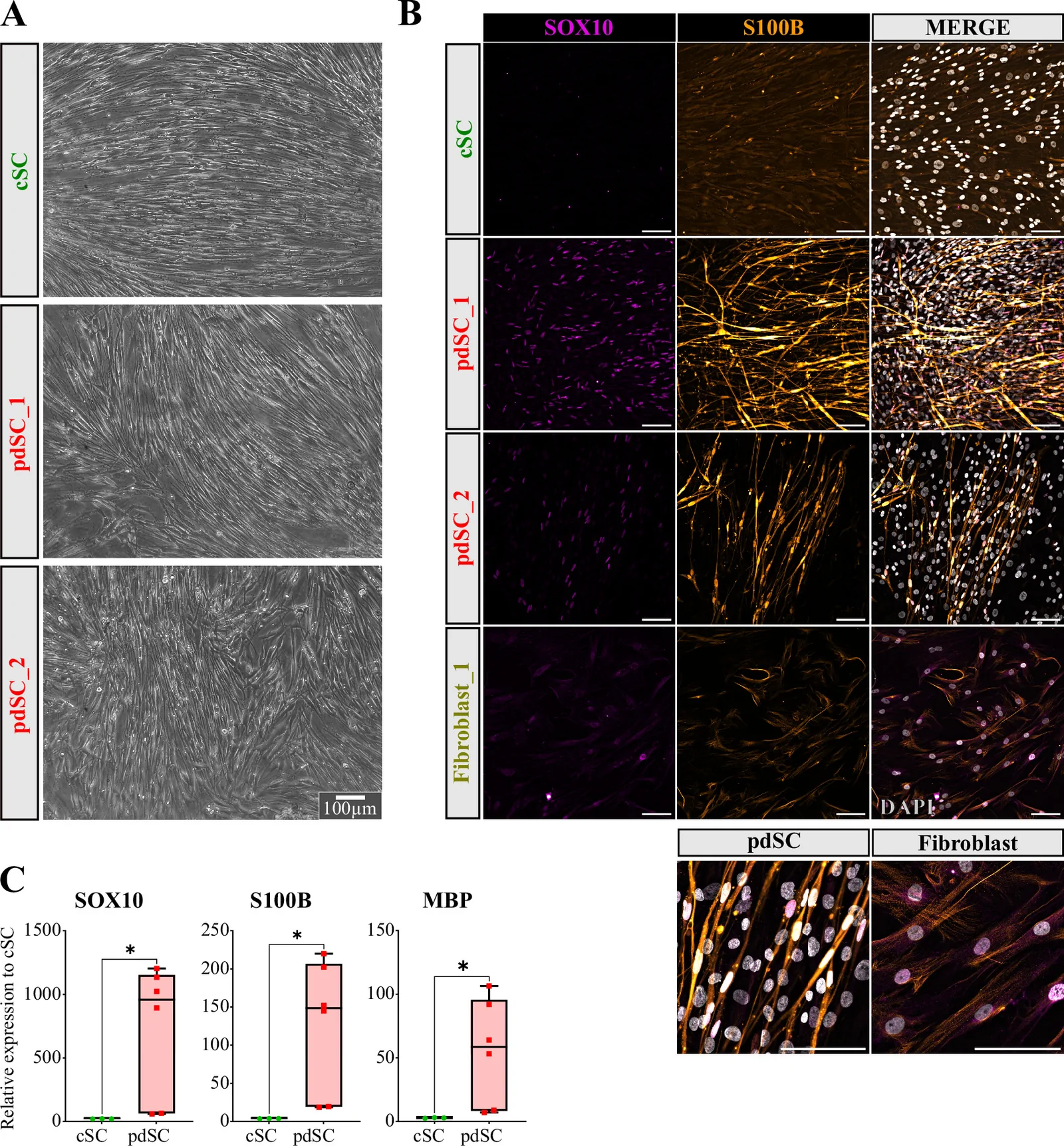
Characterization of patient-derived SC. **A** Phase-contrast images showing control human SC (*cSC*) and patient-derived SC (*pdSC*) cultures at DIV7. **B** Immunocytochemistry staining of SOX10 (magenta) and S100B (orange) in control human SC (*cSC*), patient-derived SC (*pdSC*) and fibroblast (*Fibroblast*_1) cell cultures at DIV7. Nuclei were stained with DAPI (grey). Scale bar: 100 µm. **C** Gene expression of *SOX10, S100B*, and *MBP* in pdSCs (*n* = 6), and cSCs (*n* = 3). Median ± range. *, *p* < 0.05.

After 5–7 days in vitro (DIV), patient-derived SC (pdSC) showed a spindle-like morphology, comparable to that of commercial control human SC (cSC) derived from human spinal nerve (Figs. 1E, 2A). Tests for optimization of the culture showed that pdSC developed this morphology upon reaching 60–80% confluence (Supplementary Fig. 1). In addition, seeding densities <50,000 cells per 9.6 cm^2^ dish in a 6-well plate and <10,000 cells per 3.5 cm^2^ in a 12-well plate led to reduced proliferation and low survival.

SC typically express specific markers like the SRY-box transcription factor 10 (SOX10), the S100 calcium binding protein B (S100B), and the myelin binding protein (MBP). To examine if these markers were expressed, we performed immunocytochemistry and RT-qPCR and compared the data to cSC. The immunofluorescence staining showed consistent expression of SOX10 and S100B in pdSC, with a lower expression in cSC and in a reference sample from a human fibroblast culture (Fibroblast_1) (Fig. 2B). This expression was heterogeneous among pdSC strains isolated from different patients (Supplementary Fig. 2A). In addition, the MBP marker showed low and unspecific results in all samples (Supplementary Fig. 2A). Moreover, we labeled for Fibronectin 1 (FN1), a marker commonly used to label fibroblasts. However, the marker is also present in SCs and can therefore be used to compare the morphology of both cell types (Supplementary Fig. 2B). While results showed expression of FN1 in all samples, differences in morphology between the cultured Schwann cells (pdSC) and fibroblasts (Fibroblast_1) could be clearly identified (Fig. 2B). RT-qPCR showed a higher expression of *SOX10*, *S100B* and *MBP* in pdSC compared to cSC (*p* < 0.05, Fig. 2C). On the protein level, cSC also showed low immunoreactivity for SOX10 and S100B (Supplementary Fig. 2A).

### Comparison between SC and fibroblast populations

To molecularly characterize pdSCs, we performed bulk RNA sequencing on patient-derived pdSCs and compared their transcriptomes with those of cSCs at three passages (DIV7). Nerve fascicles (NF) cultured for 48 h post-harvest were included as source tissue controls. Given potential fibroblast contamination and donor-specific variability in SOX10/S100B double-positive cells (Supplementary Fig. 2), a human fibroblast culture (Fibroblast_1) was analyzed as a reference. Sequencing yielded 2–4 × 10⁷ reads per sample (Supplementary Fig. 3A). Principal component analysis and sample distance heatmaps demonstrated strong within-group consistency (Supplementary Fig. 3B, C). Despite variability in fibroblast- and Schwann cell-associated signatures across individual cultures, all pdSC samples clustered closely together and were clearly separated from cSC, NF, and Fibroblast_1.

**Fig. 3:**
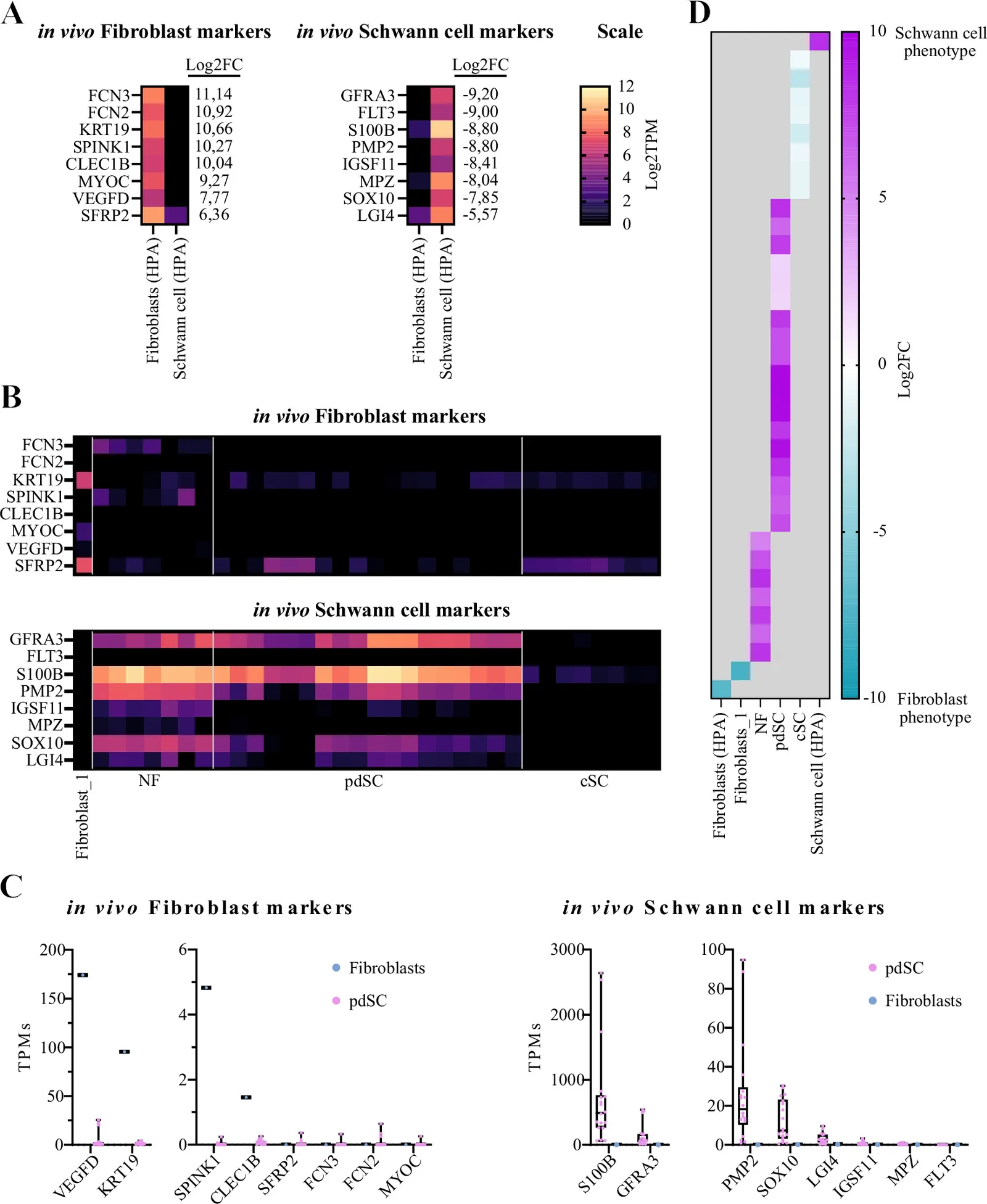
In vivo markers between SC and fibroblasts cell cultures. **A** Heatmap of the Log2TPM of in vivo Fibroblast markers (left) and in vivo Schwann cell markers (right) in fibroblast and Schwann cell populations from Human Protein Atlas (HPA), selected based on the Log2FC between them. **B** Heatmap of the Log2TPM of in vivo Fibroblast markers (up) and in vivo Schwann cell markers (down) in all samples included in a bulk-RNA sequencing analysis: Fibroblast_1 (*n* = 1), nerve fascicles (NF, *n* = 6), pdSC (*n* = 18), cSC (*n* = 8). **C** TPM of in vivo Fibroblast markers (left) and in vivo Schwann cell markers (right) in pdSC (*n* = 18), in comparison to Fibroblast_1 (*n* = 1). Mean ± SD. **D** Log2FC between the average TPM among all in vivo Schwann cell markers and the average TPM among all in vivo Fibroblast markers, per sample. cSC control human Schwann cells, FC fold change, pdSC patient-derived Schwann cells, TPM transcripts per million.

Due to the differences reported between in vivo and in vitro cell phenotypes, we studied specific markers found in both analyses. As there is no harmonized cell atlas from the peripheral human nerve available, we extracted single-cell transcriptomic consensus information from the Human Protein Atlas version 23.0 (HPA). This atlas provides single-cell categorization data from 31 different tissues and allows us to identify in vivo specific markers for many cell types^34–36^. From these data describing Fibroblasts (Fibroblast_HPA) and SCs (SC_HPA), we selected a set of eight genes with the highest (in vivo Fibroblast markers) and lowest (in vivo SC markers) log_2_-fold change (Log2FC) between both cell types (Fig. 3A).

Heatmap analysis based on the transcripts per million (TPM) data of each set (Supplementary Data 1) showed high expression of typical SC markers in pdSC, while low to no expression of these selected fibroblast markers (Fig. 3B). In particular, we showed that *SFRP2*, *KRT19*, and *MYOC* stood out as fibroblast-specific genes, while *S100B*, *GFRA3*, *PMP2*, and *SOX10* were abundantly expressed in pdSC (>10-fold, Fig. 3C). These results were supported by representing the Log2FC between average TPM of in vivo SC markers and in vivo Fibroblast markers, within each sample. Samples with a positive Log2FC (≥5 and up to 10) were related to a SC phenotype, while those with negative values (≤ − 5 and down to −10) were more likely to show a fibroblast phenotype (Fig. 3D and Supplementary Data 1). Our calculations indicated that SC_HPA (8.36), pdSC (7.05), and NF (6.78) presented a SC phenotype, while Fibroblast_1 (−7.65) and Fibroblast_HPA (−7.00) were more related to a fibroblast phenotype. Interestingly, cSC presented a neutral phenotype, neither characteristic for fibroblasts nor SC (−1.28), a finding in line with the PCA analysis (Supplementary Fig. 3B) and sample distance map (Supplementary Fig. 3C) showing that these cells exhibit an individual molecular profile.

**Fig. 4:**
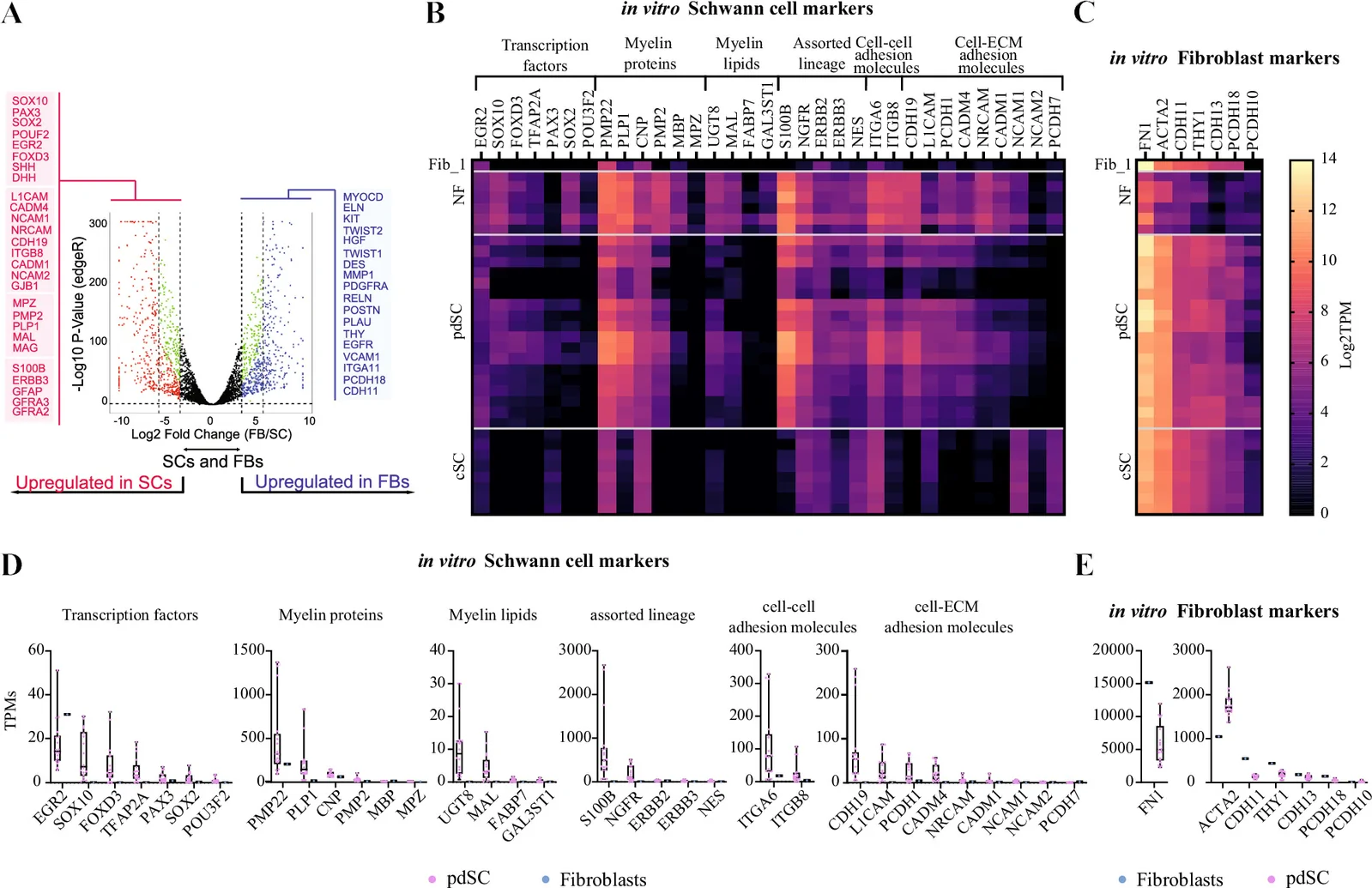
In vitro markers between Schwann cells and fibroblasts cell cultures. **A** Diagram modified from ref. ^37^ showing a volcano plot of the differential markers between Schwann cell and fibroblasts cultures, isolated from adult human peripheral nerve tissue. Genes expressed only in Schwann cells were coded in red (left), and the ones expressed only in fibroblasts were coded in blue (right). Heatmaps of Log2TPM values from our bulk RNA-seq data for in vitro Schwann cell markers, clustered by function (**B**), and in vitro Fibroblast markers (**C**): Fibroblast_1 (Fib_1, *n* = 1), nerve fascicles (NF, *n* = 6), pdSC (*n* = 18), cSC (*n* = 8). Bulk RNA-seq TPM values of in vitro Schwann cell markers, clustered by function (**D**), and in vitro Fibroblast markers (**E**) in pdSC (*N* = 18), in comparison to Fibroblast_1 (*n* = 1). Mean ± SD. cSC control human Schwann cells, pdSC patient-derived Schwann cells, NF nerve fascicles, TPM transcript per million.

To define a list of representative in vitro marker genes for each population, previously described differential expression of SC-specific or fibroblast-specific markers from primary cultures isolated from adult human peripheral nerve tissue was used (Fig. 4A)^37^. Heatmap analysis based on the TPM data of those markers in our data set confirmed abundant expression of in vitro SC markers in pdSC and in a minor extend in cSC (Fig. 4B and Supplementary Data 1). The expression of in vitro Fibroblast markers did not greatly differ between groups (Fig. 4C). Direct comparisons between pdSC and Fibroblast_1 highlighted some transcription factors (*SOX10, FOXD3, or TFAP2A*), myelin proteins (*PMP22, PLP1,* or *PMP2*), myelin lipids (*UGT8* or *MAL*), assorted lineage-related genes (*S100B, NGFR, ERBB2, ERBB3,* and *NES*), cell-cell adhesion molecules (ITGA6 and ITGB8), and cell-ECM adhesion molecules (*CDH19, L1CAM, PCDH1,* or *CADM4*) particularly enriched in pdSC (Fig. 4D). The expression of in vitro Fibroblast markers was present in both cell types, pointing out *FN1, CDH11,* or *THY1* as the only genes upregulated in fibroblasts in comparison to pdSC (Fig. 4E).

In ex vivo nerve fascicles, the source of the isolated pdSC (Figs. 3B, D, 4B), were low in some specific fibroblast markers, including *THY1* and *CDH13*. The data highlights *S100B, SOX10*, and *PMP2* as specific markers characteristic of both in vivo and in vitro SC and shows that cells isolated with this method present high gene expression levels (>100 TPM) of *S100B, PMP22, PLP1,* and *NGFR*, and moderate expression (>50 TPM) of *GFRA3, CNP, ITGA6,* and *CDH19*. In the control cell line (cSC), markers, such as *S100B, SOX10*, or *PMP2*, were barely detectable.

### Identification of patient-derived Schwann cell phenotype

In vitro, as shown above, patient-derived Schwann cells (pdSC) from the sural nerve regain their capacity for proliferation. This process is typically accompanied by phenotypic plasticity and a partial dedifferentiation from the mature Schwann cell state commonly observed in vivo. To identify the distinct phenotype of pdSC, markers from previous studies performing single-cell RNA sequencing of peripheral nerve specimens were analyzed.

**Fig. 5:**
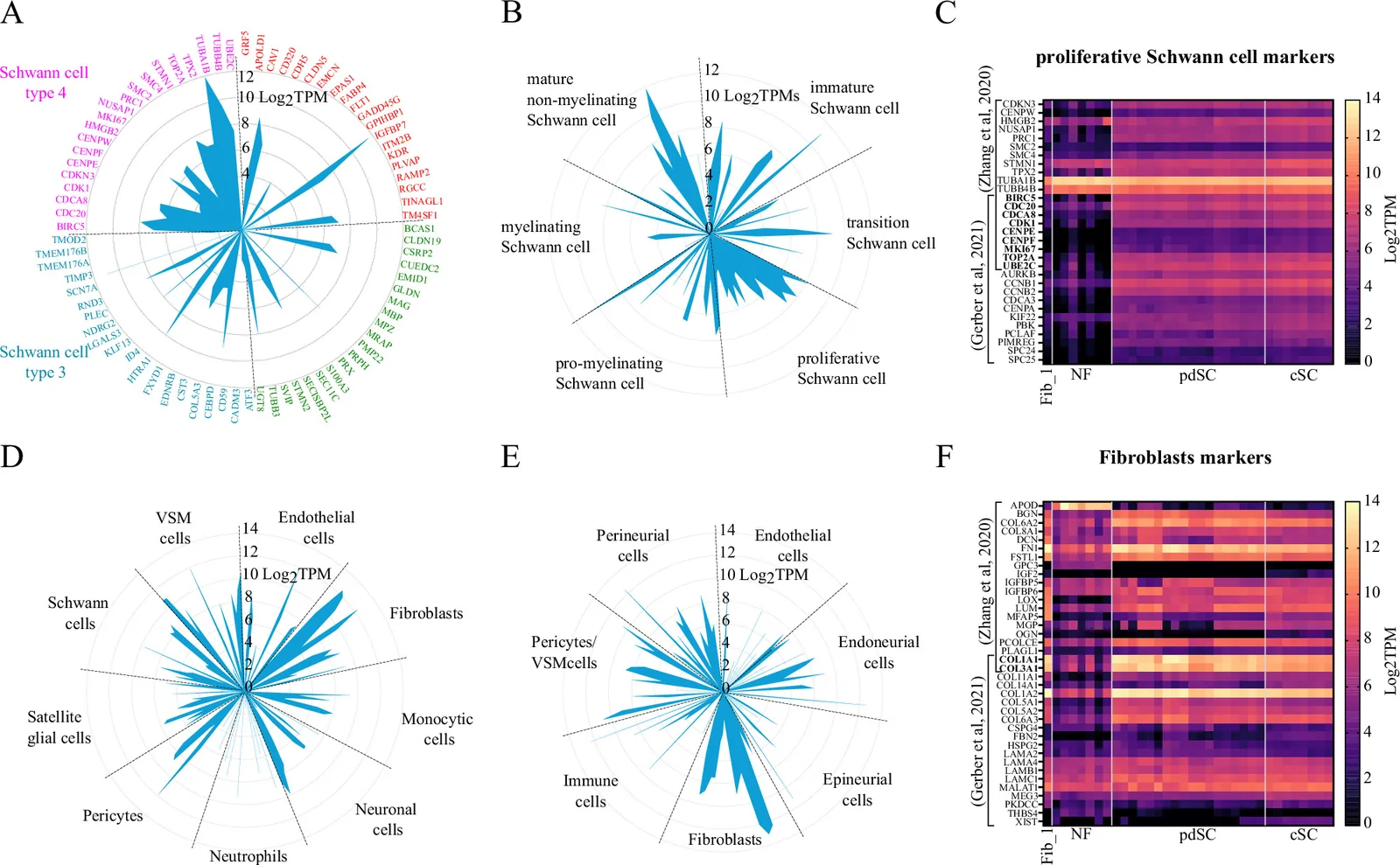
Molecular profiling identifies proliferative SC and fibroblast-like phenotypes in pdSC cultures. Radar charts of the expression in pdSC of markers characteristic of different Schwann cell types found in rat neonatal sciatic nerves and DRG^38^ (**A**) or in mouse sciatic nerves from P1 to P60^39^ (**B**), and of all cell types present in neonatal rat sciatic nerves and DRG^38^ (**D**), or mouse neonatal sciatic nerves^39^ (**E**). Top twenty differentially expressed genes per type were selected based on their ranked reported average log fold change values and represented as log2TPMs. Heatmaps of the Log2TPMs from our bulk RNA-seq data for proliferative Schwann cell markers (**C**) and Fibroblast markers (**F**), pooled from Zhang et al.^38^ and Gerber et al.^39^: Fibroblast_1 (*n* = 1), nerve fascicles (*n* = 6), pdSC (*n* = 18), cSC (*n* = 8). In **A**, Schwann cell type 1: markers characteristic of connective tissue and fibroblasts; Schwann cell type 2: markers related to myelin sheath formation; Schwann cell type 3: markers related to tissue development; Schwann cell type 4: markers involved in proliferation and cell cycle. cSC control human Schwann cells, NF nerve fascicles, pdSC patient-derived Schwann cells, TPM transcript per million, VSM cells vascular smooth muscle cells.

From two single-cell RNA-seq reference data sets from rodent newborn or young naïve sciatic nerves^38,39^, top twenty differentially expressed genes corresponding to different SC subtypes were selected, and their TPMs in pdSC were represented in radar charts (Fig. 5A, B, and Supplementary Data 1). Our results showed that pdSC presented a molecular profile related to a proliferative SC phenotype. This profile could also be observed in cSC, but was less pronounced in nerve fascicles or fibroblasts (Fig. 5C). In addition, we studied whether pdSC cultures presented a phenotype characteristic of other cell types residing in peripheral nerves, i.e.: endothelial cells, endoneurial cells, epineurial cells, fibroblasts, glial cells, immune cells, neuronal cells, vascular smooth muscle cells, pericytes or perineurial cells. For this, we extracted the top twenty differentially expressed marker genes for each cell type found in rodent newborn or young naïve sciatic nerves^38,39^. In pdSC, represented as TPMs in radar charts, we showed low to no expression of neutrophil, immune cell, and perineurial cell markers (Fig. 5D, E, and Supplementary Data 1). On the other hand, pdSC showed variable expression of specific genes from the remaining cell types. Among all, the expression of fibroblast markers was prominent in pdSC cultures. The elevated expression of fibroblast markers found in pdSC was also observed in cSC and Fibroblast_1, but not in nerve fascicles (Fig. 5F).

## Discussion

In this study, we present a novel protocol to culture human SC from ~4-mm diagnostic sural nerve biopsies. The resulting cultures were characterized and expanded to generate primary sural nerve-derived cultures from patients with PNP that may serve as useful tools for diagnostic and therapeutic applications. Application of this protocol resulted in the establishment of cultures containing spindle-shaped cells with nuclear localization of SOX10 and cytoplasmic expression of S100B and MBP, consistent with previously described SC hallmarks^40–44^. The analysis of their transcriptome confirmed the expression of *S100B* and *SOX10*, but not *MBP*, in cells cultured with this method.

S100B is a calcium-binding protein that is highly expressed in different glial cells, including SC, but that has also been reported in other cell types, such as chondrocytes, melanocytes, Langerhans cells, dendritic cells and more, but not in fibroblasts^45^. SOX10 is a transcription factor expressed exclusively in cells derived from the neural crest, such as SC. Expression of SOX10 is important for the early development of the SC lineage and continues to be required during multiple steps of SC development, so much so that deletion of SOX10 arrests SC development at the immature stage, impairs myelination and compromises SC identity and homeostasis^46,47^. Our analysis of genes characteristic for in vivo and in vitro SC finds S100B and SOX10 as specific markers common to all SC. The expression of SOX10 and S100B in cells cultured with this protocol thus confirms the presence of dividing cells of the SC lineage.

Our multiclonal primary cultures also contained varying proportions of SOX10- and S100B negative cells. Cells lacking these markers in SC cultures are frequently interpreted as contaminating fibroblasts^37,48^. Peripheral nerve biopsies contain several non-SC populations, including endoneurial fibroblasts, which may be co-isolated during tissue dissociation and subsequently expand under in vitro conditions. On the other hand, SC are also known to display considerable phenotypic plasticity in culture. In particular, proliferative culture conditions, including high serum concentrations or mitogenic stimulation, such as those used in this protocol, have been reported to promote proliferation and to downregulate canonical SC markers, including SOX10^11,49^. Moreover, experimental loss of SOX10 in SC has been associated with increased proliferation, enhanced focal adhesions, and a flattened morphology similar to that observed in some of our cultures^50^. Lastly, during early protocol optimizations, fibroblasts were clearly present in our cultures, and they rapidly overgrew under high-serum conditions, hindering SC isolation, which is no longer observed.

Given these considerations, we acknowledge the limitation that fibroblasts co-isolated from the original biopsy may be present in the cultures. In addition, fibroblast-like phenotypes may reflect Schwann cells or other co-isolated cell types adopting alternative states under the applied culture conditions.

The only other marker that was common to in vivo and in vitro SC was the peripheral myelin protein 2 (PMP2). PMP2 (or P2) is one of the three major myelin proteins, along with myelin protein zero (MPZ or P0) and myelin basic protein (MBP or P1) identified in the PNS^51^, and that are thus expressed mainly in myelinating SC, among other glial cells^34^. SOX10 is believed to regulate the expression of these three myelin proteins^52–54^, and its upregulation would thus explain the specific expression of PMP2 in pdSC, in comparison to a null expression in fibroblasts. MPZ and MBP, on the other hand, were low and equally expressed in all SC (pdSC and cSC) and in fibroblasts. However, while MPZ and MBP have been described to play roles in myelin compaction, PMP2 has been suggested to have a different function. For instance, it has been found upregulated upon treatment with forskolin and being expressed prior to MBP and MPZ after peripheral nerve crush^51^. The expression of PMP2 may thus not be exclusively related to myelination. These results, along with our analyses of the different Schwann cell phenotypes and the observed proliferation in culture, suggest that pdSC may be in a proliferative state rather than in a myelinating state, and therefore lack the capacity to myelinate axons. This interpretation is consistent with the well-established observation that Schwann cells rapidly dedifferentiate, downregulate myelin-related genes and enter a proliferative repair-like state when cultured or deprived of axonal contact^55–59^.

The transition from a proliferative to a myelinating stage is highly coordinated by the levels of intracellular cyclic adenosine monophosphate (cAMP), a key regulator of metabolic activity, survival, proliferation and differentiation. For instance, in the presence of forskolin, a potent inducer of cAMP, the growth factors neuregulin-1 (NRG1), platelet-derived growth factor (PDGF), and fibroblast growth factor (FGF) can induce a synergistic proliferative response in SC^60–62^, enhanced by the inclusion of serum. On the other hand, the high intracellular levels of cAMP are also necessary for the expression of myelination-associated genes, while the presence of serum, NRG1, PDGF or FGF can inhibit this expression. Notably, cells in a myelination state do not have the capacity to proliferate anymore, and it has been suggested that inhibition of the cell cycle may be necessary to trigger myelin-specific differentiation^60,63,64^. The presence of forskolin together with NRG1, PDGF, FGF and serum in our supplemented D-val medium thus explains the high proliferation capacity of pdSC, and their removal might be necessary to promote a SC shift towards a myelinating stage. Notably, fibroblasts are cells generally described with a high proliferative capacity^65^. Some of the genes highly expressed in both Fibroblast_1 and pdSC, such as fibronectin 1 (FN1), have been closely associated with cell proliferation^66,67^. Thus, the proliferative phenotype of pdSC may explain the close genetic profiling to a fibroblast cell type.

Interestingly, in most mammalian cells, such as fibroblasts^68^ or muscle smooth cells^69^, an activation of the cAMP-dependent protein kinase A pathway can induce growth arrest and cell differentiation. The culture method presented in this study uses D-valine media with the aim to selectively inhibit the proliferation of fibroblasts and obtain a SC culture. Moreover, forskolin in the culture medium may also contribute to the inhibition of fibroblast expansion in the culture. In fact, previous authors have questioned the efficacy of D-valine media in the isolation of human SC based on the low gene expression of the DAAO enzyme^29^, necessary to metabolize D-valine into its enantiomer L-valine and thus survive in this medium. Its capacity to inhibit proliferation of human fibroblast, nevertheless, has been described since 1975^70,71^ and has been used in human samples to isolate smooth muscle cells^72^, endometrial stromal cells^73^, epithelial cells^74,75^, and SC^27^. In addition, it has been extensively applied for the isolation of rat SC^24^, as well as of other cell strains from different species^76–81^. Our data show low expression of DAAO in all cell types (<1 TPM), as can be found in different available data atlases from human^29,34^ and rodent^82^ samples. These findings suggest that inhibition of contaminating cells, such as fibroblasts, may be driven less by L-valine deprivation or D-valine supplementation and more by the presence of forskolin. Nevertheless, our results demonstrate that D-valine-supplemented medium is a safe and effective approach for establishing and expanding human sural nerve-derived SCs, while limiting the expansion of contaminating cell populations.

We used commercially available Schwann cells (cSCs) as a reference in our study. Previous reports have shown that these cells display key hallmarks of Schwann cells^83–85^. In our hands, cSCs exhibited proliferation behavior and morphology comparable to those observed in our pdSCs. However, transcriptome analysis revealed that these cell preparations formed a distinct cluster, clearly separated from both fibroblasts and our patient-derived SCs. These findings suggest that morphological assessment, proliferation characteristics, and antibody staining alone may not be sufficient to reliably define cellular identity in vitro. Instead, bulk mRNA sequencing provides a robust molecular approach for the characterization and classification of cell preparations and how they behave in individual laboratories. Given the increasing accessibility of sequencing technologies and open science datasets, transcriptomic profiling represents a valuable tool for verifying cell identity and for comparing cell lines across laboratories with high transparency.

While our method allows the culture of proliferative human SC from diagnostic sural nerve biopsies, the study of different culture conditions would help identify protocols to switch pdSC into each of the desired SC phenotypes. In the future, integration with publicly available single-cell RNA-sequencing datasets from human sural nerve tissue may help to further resolve cellular heterogeneity and plasticity in Schwann cell cultures derived from patient biopsies. Furthermore, while all cultures were generated using identical culture conditions, variability between samples may reflect donor-dependent biological factors, as well as arise from technical limitations or intrinsic cell-to-cell heterogeneity. Our aim was limited to confirming Schwann cell identity using accessible, curated datasets; therefore, the comparisons presented here should be interpreted as representative, rather than comprehensive. Moreover, future work incorporating single-cell transcriptomic analyses will be essential to dissect the cellular composition in greater detail, including distinct SC subtypes and transitional states between clusters. Lastly, due to the invasive nature of nerve biopsy procedures, obtaining nerve samples from healthy volunteers was not ethically permissible. Under other circumstances, the isolation of Schwann cells from healthy donors could help clarify the contribution of neuropathy to the characteristics observed in each cell strain.

In summary, our protocol provides proof of principle for the expansion of proliferative human Schwann cells from ~4 mm diagnostic sural nerve biopsies, yielding sufficient cell numbers for downstream analyses. pdSCs thus represent a valuable tool for studying neuropathy pathophysiology and advancing personalized medicine.

## Methods

### Inclusion and ethics

In 2021, six patients with PNP were recruited at the Department of Neurology, University Hospital Würzburg, Germany, where they were seen for diagnostic work-up. Our study was approved by the Würzburg Medical Faculty Ethics Committee (# 15/19), and all participants gave written informed consent prior to inclusion. Diagnoses were based on history taking and neurological examination, laboratory studies, and nerve conduction examinations.

### Sample collection

From each patient, a sural nerve biopsy was performed at the Department of Neurosurgery, University Hospital Würzburg, Germany, as described previously^86^. Nerves were cut into ~4-mm pieces, from which one piece was used for the study. This 4-mm sural nerve piece was put in ice-cold DMEM/F12 medium (#11320-074, Gibco), and fascicles were pulled out with tweezers to remove as much connective tissue as possible.

In two wells of a 96-well plate, two fascicles of similar size were put in 200 µl of supplemented DMEM/F12 medium. This medium consisted of DMEM/F12 containing 100 U/ml penicillin-streptomycin (15140-122, Gibco), 10% (w/v) FBS, 20 mM 4-(2-hydroxyethyl)-1-piperazineethanesulfonic acid (HEPES) (#15630-056, Gibco), and 1× (w/v) N2-supplement (#17502048, Gibco). The medium was freshly supplemented with 50 ng/ml heregulinβ-1 (#100-03, PeproTech), 10 ng/ml FGF-basic (#100-18B, PeproTech), 10 ng/ml PDGF-AA (#100-13A, PeproTech), and 5 μM forskolin (#F6886, Sigma) and incubated at 37 °C and 5% CO_2_. After 48 h in culture, nerve fascicles were collected in 700 µl of Qiazol lysis reagent (Qiagen, Hilden, Germany) and stored at −80 °C for RNA isolation. Remaining fascicles were processed for isolation of pdSC.

### Patient-derived Schwann cell isolation and culture

#### pdSC isolation

Four to six nerve fascicles were divided into two wells of a 12-well plate and incubated with 2 ml of dissociation medium per well, in a standard incubator at 5% CO_2_ and 37 °C for 20–24 h. Medium consisted of supplemented DMEM/F-12 medium containing 0.125% (w/v) collagenase type IV (#17104-019, Gibco), 1.25 U/ml dispase II (#D4693-1G, Sigma), and 3 mM calcium chloride (CaCl_2_). The next day, fascicles were mechanically dissociated into a suspension using a 1000 μl micropipette. Next, the suspension from each well was diluted with 8 ml of washing medium (DMEM/F-12 media containing 10% (w/v) fetal bovine serum (FBS) (#F7524, Sigma)) to stop the activity of dissociation enzymes. Cells were then pelleted at 300 × *g* for 5 min at RT.

After aspirating the supernatant, each cell pellet was resuspended and then pooled in 0.5 ml supplemented D-val medium (MEM Eagle D-valine Modification w/L-Glutamine (#M3861-20, Biomol) containing 2.2 g/l sodium hydrogen carbonate (NaHCO3)(#HN01.2, CarlRoth), 100 U/ml penicillin-streptomycin (#15140-122, Gibco), 10% (w/v) FBS, 20 mM 4-(2-hydroxyethyl)-1-piperazineethanesulfonic acid (HEPES) (#15630-056, Gibco), 2 mM L-Glutamine (#25030-024, Gibco) and 1× (w/v) N2-supplement (#17502048, Gibco) and supplemented with 50 ng/ml heregulinβ-1 (#100-03, PeproTech), 10 ng/ml FGF-basic (#100-18B, PeproTech), 10 ng/ml PDGF-AA (#100-13A, PeproTech), and 5 μM forskolin (#F6886, Sigma)).

The resuspended cells were divided into 2–3 wells of a Matrigel hESC-Qualified Matrix, LDEV-free (#354277, Corning)-coated 12-well plate, preincubated with 0.5 ml of supplemented D-val medium (see above), ensuring a minimum of 10,000 cells per 3.5 cm^2^ dish. Cells at passage 0 (P0) were incubated in a standard incubator at 5% CO_2_ and 37 °C, and the whole medium was replaced every 2–3 days.

### pdSC expansion

After 7 DIV, or when 90% confluency was reached, cells were split as follows: medium was removed, and each well was washed twice with 37 °C Dulbecco’s phosphate-buffered saline (PBS). Then wells were incubated with 1 ml 37 °C TrypLE (#12604-021, Gibco) in a standard CO_2_ incubator for 15 min. The TrypLE was gently pipetted up and down with a 100 µl pipette to collect the remaining attached cells. The resulting cell suspension was diluted five times in 37 °C washing medium and centrifuged at 300 × *g* for 5 min at RT. After aspirating the supernatant, each cell pellet was resuspended in 1 ml supplemented D-val medium and pooled together. The resulted cell suspension from P0 was divided into three well of a 6-well plate and incubated for 7 days or until the culture reached 90% confluency. Then, following the same protocol, P1 cells were pooled and expanded into 3 × 25 T flasks until cells reached confluency, whereon they were ready for cryopreservation.

### pdSC cryopreservation

In order to freeze pdSC, the cells were detached and collected in a suspension as described above. The cells were then centrifuged at 300 × *g* for 5 min at RT, and the pellet was diluted in ice-cold freezing medium (90% FBS and 10% DMSO) at a concentration of 2 million cells/ml. One milliliter per freezing vial were cooled down 1 °C/min until −80 °C through a CoolCell™ Cell Freezing Vial Containers (#15542771, Corning). The next day, vials were stored in liquid nitrogen.

After a minimum of 1 week in N_2_, cell vials could be thawed in a water bath at 37 °C, mixed with 8 ml of ice-cold DMEM/F-12 and centrifuged at 300 × *g* for 5 min at RT. After aspirating the supernatant, each cell pellet could be resuspended in supplemented D-val medium and cells were cultured at recommended densities (see above).

### Control samples

As control of a Schwann cell type, commercial human Schwann cells (cSCs) (#1700, ScienceCell) isolated from human spinal nerve were purchased. Upon arrival, the frozen vial containing >5 × 10^5^ cells was thawed (numbered as P1) and cultured in supplemented D-Val medium in 3 wells of a Matrigel hESC-Qualified Matrix, LDEV-free (#354277, Corning)-coated 12-well plates. Cells were expanded following the same protocol as for pdSC and cryopreserved at P3.

As control of a fibroblast cell type, a sample isolated from a skin biopsy of a healthy volunteer and previously described^87–89^ was used. In summary, a 6-mm skin punch biopsy from the calf was cut into small dermal pieces and placed in fibroblast medium (DMEM/F-12 with 10% FCS and 1% penicillin/streptomycin) for 1 h to allow tissue adherence before adding additional medium. Cultures were maintained with medium changes every 2–3 days. Cells were expanded and further processed at passage four.

### Immunocytochemistry

After thawing, cSC, pdSC, and Fibroblast_1 were seeded in coverslips in a 24-well plate and fixed in paraformaldehyde 4% 10 min at DIV7 after reaching the desired confluency. Next, coverslips were washed with PBS and permeabilized with 0.1% Triton X100/PBS for 12 min at RT, and blocked with 5% FBS/0.3% Triton X100 /PBS for 30 min. Samples were then incubated with the primary antibodies monoclonal mouse to SOX10 (1:50, #SC-365692, Santa Cruz, RRID: AB_10844002), monoclonal rabbit to S100B (1:500, #ab52642, Abcam, RRID:AB_882426), monoclonal rat to MBP (1:100, #ab7349, Abcam, RRID:AB_305869) and/or monoclonal mouse to FN1 (1:100, #SC-8422, Santa Cruz, RRID: AB_627598) in 1% FBS/0.1% Triton/PBS over night at 4 °C. The next day, the primary antibody was washed with PBS and samples were incubated with the secondary antibodies donkey anti-mouse-Cy3 (#715-165-151, Dianova, RRID: AB_2315777), donkey anti-rabbit-AF647 (#715-165-152, Dianova, RRID: AB_2492288), and donkey anti-rat-AF488 (#715-165-150, Dianova, RRID: AB_2340683), in 1% FBS/0.1% Triton X100/PBS for 2 h at RT. After thoroughly washing with PBS, samples were incubated with 4′,6-Diamidino-2-phenylindole dihydrochloride (DAPI) (1:20000 in PBS, #32670-5MG-F, Sigma) for 10 min, washed and mounted with Aqua-Poly/Mount (#18606-20, Polysciences, PA, USA). Samples were visualized with a Leica DMi8 fluorescence microscope (Leica, Wetzlar, Germany), and pictures were processed in Fiji (Image J)^90^.

### Gene expression analysis

#### RNA extraction

For the gene expression analysis, three wells of a 12-well plate confluent with pdSCs at P3 from each patient and three wells of cSCs at P4, P5, and P6 were collected in Qiazol lysis reagent (Qiagen, Hilden, Germany) and stored at −80 °C.

The cSCs were commercial and derived from a single donor (*n* = 1 biological replicate). The pdSC cultures were derived from 6 independent donors (*n* = 6 biological replicates). Technical replicates and pdSCs at different passages were included in the final analyses as part of the total *n*-number.

Purification of total RNA, cell samples were homogenized in 700 µL of Qiazol lysis reagent with a homogenizer disperser. RNA was isolated using the RNeasy kit (Qiagen, Hilden, Germany), following the manufacturer’s protocol. Samples were stored at −80 °C after the RNA quality and quantity was assessed with a NanoDrop™ One (Thermo Fisher Scientific, Waltham, MA, USA).

### Reverse transcription

For cDNA synthesis from mRNA, TaqMan Reverse Transcription reagents (Thermo Fisher Scientific, Waltham, MA, USA) were used. Two hundred fifty nanograms mRNA of each sample was pre-incubated with 5 µl random hexamer 50 µM (N8080127) at 85 °C for 3 min. Next, 10 μl 10× PCR buffer (N8080130), 22 μl MgCl_2,_ 20 μL deoxyribonucleoside triphosphate 10 mM (4303441), 6.25 μL multiscribe reverse transcriptase 50 U/µl (4311235) and 2 μL RNase inhibitor 20 U/µl (N8080119) were added per sample. Lastly, reaction was performed under these conditions: annealing (25 °C, 10 min), reverse transcription (48 °C, 60 min), and enzyme inactivation (95 °C, 5 min). Reactions were carried out on a PRISM 7700 Cycler (Applied Biosystems, Waltham, MA, USA), and transcribed cDNA was stored at −20 °C until further analysis.

### RT-qPCR

RT-qPCR was carried out with TaqMan qRT-PCR reagents (all Thermo Fisher Scientific, Waltham, MA, USA) and pre-designed assays in a StepOnePlus Real-Time PCR System (Thermo Fisher Scientific, Waltham, MA, USA). RT-qPCR was performed for the targets *SOX10* (#Hs00366918_m1), *S100B* (#Hs00902901_m1) and *MBP* (#Hs00921945_m1). For target normalization, *RPL13A* (#Hs04194366_g1) was selected as suitable endogenous control, showing to be stable across groups. All primers were purchased from Thermo Fisher Scientific. For each reaction, 3.5 µL cDNA was mixed with 0.5 µL nuclease free water, 5 µL Fast Advanced Mastermix and 0.5 µL RPL13A primer and 0.5 µL target primer.

Each mRNA was amplified in triplicates and relative quantification (RQ) values were obtained by the StepOnePlus™ Software v2.3 (Thermo Fisher Scientific, Waltham, MA, USA) using interplate calibrators through the ∆∆Ct method.

### RNA sequencing

RNA quality was checked using 2100 Bioanalyzer with RNA 6000 Nano kit or 5200 Fragment Analyzer DNF-471-33-SS Total RNA 15nt kit (Agilent Technologies). The RIN/RQN for all samples was ≥8.6. cDNA libraries suitable for sequencing were prepared from 10 ng input with SMART-Seq® v4 Ultra® Low Input RNA Kit (Takara) according to manufacturer’s instructions (1/4 volume). The PCR amplification was performed using 10–12 PCR cycles. Libraries were quantified by QubitTM dsDNA HS Assay Kit (3.0 Fluometer; ThermoFisher), and quality was checked using 2100 Bioanalyzer with High Sensitivity DNA kit (Agilent Technologies). Zero point five nanograms of each library was subjected to a tagmentation-based protocol (Nextera XT, Illumina) using a quarter of the recommended reagent volumes. Libraries were quantified again by QubitTM dsDNA HS Assay Kit (3.0 Fluometer; ThermoFisher), and quality was checked using 2100 Bioanalyzer with High Sensitivity DNA kit (Agilent Technologies) before pooling. Sequencing of pooled libraries, spiked with 1% PhiX control library, was performed in single-end mode with 100 nt read length on the NextSeq 2000 platform (Illumina) with two P2 sequencing kits and 16–39 Mio reads per sample. Demultiplexed FASTQ files were generated with bcl-convert v4.0.3 or v4.2.4 (Illumina).

To assure high sequence quality, reads were quality- and adapter-trimmed via Cutadapt^91^ v2.5 using a cutoff Phred score of 20 in NextSeq mode, and reads without any remaining bases were discarded (parameters: --nextseq-trim=20 -m 1 -a CTGTCTCTTATACACATCT).

### Transcriptome analysis

Processed reads were mapped to the human reference genome (NCBI RefSeq assembly GCF_000001405.40/GRCh38.p14; primary assembly and mitochondrion) using STAR^92^ v2.7.2b with default parameters except for including transcript annotations from RefSeq annotation version RS_2023_03 for GRCh38.p14. This annotation was also used to generate read counts on the exon level, summarized for each gene via featureCounts v1.6.4 from the Subread package^93^. Multi-mapping and multi-overlapping reads were counted unstranded with a fractional count for each alignment and overlapping feature (parameters: -s 0 -t exon -M -O --fraction). The resulting read counts were utilized to calculate TPM values and perform downstream analyses. DESeq2^94^ v1.24.0 was used to conduct principal component analysis by applying the DESeq function with “betaPrior=TRUE” to normalize the data, followed by variance stabilizing transformation by applying the vst function. The transformed data was plotted using the plotPCA function together with methods from the R package ggplot2 v3.3.2 (Supplementary Fig. 3B). In addition, sample-to-sample distances were calculated using the R dist function on vst-transformed counts and the resulting distance matrix was plotted using the R package pheatmap v1.0.12 (Supplementary Fig. 3C).

Transcriptomic analyses were conducted using TPM-normalized gene expression values derived from RNA sequencing. Gene expression patterns were visualized with heatmaps in GraphPad Prism and radar charts in Microsoft Excel.

Human Protein Atlas (HPA) single-cell transcriptomic data (version 23.0) were used as a reference dataset for in vivo gene expression profiles^34–36^. The HPA single-cell type atlas integrates and re-processes publicly available single-cell and single-nucleus RNA sequencing (scRNA-seq/snRNA-seq) datasets from healthy adult human tissues using a unified processing pipeline. Version 23.0 comprises expression profiles from 81 annotated cell types across 31 human tissues. The underlying datasets originate from publicly available repositories, including the Human Cell Atlas, Gene Expression Omnibus (GEO), Single Cell Expression Atlas, EMBL-EBI BioStudies, and Tabula Sapiens.

Normalized gene expression values (nCPM) were obtained from the Human Protein Atlas single-cell type data portal and the HPA file “rna_single_cell_type.tsv.zip” (available at: (https://www.proteinatlas.org/humanproteome/single+cell/single+cell+type/data#cell_type_data).

This file contains normalized transcript expression values for all annotated cell types, including Schwann cells and fibroblasts. Schwann cell and fibroblast expression profiles were extracted based on HPA cell-type annotations. Putative cell type-enriched marker genes were identified by calculating the Log2 fold change (Log2FC) between these two cell types, and selecting the eight genes with the highest Log2FC (fibroblast-enriched markers) and the eight genes with the lowest Log2FC (Schwann cell-enriched markers). These gene sets were subsequently applied to TPM-normalized pdSC transcriptomic data for comparative analyses. All selected genes are provided in Supplementary Data 1.

To assess similarity to fibroblast or Schwann cell phenotypes, mean TPM values of selected marker genes were visualized in heatmaps. Log2FC values between group averages were used to calculate a similarity score ranging from −10 to 10. Samples with Log2FC ≤ − 5 were classified as fibroblast-like, whereas those with Log2FC ≥ 5 were classified as Schwann cell-like (Fig. 3D and Supplementary Data 1).

Representative in vitro marker genes were defined using datasets from primary cultures of adult human peripheral nerve cells^37^. Identified Schwann cell- and fibroblast-related markers were mapped to our TPM data and visualized in heatmaps. Published scRNA-seq datasets of peripheral nerve-resident cell types were used as reference frameworks for Schwann cell subtype classification. From Zhang et al.^38^, marker genes for cell types identified in neonatal (P1) rat sciatic nerves and DRG were obtained from Supplementary Table 1 (“glia23928-sup-0001-TableS1.xlsx”), excluding the erythrocyte cluster. Marker genes for four Schwann cell subtypes were obtained from Supplementary Table 2 (“glia23928-sup-0002-TableS2.xlsx”). For each annotated cell type, the top twenty marker genes were selected by ranking genes within each cluster according to their reported average log2 fold-change (avg_logFC) values. From Gerber et al.^39^, we extracted differentially expressed marker genes from Supplementary File 3 (“elife-58591-supp3.xls”). Schwann cell markers were obtained from the “SmartSeq2 related to Figure 4” sheet, which contains clusters from mouse sciatic nerves spanning postnatal day 1 (P1) to adulthood (P60). Markers for additional cell types at P1 were obtained from the “10x_P1 related to Figure 6” sheet. Fibroblast markers were derived from the fibroblast-related cluster (FbRel*); the Schwann cell clusters and the proliferating fibroblast-like (prol. Fb) cluster were not included. Otherwise, cell types were defined according to the reported cluster annotations. For each cluster, the top twenty differentially expressed marker genes were selected by ranking genes within each cluster according to their reported average natural-log fold-change (avg_logFC) values.

These gene sets were used for comparative analysis of pdSC transcriptomes and do not represent direct biological equivalents to the human samples analyzed here. The TPM expression of these gene sets in pdSC samples was visualized using radar charts and heatmaps (Fig. 5 and Supplementary Data 1).

### Statistics and reproducibility

Statistical analyses were performed using SPSS Statistics 27 (IBM, Armonk, NY, USA). Normality of the data was assessed using the Shapiro–Wilk test. For normally distributed data, comparisons between two groups were performed using unpaired two-tailed t-tests, and correlations were assessed using Pearson’s correlation coefficient. For non-normally distributed data, comparisons between two groups were performed using the Mann–Whitney *U* test, and correlations were assessed using Spearman’s rank correlation coefficient. Two-way ANOVA was used for stimulation experiments involving two independent variables. Data were visualized using GraphPad Prism 9 (GraphPad Software, Inc., La Jolla, CA, USA).

For pdSC cultures, biological replicates were defined as cultures derived from independent donors. Six independent patient donors were included (*n* = 6 biological replicates). Commercial control Schwann cells (cSCs) originated from a single donor (*n* = 1 biological replicate). Where cells from different passages or repeated measurements of the same donor were included, these were considered repeated or technical measurements and not independent biological replicates. Sample sizes and the number of replicates used for individual experiments are indicated in the corresponding figure legends and “Methods” sections. Experiments without statistical analysis were performed using the biological and/or technical replicates specified for the respective experiment. Transcriptomic analyses were descriptive and exploratory, and no statistical inference was performed using technical replicates or repeated measurements as independent biological replicates.

### Reporting summary

Further information on research design is available in the Nature Portfolio Reporting Summary linked to this article.

## Supplementary information

Transparent Peer Review File

Supplementary Information

Description of Additional Supplementary Files

Supplementary Data 1

Reporting Summary

## Data Availability

A step-by-step technical description of the method presented in this article is available at protocols.io, https://doi.org/10.17504/protocols.io.36wgqnxj3gk5/v1. Raw human bulk RNA-seq data generated in this study have been deposited in the European Genome-phenome Archive (EGA) under accession number EGAS50000000737. All the numeric data used in Figs. 2–5 are included in the Supplementary Data 1.
