# Supplementary material for: Culture and gene expression profiling of Schwann cells from human sural nerve
Source: Commun Biol. 2026 Sep 28;9:1273. doi: 10.1038/s42003-026-11023-z (PMC13627658; doi:10.1038/s42003-026-11023-z)
Supplement: Supplementary file 1 [file 42003_2026_11023_MOESM1_ESM.pdf]

nature portfolio

Peer Review File

Culture and gene expression profiling of Schwann cells from human sural nerve

Corresponding Author: Dr Patricia García-Fernández

This file contains all reviewer reports in order by version, followed by all author rebuttals in order by version.

Version 0:

Reviewer comments:

Reviewer #1

(Remarks to the Author)
In this manuscript, the authors show that they are able to culture human Schwann Cells (SCs) from about 4 mm sural nerve biopsies and performed bulk RNA sequencing analyses to confirm SC identity and describe their phenotype.

There is enthusiasm for the presented possibility to culture SCs from such small biopsies as it allows to compare the supposed dysfunctional SCs derived from PNP patients with those from healthy controls to reveal potential differences and affected pathways. Unfortunately, such an analysis was not performed but the manuscript only focused on characterizing the pdSC cultures based on their bulk sequencing data.

There are serious concerns about the study that are listed below.

1. The study is presented as novel protocol to isolate human SCs from small biopsies, but actually uses well established tissue digests and culture conditions. Therefore, it rather represents an adaption of existing protocols (e.g. Weiss et al 2018; Peng et al 2020) for the culture of human SCs but from small biopsies.

2. There are key studies already characterizing cultured human SCs using proteome and transcriptome analyses (Weiss et al 2016; Peng et al 2020). The first one was not cited in this manuscript, although the RNA-sequencing analysis was performed in a similar way, including primary SCs and fibroblast as well as nerve fascicle samples and resulting phenotypes were described and also functionally validated. A comparison of the human SC expression profiles generated by this and the other studies would be very interesting to identify possible PNP associated differences in the isolated pdSCs.

3. The authors write in results line 173: “pdSCs are proliferating primary cells that can change their phenotype in culture and diverge from commonly understood SC found in vivo” or discussion line 234: “these results suggest that pdSC may be in a proliferative state rather than a myelinating state, and therefore lack the capacity to myelinate axons.”, which raises the question whether they are familiar with the repair phenotype of SCs (Jessen & Mirsky 2016 the repair Schwann cell; Jessen et al 2016 adaptive cellular reprogramming). As soon as SCs sense axon disruption or lose axon contact (such as upon nerve injury or isolation from the nerve environment), they will transform into repair cells. That repair phenotype is associated with the regain of a proliferative potential and with an upregulation of precursor/immature SC genes and repair associated genes (e.g. c-JUN see Arthur-Farraj et al 2012) as well as downregulation of genes involved in myelination. Thus, as soon as SCs are cultured, the SCs adapt a repair-associated cell state and significantly differ from adult SCs found in intact nerves (but still share the expression of the known SC lineage genes such as S100B, SOX10, …). Also in cultured nerve fascicles, the SCs within will adapt the repair phenotype after several days (see Weiss et al 2016).
Taken together, the authors report a well-defined SC phenotype of cultured human SCs expressing already reported genes and known to differ from adult SCs found in vivo (Monje P. 2020: Schwann cell cultures; Jessen & Mirsky 2016: the repair Schwann cell; Jessen et al 2016: adaptive cellular reprogramming; Weiss T. et al 2016: the human repair Schwann cell phenotype). However, it would be interesting to which extent the pdSCs show a repair SC-associated expression profile and if it is different to healthy SCs.

4. In the methods part, it is written, that the RNA-sequencing analysis was performed with 3 12wells of p3 pdSCs from 6 donors. This does not equal "n=18" as stated in the methods. “n” should indicate the donor number, which is 6, and from each donor, three 12wells of p3 pdSC cultures have been analyzed, this means, pdSCs from one donor have been

sequenced in triplicates. In turn, the cSC cultures of one donor (n=1 not 8), have been analyzed in p4, p5 and p6, this means, three replicates of cSCs from one donor have been sequenced. As a consequence, the statistical analysis of the sequencing data could have been performed in a wrong way and should be checked by a bioinformatician.

Other concerns:
a. The authors write several times, that their protocol allows the isolation of proliferative human SC “lines”. A cell line is defined by an unlimited replicative potential but the pdSCs have not been cultured beyond p3.
b. It is widely accepted amongst Schwann cell biologists that the commercial available human SCs have indeed less in common with fresh isolated SCs from human nerves. The cSC does not even express the SC lineage marker SOX10. Therefore, the label ‘human SC control’ should be questioned.
c. The methods part did not state where the human fibroblast culture was derived/purchased from. Indeed, human fibroblasts are as diverse as the tissue they are derived from and might differ from “nerve fibroblasts” (a collective term for ‘no SC’ including everything from endothelial cells, to pericytes, …). In addition, fibroblast cultures from at least 3 donors should have been included in the analysis.
d. In the last sentence of the abstract, it was written that the generated SC cultures could serve as tools for “therapeutic purpose”, which one would that be?
e. Was the immunostaining for SOX10 and S100B performed with all SC cultures of the 6 donors?
f. To prove whether the used antibodies are specifically targeting the desired protein, it is supportive to show a SC culture containing some fibroblasts to illustrate positive and negative SOX10 and S100B stainings side by side.
g. The Immunostaining of MBP is presumably not a specific staining because: 1) the MBP protein is downregulated in cultured human SCs (Weiss et al 2016; Peng et al 2020). Please note that the MBP gene can still be expressed, 2) the MBP staining looks exactly like the S100B staining. A similar staining pattern of different proteins could indicate an unspecific antibody binding and should be investigated.
h. In the discussion (line 276) it was written that a single-cell analysis would be necessary to determine the exact purity of the SC cultures. Actually, to define the purity of SC cultures, immunofluorescence stainings or flow cytometry against established SC markers would be sufficient.
i. In the discussion (line 290) pdSCs were proposed as “tool to study the pathophysiology of individual neuropathies, leading the way to personalized medicine”. This statement is exaggerated as distinct PNP associated SC phenotypes / expression profiles / functional differences have not been identified and the repair phenotype, that SCs gain after culture, will also influence such a potential PNP phenotype.

The authors have generated precious RNA-sequencing data of PNP patient derived SC cultures. It would be interesting to compare them to the already existing RNA sequencing data of human SC cultures and try to retrieve information about the differences between dysfunctional PNP-associated SCs and control SCs. Based on that information, functional differences might be identified that could be validated in pdSC cultures. Such findings would be of high interest for the SC community.

Reviewer #2

(Remarks to the Author)
The manuscript by García-Fernández et al. presents a methodology for isolating Schwann cells (SC) from small human sural nerve biopsy samples and a comprehensive transcriptome analysis to characterize their phenotype. This work represents a contribution to the field, particularly for studying patient-derived SC biology, which is critical for understanding PNS disorders. The study combines good experimental design with an in-depth analysis.

Suggestions for Additional Analysis:

qPCR Variability (Figure 2C): The relative expression of SC markers varies between the six patient-derived SCs. Could the authors comment on possible correlations between expression levels and patient characteristics such as age, disease duration, or diagnosis? Moreover, is there a correlation between the expression levels of the three markers (SOX10, S100B, and MBP) within the same patient samples? Differentiating the dots in the graph and/or providing a supplementary table with the raw data would help.

Purity Analysis: While the study clearly confirms the SC phenotype through transcriptome analysis, including a quantification of SOX10-negative nuclei in the cultures would strengthen the purity assessment. Additionally, staining for a fibroblast-specific marker could provide more insight into potential contamination levels.

Supplementary Figure 2: This figure includes 18 samples which from the methods they represent triplicates at P3, please indicate in the graph which are triplicates (do they cluster together in the PCA plot?) and/or include in a supplementary table the PCA values for triplicates. This would help in understanding the reproducibility of the protocol across replicates.

Version 1:

Reviewer comments:

Reviewer #1

(Remarks to the Author)
The authors’ efforts to revise the manuscript according to the reviewers’ suggestions are appreciated. The idea and potential

significance of this work are acknowledged and valued. Nevertheless, in its current form, the experimental evidence does not support the main conclusions drawn by the authors.

The authors write: "The data show that the culture of human SC obtained with this method presents a good preservation of SC phenotypes and is highly reproducible when applied to small biopsy samples from different patients." Unfortunately, the presented data do not support this statement. Immunofluorescence images show stainings for S100 and SOX10, that identified SCs in the pdSC cultures, but they also show that pdSC cultures contained different amounts S100/SOX10 negative cells (Fig.2B, S.Fig.2A). These SOX10 negative cells are not SCs, most likely, they represent fibroblasts. Bulk RNA sequencing performed on these cultures will inevitably yield a mixed gene expression profile, reflecting both SC and fibroblast populations. SC-specific transcripts will still be detected; however, their relative abundance decreases proportionally with the proportion of fibroblasts in the culture. Accordingly, the RNA sequencing data show that triplicates from one donor were negative for SOX10, and other SC markers were barely detected, whereas triplicates from another donor exhibited strong SOX10 expression, which correlated with the presence of additional SC-associated genes (see triplicates in Fig. 3 and Fig. 4). Indeed, fibroblast-associated genes were also present in the pdSC cultures, as shown in Fig. 4 and Fig. 5. Nevertheless, the authors concluded that "… the expression of fibroblast markers seemed to be prominent in pdSC, indicating a fibroblast-like phenotype." while never considering that this signature is just a results of a contaminating fibroblast population. Even though their own immunofluorescence stainings and analyses show that pdSC cultures are not pure, the authors write that "In addition, the homogeneity of the culture observed in our immunostaining analyses suggested the elimination of other contaminating cells, such as fibroblasts, in the process."

A more thorough evaluation of the results and a more cautious interpretation are necessary to ensure that the conclusions accurately reflect the presented data.

Please find the new reviewer comments below the author comments.

Reviewers' Comments to the Authors:
Reviewer #1:
In this manuscript, the authors show that they are able to culture human Schwann Cells (SCs) from about 4 mm sural nerve biopsies and performed bulk RNA sequencing analyses to confirm SC identity and describe their phenotype.
There is enthusiasm for the presented possibility to culture SCs from such small biopsies as it allows to compare the supposed dysfunctional SCs derived from PNP patients with those from healthy controls to reveal potential differences and affected pathways. Unfortunately, such an analysis was not performed but the manuscript only focused on characterizing the pdSC cultures based on their bulk sequencing data.
Author response: Thank you for appreciating the importance of our study and identifying one of its main limitations. Nerve biopsies are obtained through an invasive procedure that is only performed in patients for diagnostic purposes. This unfortunately means that we were not able to obtain SCs from healthy donors as controls and thus perform the analysis suggested by the reviewer. Commercially available SCs were used instead as the best available control. This limitation was expanded in the discussion in page 13, line 297-300, "In addition, due to the invasive nature of nerve biopsy, obtaining nerve samples from healthy volunteers was not ethically permissible. Under other circumstances, the isolation of Schwann cells from healthy donors could help clarify the contribution of neuropathy to the characteristics of each cell strain.".
Reviewer Response: There seems to have been a misunderstanding. It was suggested to compare the pdSC samples with publicly available RNA sequencing data from healthy human Schwann cells, not to obtain new biopsies from healthy donors for additional analysis.
When using commercially available "SCs", their SC identity has to be confirmed before using it as a control. By looking at the stainings and RNA sequencing data, the cSCs are negative for established SC markers. Thus, they cannot serve as a proper SC control.

There are serious concerns about the study that are listed below.
1. The study is presented as novel protocol to isolate human SCs from small biopsies, but actually uses well established tissue digests and culture conditions. Therefore, it rather represents an adaption of existing protocols (e.g. Weiss et al 2018; Peng et al 2020) for the culture of human SCs but from small biopsies.
Author response: We absolutely agree with giving previous protocols the acknowledgement that they deserve for their contribution in inspiring this method. However, our protocol is not a direct modification of one or several of them, in particular not Weiss et al 2018 or Pent et al 2020, since we do not use any of the steps followed in those protocols, such as magnetic separation, FACS or two-step enrichment exploiting differential adhesion properties. Nevertheless, we have indeed adapted the D-valine media and protocol used in Kaewkhaw, R., A.M. Scutt, and J.W. Haycock, Integrated culture and purification of rat 510 Schwann cells from freshly isolated adult tissue. Nat Protoc, 2012.7(11): p. 1996-2004 to obtain SCs from smaller human material than had ever been tested before. We believe that this concept is well explained in the main text, particularly in page 2, lines 41 to 57.
Reviewer Response: There seems to have been a misunderstanding. The similarity with published protocols is the used dissociation solution for fascicle tissue digest and culture conditions, meaning the culture medium supplemented with FGF-basic and PDGF. It is correct that the D-valine medium was so far only described for rodent SC culture. Nevertheless, the presented approach appears to be an adaptation of several previously established SC isolation methods, rather than a novel protocol.

2. There are key studies already characterizing cultured human SCs using proteome and

transcriptome analyses (Weiss et al 2016; Peng et al 2020). The first one was not cited in this manuscript, although the RNA-sequencing analysis was performed in a similar way, including primary SCs and fibroblast as well as nerve fascicle samples and resulting phenotypes were described and also functionally validated. A comparison of the human SC expression profiles generated by this and the other studies would be very interesting to identify possible PNP associated differences in the isolated pdSCs.
Author response: Thank you for bringing this study to our attention, we have included a citation of this article in the discussion, page 11, line 217 (reference 43). We absolutely agree that a comparison between our analysis and other published ones would be of great interest. Unfortunately, due to the size limitations of this article, we had to focus on the studies that we considered most relevant. The RNA-sequencing analysis performed in Weiss et al 2016 focuses on markers related to repair phenotype, debris clearance, phagocytosis, antigen presentation or T-cell related signaling. While interesting, our manuscript focuses on Schwann cell identity, and we will use future studies to focus on other signaling pathways or pathogenic phenotypes. Since the RNA-sequencing data is available, we also invite other authors to perform whichever comparisons may be more relevant or interesting for them.

3. The authors write in results line 173: “pdSCs are proliferating primary cells that can change their phenotype in culture and diverge from commonly understood SC found in vivo” or discussion line 234: “these results suggest that pdSC may be in a proliferative state rather than a myelinating state, and therefore lack the capacity to myelinate axons.”, which raises the question whether they are familiar with the repair phenotype of SCs (Jessen & Mirsky 2016 the repair Schwann cell; Jessen et al 2016 adaptive cellular reprogramming). As soon as SCs sense axon disruption or lose axon contact (such as upon nerve injury or isolation from the nerve environment), they will transform into repair cells. That repair phenotype is associated with the regain of a proliferative potential and with an upregulation of precursor/immature SC genes and repair associated genes (e.g. c-JUN see Arthur-Farraj et al 2012) as well as downregulation of genes involved in myelination. Thus, as soon as SCs are cultured, the SCs adapt a repair-associated cell state and significantly differ from adult SCs found in intact nerves (but still share the expression of the known SC lineage genes such as S100B, SOX10, …). Also in cultured nerve fascicles, the SCs within will adapt the repair phenotype after several days (see Weiss et al 2016). Taken together, the authors report a well-defined SC phenotype of cultured human SCs expressing already reported genes and known to differ from adult SCs found in vivo (Monje P. 2020: Schwann cell cultures; Jessen & Mirsky 2016: the repair Schwann cell; Jessen et al 2016: adaptive cellular reprogramming; Weiss T. et al 2016: the human repair Schwann cell phenotype). However, it would be interesting to which extent the pdSCs show a repair SC-associated expression profile and if it is different to healthy SCs.
Author response: We absolutely agree with the reviewer about the interest of exploring further the phenotype of pdSC and appreciate the clear and detailed information about the SC repair phenotype. We are aware that a repair phenotype is associated with the regain of a proliferative potential and could in theory match the proliferative capacity of our Schwann cells in culture. In particular, the proliferative phenotype described in our article is also present in cSC (as shown in Figure 5 C) and thus seems to characterize cultured human SCs in general, as mentioned by the reviewer. We agree that it would be particularly interesting to study the SC repair phenotype and other pathological ones related to axonal disruption, damage or neuropathy. This manuscript aims to describe only the isolation and culture method of pdSC and the phenotype resulting from the culture. However, we are currently working on a deeper analysis between pdSC and cSCs, as well as among patients, which will be included in a follow-up article. This analysis will further inform on the repair and pathological phenotypes of each patient-derived SC strain.
Reviewer response: Please note that it is published and well known that SCs enter a proliferative state and downregulate myelin-related genes when cultured. The sentence in line 245: “These results, along with our analyses of the different Schwann cell phenotypes and the observed proliferation in culture, suggest that pdSC may be in a proliferative state rather than in a myelinating state, and therefore lack the capacity to myelinate axons.” implies that this observation is novel, although it reflects a well-established phenomenon of cultured SCs.
It is true that the commercial “SCs” used as control also proliferated in culture. However, so would most other cultured human cell types.

4. In the methods part, it is written, that the RNA-sequencing analysis was performed with 3 12wells of p3 pdSCs from 6 donors. This does not equal "n=18" as stated in the methods. “n” should indicate the donor number, which is 6, and from each donor, three 12wells of p3 pdSC cultures have been analyzed, this means, pdSCs from one donor have been sequenced in triplicates. In turn, the cSC cultures of one donor (n=1 not 8), have been analyzed in p4, p5 and p6, this means, three replicates of cSCs from one donor have been sequenced. As a consequence, the statistical analysis of the sequencing data could have been performed in a wrong way and should be checked by a bioinformatician.
Author response: The reviewer makes a very fair point noticing the limitations in n number and availability of donors. As mentioned in the previous question, the aim of this manuscript is to prove the identity of SCs isolated with the described method and their phenotype in culture. Thus, the individual differences between each patient-derived cell strain or between passages is not our main interest. Therefore, in our pdSC and cSC data set we have included all biological and technical replicates as part of the total n number in order to have a clearer vision of the common characteristic of all control and patient-derived SCs when cultured with this method. In future studies where we deepen into individual differences, we do cluster technical replicates as suggested by the reviewer.
Reviewer response: There seems to have been a misunderstanding. The presented n number of 6 is not a limitation of this study. However, defining triplicates from one donor as n = 3 is methodologically incorrect, as these do not represent independent biological replicates. This should be clarified in the methods section and in the respective figure legend to

avoid misinterpretation of sample numbers and statistical validity.

Other concerns:

a. The authors write several times, that their protocol allows the isolation of proliferative human SC “lines”. A cell line is defined by an unlimited replicative potential but the pdSCs have not been cultured beyond p3.
Author response: The reviewer is absolutely right. We’d like to apologize for the miswording. We have rephrased the word ‘lines’ or have removed it from the text.

b. It is widely accepted amongst Schwann cell biologists that the commercial available human SCs have indeed less in common with fresh isolated SCs from human nerves. The cSC does not even express the SC lineage marker SOX10. Therefore, the label ‘human SC control’ should be questioned.
Author response: We agree with the reviewer that a primary culture of SCs, as are pdSCs, show more Schwann cell characteristics than commercially available human SCs, as we have proved in this manuscript. Unfortunately, as mentioned in the discussion and in our first answer, we were not able to obtain SCs from healthy donors as controls. Thus we were limited to the use of commercial SCs in order to prove the identity of our cells, which we further confirmed through comparisons with reference data sets. This very good point highlights the importance of our described protocol, which eases and widens their isolation and primary culture, leading the way to personalized medicine, while opening new possibilities for better commercially available SCs.
Reviewer response: In order to prove the identity of pdSCs, the authors are not forced to use commercial SCs (with limited to non-existing SC characteristics), but should compare their pdSC with available data sets of characterized human SCs.

c. The methods part did not state where the human fibroblast culture was derived/purchased from. Indeed, human fibroblasts are as diverse as the tissue they are derived from and might differ from “nerve fibroblasts” (a collective term for ‘no SC’ including everything from endothelial cells, to pericytes, …). In addition, fibroblast cultures from at least 3 donors should have been included in the analysis.
Author response: This fibroblast sample was isolated from a skin biopsy of a healthy volunteer and has been previously described in Karl et al, 2019; Kress et al, 2021 and Egenolf et al, 2021 [1-3]. We have included this information in the method section, in page 16, lines 384-389, “As control of a fibroblast cell type, a sample isolated from a skin biopsy of a healthy volunteer and previously described [78-80] was used. In summary, a 6-mm skin punch biopsy from the calf was cut into small dermal pieces and placed in fibroblast medium (DMEM/F-12 with 10% FCS and 1% penicillin/streptomycin) for 1hr to allow tissue adherence before adding additional medium. Cultures were maintained with medium changes every 2–3 days until fibroblast outgrowth, and cells were expanded and further processed at passage four”, including the three references in the text. We agree with the reviewer that the inclusion of one fibroblast sample may not be enough to perform a full statistical and robust analysis. In this manuscript, we aimed to include this one sample in order to have a clearer vision of the differences between SC and fibroblast cell types, and better identify pdSC.

d. In the last sentence of the abstract, it was written that the generated SC cultures could serve as tools for “therapeutic purpose”, which one would that be?
Author response: As mentioned at the end of the discussion, we follow the idea that the isolation of pdSC may be used to investigate the proinflammatory, pro- or demyelinating phenotypes to which Schwann cells may contribute. This may help to improve treatment options for individual patients.
Reviewer response: This should be clarified in the manuscript because the sentence “…serve as tools for diagnostic and therapeutic purposes” could be misunderstood in that way, that the intention is to use the pdSCs as therapy.

e. Was the immunostaining for SOX10 and S100B performed with all SC cultures of the 6 donors?
Author response: The immunostaining for SOX10, S100B and MBP was initially performed with 2 out of the 6 donors. However, we realized during the revision process that it would be beneficial to include the immunostainings from all donors, including the fibroblast cell line and the commercially available SC used as control. This can now be found in Figure 2 B and as an additional supplementary figure (Supplementary figure 2). The new data was further explained in the text, in page 4, line 93 to 99, “The immunofluorescence staining showed consistent expression of SOX10 and S100B in pdSC, with a lower expression in cSC and in a reference sample from a human fibroblast culture (Fibroblast_1) (Fig. 2B). This expression was heterogeneous among pdSC strains isolated from different patients (Suppl. Fig. 2 A). In addition, the MBP marker showed low and unspecific results in all samples (Suppl. Fig. 2 A). Moreover, we compared the expression of Fibronectin 1 (FN1), a marker commonly associated with fibroblasts, among cSC, pdSC and Fibroblast_1 (Suppl. Fig. 2 B). While results showed a similar expression among all samples, differences in morphology between Schwann cells (pdSC) and fibroblasts (Fibroblast_1) can be clearly identified (Fig. 2B)” and in methods, section “Immunocytochemistry”, page 16, line 392 and 399.
Reviewer response: The additional stainings are appreciated. They show that pdSC cultures contain a significant amount of cells that are negative for established SC markers SOX10 and S100B. The most obvious conclusion would be, that the cultures contain different amounts of fibroblasts but the authors never commented about the fibroblast contamination in their pdSC cultures.
The FN1 staining shows that FN1 is stronger expressed in pdSCs than in the fibroblasts. Why did the authors not use Thy.1 as a fibroblast marker?

f. To prove whether the used antibodies are specifically targeting the desired protein, it is supportive to show a SC culture containing some fibroblasts to illustrate positive and negative SOX10 and S100B staining side by side.
Author response: As mentioned above, we have now included this immunostaining in Figure 2 B and as an additional supplementary figure (Supplementary figure 2).

g. The Immunostaining of MBP is presumably not a specific staining because: 1) the MBP protein is downregulated in cultured human SCs (Weiss et al 2016; Peng et al 2020). Please note that the MBP gene can still be expressed, 2) the MBP staining looks exactly like the S100B staining. A similar staining pattern of different proteins could indicate an unspecific antibody binding and should be investigated.
Author response: The reviewer makes a fair point questioning the specificity of the MBP antibody. The antibody used in this study was a monoclonal rat antibody to MBP #ab7349, Abcam, RRID:AB_305869, which had been used in over 308 publications (https://www.abcam.com/en-us/products/primary-antibodies/myelin-basic-protein-antibody-12-oligodendrocyte-marker-ab7349?srsltid=AfmBOoppU6GUDtC2U5W_0Bjf44hIP-lju1NkbbbTDxaHH3fW6s3M1fi7), thus promising good specificity and results. However, as mentioned above, we repeated and expanded the immunostainings during this revision, including MBP. We agree that the results show a low specificity, possibly due to the low expression in all samples. Thus, we removed it from the main text (Figure 2) and included it only in Supplementary Figure 2.

h. In the discussion (line 276) it was written that a single-cell analysis would be necessary to determine the exact purity of the SC cultures. Actually, to define the purity of SC cultures, immunofluorescence stainings or flow cytometry against established SC markers would be sufficient.
Author response: The reviewer makes a good and fair suggestion, that would maybe help clarify the purity of the culture. However, our study has also proved that pdSC shows a fibroblast-like phenotype and can share proliferative markers with other cell types, as well as lower expression of Schwann cell markers depending on the strain. Thus, although immunofluorescence staining and flow cytometry may help differentiate cell types with clear and different phenotypes, a complete single-cell transcriptomic would be necessary to fully understand the different cell types and phenotypes they present. In addition, since the SC cultures are of multicellular origin, the advantage of this strategy is that it better reflects the natural variability of Schwann cell phenotypes. In contrast, selecting specific Schwann cell line clones would likely limit the experimental and clinical potential of the cultures by reducing phenotypic diversity.
Reviewer response: If the purity of pdSC cultures is not clarified, then the authors don't know if the 'fibroblast-like' phenotype they describe for their pdSC cultures is just a results of a contaminating fibroblast population.
In addition, propagating single SC "clones" will face the challenge of the limited live span of cultured human cells and the acquisition of a senescent phenotype, thus, multiple passaged human SCs will likely diverge from their original phenotype and not be useful for functional studies. How will the authors address that?

i. In the discussion (line 290) pdSCs were proposed as "tool to study the pathophysiology of individual neuropathies, leading the way to personalized medicine". This statement is exaggerated as distinct PNP associated SC phenotypes / expression profiles / functional differences have not been identified and the repair phenotype, that SCs gain after culture, will also influence such a potential PNP phenotype.
Author response: The reviewer is absolutely right pointing out that the phenotype gained in culture may influence and mask the potential PNP phenotype. Nevertheless, the genetic study of each cell strain and their behavior upon different stimuli, as well as in different culture conditions or passages, can elucidate the PNP-related characteristics of that specific patient. We are at the moment working on analyzing the individual differences among cell strains and their correlations with the neuropathic symptoms seen in each patient. Furthermore, in the near future, we see the potential of adding pdSC to induced human sensory neurons, which are produced in our lab as well, and study their interaction and relationship.
Reviewer response: It is a meaningful goal to compare the individual differences between the pdSC cultures and associations to neuropathic syndromes and the myelination potential towards iPSC-derived sensory neurons. Because your work is so important, the purity of pdSC cultures should be very high to draw relevant conclusions.

The authors have generated precious RNA-sequencing data of PNP patient derived SC cultures. It would be interesting to compare them to the already existing RNA sequencing data of human SC cultures and try to retrieve information about the differences between dysfunctional PNP-associated SCs and control SCs. Based on that information, functional differences might be identified that could be validated in pdSC cultures. Such findings would be of high interest for the SC community.
Author response: We would like to thank you for the very helpful contribution that your comments have provided to our manuscript. In this article, we have already compared our results with existing RNA sequencing data of human SC (Human Protein Atlas[Ref 34]; Karlsson et al, 2021[Ref 35]; Peng et al, 2020[Ref 36]; Gerber et al; 2021[Ref 37] and Zhang et al, 2021[Ref 38]) in figures 3, 4 and 5. In addition, we also hope that our future studies on individualized PNP patient derived SCs and their PNP-associated phenotypes will indeed be of great interest for the SC community
Reviewer response: The single-cell type transcriptomics map of human tissue does not include nerve tissue. How were the SC_HPA and fibroblast_HPA cell specific markers identified?

Other:
Line 48: "Fibroblasts do not express the D-amino acid oxidase (DAAO) required to use up D-valine and therefore survive in

this particular medium. This results in their elimination and promotes a pure SC culture." If it is true, that fibroblast shall not survive in this medium, why are most pdSCs contaminated with a significant amount of S100-/SOX10- cells as shown in the IF stainings?

Line 90-91: "SC typically express specific markers like …, and the myelin binding protein (MBP)." This statement is not true because only in myelinating SCs express MBP. However, upon isolation, myelinating SCs downregulate MBP and other myelin genes because they adapt a repair phenotype.

Reviewer #2

(Remarks to the Author)
The authors have responded all issues raised. This is an important work for the field.

Version 2:

Reviewer comments:

Reviewer #1

(Remarks to the Author)
Dear authors,

Thank you for addressing most of the points. Again, be ensured that this work is acknowledged and valued. You demonstrated that it is possible to culture Schwann cells from very small nerve biopsies as the immunofluorescence staining showed SOX10 and S100B double positive Schwann cells in the cultures. However, the nerve biopsy cultures also contained SOX10/S100B negative cells, some cultures more, some less. Based on the absence of established Schwann cell markers and the fibroblast-like morphology, the data do not support the interpretation that these cells represent Schwann cells with a fibroblast phenotype. Instead, the findings are more consistent with a contaminating fibroblast population.

The argument that "All cultures were processed following the same protocol and cultured in the same medium, as well as the commercial SC used as control, thus suggesting that the differences we see are based on the origin of the biopsy and not the protocol itself." does not hold because using the same protocol and culture conditions does not exclude differences in cellular composition between samples. Variability in the proportion of Schwann cells versus contaminating cells can arise from differences in the cellular composition or origin of the original biopsy, Schwann cell viability (due to the PNP), donor-dependent variabilities, or selective expansion during culture. This is why a systematic assessment of Schwann cell culture purity is commonly performed when working with cultured Schwann cells.
Concerning the assessment of SC purity the authors write "single-cell analyses would be necessary to determine the exact purity of the SC culture and verify the absence of other cell types". While single-cell RNA sequencing can provide high-resolution insights into cellular heterogeneity, it is not a prerequisite for determining Schwann cell culture purity. The presence of Schwann cells can be evaluated using standard immunostainings, including the SC marker-based immunostaining against SOX10 and S100B as the authors performed. Those stainings show that the different nerve biopsy cultures contain varying amounts SOX10 and S100B negative cells. SOX10 is a core lineage transcription factor of SCs present in immature, mature and repair SCs; its absence in Schwann cells is unlikely.
Nevertheless, the authors assume that their isolation results in pdSC cultures composed of different Schwann cell phenotypes, including a dominant fibroblast-like phenotype, rather than a much likelier explanation: a contaminating fibroblast population. The statement: "Overall, our data indicate that the cultures contain Schwann cells with donor-dependent phenotypic variability rather than fibroblast contamination." is in fact not demonstrated by the data. The presented data show that the nerve biopsy cultures from different donors contain different amounts of SOX10/S100B positive Schwann cells. This is also nicely reflected in the bulk sequencing data. Then, the expression profiles of pdSC cultures are compared to different Schwann cell and fibroblast associated markers derived from different sources but all showing the same, dependent on the donor, some nerve biopsy cultures have more markers in common with Schwann cells, some have more markers in common with fibroblasts. While these data would usually suggest that there are different amounts of fibroblasts present in the pdSC cultures, the authors conclude that the established nerve biopsy cultures represent Schwann cells with a fibroblast phenotype "supported by the fact that the commercial SCs (cSC) also show low expression of these markers in both our IF stainings and RNAseq". In turn, it is not questioned that commercially available Schwann cell cultures show that strong resemblance with fibroblasts.

It all ends in one question: how would the data look like if the pdSC cultures would contain different amounts of contaminating fibroblasts? The answer is: exactly the same. Thus, the authors cannot discriminate if the isolated cells are Schwann cells with a fibroblast phenotype or a mixture of Schwann cells and fibroblasts.

Other:
The authors state that "Fibroblasts (Fibroblast_HPA) and SC (SC_HPA) single-cell transcriptomic consensus from Human Protein Atlas version 23.0 (HPA), obtained from 31 different tissues were compared in order to find in vivo specific markers for each cell type". However, the Human Protein Atlas does not provide a curated or validated consensus Fibroblast or Schwann cell gene signature. It remains unclear which tissue, dataset, cell clusters, and selection criteria were used to define this signature. Please clarify this in the methods part.

The Thy.1 staining, while appreciated, looks very strange because Thy.1 should be expressed at the cell membrane but shows a nuclear staining. How do the authors explain this?

One reviewer comment about the n number was not addressed: There seems to have been a misunderstanding. The presented n number of 6 is not a limitation of this study. However, defining triplicates from one donor as n = 3 is methodologically incorrect, as these do not represent independent biological replicates. This should be clarified in the methods section and in the respective figure legend to avoid misinterpretation of sample numbers or statistics.

Version 3:

Reviewer comments:

Reviewer #1

(Remarks to the Author)
Dear authors,

The revised wording helps to introduce the hypothesis that a varying population of SOX10⁻/S100B⁻ Schwann cells may exist in neuropathic sural nerves, or that Schwann cells isolated from such tissue may adopt a SOX10-/S100B- fibroblast-like phenotype in culture, concepts that are not yet established.

However, the manuscript would need to be revised accordingly, as several sections still imply the generation of a pure, bona fide Schwann cell culture using this method.

Several methodological aspects still need to be clarified, including the correct use of “n”-numbers, how the different “Schwann cell subtypes” were defined and extracted from the published scRNA-seq datasets, and how the curated or validated consensus fibroblast and Schwann cell gene signatures were derived from the Human Protein Atlas (see points below).

If the authors conclude that the initial isolation yields a pure Schwann cell population that subsequently undergoes pronounced reprogramming toward a fibroblast-like phenotype in culture, the culture conditions should be optimized to prevent or minimize this effect. Otherwise, it remains questionable whether these cultures can serve as a reliable tool for studying neuropathy pathophysiology and for advancing personalized medicine approaches.

Finally, the question of how the data would appear if the pdSC cultures contained varying proportions of contaminating fibroblasts remains a central point of concern. The current data do not allow discrimination between Schwann cells adopting a fibroblast-like phenotype and a mixed population of Schwann cells and fibroblasts. Therefore, careful wording and balanced argumentation are essential when introducing this hypothesis.

1. To strengthen the title while maintaining transparency regarding the heterogeneous cell populations, the following title is suggested: “Transcriptomic profiling reveals heterogeneity in primary cultures derived from human polyneuropathic sural nerve biopsies”. This title would provide room to address the authors’ hypothesis of a not yet established view that Schwann cells may adopt a fibroblast-like phenotype in culture.

2: Even though the authors the authors know of all the limitations, they write that “In this study, we present a novel protocol to culture human SC from ~4-mm diagnostic sural nerve biopsies. Cells isolated with this method were characterized and expanded, in order to create well-defined primary Schwann cell cultures derived from patients with PNP that could serve as tools for diagnostic and therapeutic purposes. The application of this protocol resulted in the establishment of a culture of spindle-shaped cells that tested positive for the transcription factor SOX10 in their nuclei, and for the proteins S100B and MBP in their cytoplasm, resembling previously described SC hallmarks [39-43]. The analysis of their transcriptome confirmed the expression of S100B and SOX10, but not MBP, in cells cultured with this method.”
or
“In this study, we describe a protocol that incorporates key elements of previously published culture conditions while enabling the successful use of D-valine medium to isolate and expand a pure Schwann cell culture from ~4 mm diagnostic human sural nerve biopsies.”
These paragraphs are misleading as it suggests the culture of pure, SOX10+/S100B+ Schwann cell cultures.

3. The description of sample size is still misleading. While the authors state that Schwann cell cultures were derived from 6 independent donors (n = 6 biological replicates), they also indicate that technical replicates and cells from different passages were included in the total n-number. Technical replicates and repeated measurements from the same donor (e.g. different passages) do not constitute independent biological replicates and should not be included in the reported n. This approach artificially inflates sample size and may lead to incorrect statistical inference. The authors should clearly distinguish between biological replicates, technical replicates, and repeated measurements in every figure and revise any statistical analysis accordingly.

4. The Human Protein Atlas does not provide a curated or validated consensus Fibroblast or Schwann cell gene signature. It remains unclear which tissue, dataset, cell clusters, and selection criteria were used to define this signature. The revised methods part does not include that information.

In addition, the methods part does not contain how the genes for the different “SC subtypes” have been selected. The authors wrote “To identify the distinct phenotype of pdSC, markers from previous studies performing single-cell RNA sequencing of peripheral nerve specimens were analyzed. From two reference data sets [37, 38], twenty genes corresponding to each SC subtype were selected and their TPMs in pdSC were represented in radar charts (Fig. 5A, B, Suppl. Table 3).” In Zhang et al, they defined SC subpopulations of day 1 of age neonatal Sprague–Dawley rats. In Gerber et al, the extracted nerve cells from newborn mice at P1, and cells at a young adult stage at P60. The authors did not specify from which datasets the “SC subtypes” were extracted. In addition, those publications use newborn or young naïve nerves as source. With regard to the patient derived data, it would be more appropriate to use scRNA-seq data from older rodents and might also involve an injury context, e.g: Lovatt D, Tamburino A, Krasowska-Zoladek A, Sanoja R, Li L, Peterson V, Wang X, Uslaner J. scRNA-seq generates a molecular map of emerging cell subtypes after sciatic nerve injury in rats. Commun Biol. 2022 Oct 19;5(1):1105. doi: 10.1038/s42003-022-03970-0. PMID: 36261573; PMCID: PMC9581950.

Open Access This Peer Review File is licensed under a Creative Commons Attribution 4.0 International License, which permits use, sharing, adaptation, distribution and reproduction in any medium or format, as long as you give appropriate credit to the original author(s) and the source, provide a link to the Creative Commons license, and indicate if changes were made.
In cases where reviewers are anonymous, credit should be given to 'Anonymous Referee' and the source.
The images or other third party material in this Peer Review File are included in the article’s Creative Commons license, unless indicated otherwise in a credit line to the material. If material is not included in the article’s Creative Commons license and your intended use is not permitted by statutory regulation or exceeds the permitted use, you will need to obtain permission directly from the copyright holder.
To view a copy of this license, visit https://creativecommons.org/licenses/by/4.0/

Response to Reviewers

Manuscript COMMSBIO-24-7498-T

Dear Dr. Karlsson Rosenthal,

Thank you for giving us the opportunity to submit a revised draft of the manuscript "Isolation, culturing, and gene expression profiling of Schwann cells from human sural nerve" for publication in the Journal of Communications Biology. We appreciate the time and effort that you and the reviewers dedicated to providing feedback on our manuscript and are grateful for the insightful comments on and valuable improvements to our paper. We have incorporated most of the suggestions made by the reviewers. Those changes are highlighted within the manuscript. Please see below, in blue, for a point-by-point response to the reviewers' comments and concerns. All page numbers refer to the revised manuscript file with changes highlighted in yellow.

In addition, three new coauthors have been added to the manuscript during this revision: Dr. Eva Meller, representative of the Department of Neurosurgery, University Hospital of Würzburg, responsible for the performance of the nerve biopsies and collection of samples necessary for the study; and Dr. Julia Grüner and Maximilian Koch, from the Department of Neurology, University Hospital of Würzburg, who performed the necessary experiments and immunofluorescence staining included in this revision and contributed to the creation of the new and modified figures.

Reviewers' Comments to the Authors:

Reviewer #1:

In this manuscript, the authors show that they are able to culture human Schwann Cells (SCs) from about 4 mm sural nerve biopsies and performed bulk RNA sequencing analyses to confirm SC identity and describe their phenotype.

There is enthusiasm for the presented possibility to culture SCs from such small biopsies as it allows to compare the supposed dysfunctional SCs derived from PNP patients with those from healthy controls to reveal potential differences and affected pathways. Unfortunately, such an analysis was not performed but the manuscript only focused on characterizing the pdSC cultures based on their bulk sequencing data.

Author response: Thank you for appreciating the importance of our study and identifying one of its main limitations. Nerve biopsies are obtained through an invasive procedure that is only performed in patients for diagnostic purposes. This unfortunately means that we were not able to obtain SCs from healthy donors as controls and thus perform the analysis suggested by the reviewer. Commercially available SCs were used instead as the best available control.

This limitation was expanded in the discussion in page 13, line 297-300, "*In addition, due to the invasive nature of nerve biopsy, obtaining nerve samples from healthy volunteers was not ethically permissible. Under other circumstances, the isolation of Schwann cells from healthy donors could help clarify the contribution of neuropathy to the characteristics of each cell strain.*".

There are serious concerns about the study that are listed below.

1. The study is presented as novel protocol to isolate human SCs from small biopsies, but actually uses well established tissue digests and culture conditions. Therefore, it rather represents an adaption of existing protocols (e.g. Weiss et al 2018; Peng et al 2020) for the culture of human SCs but from small biopsies.

Author response: We absolutely agree with giving previous protocols the acknowledgement that they deserve for their contribution in inspiring this method. However, our protocol is not a direct modification of one or several of them, in particular not Weiss et al 2018 or Pent et al 2020, since we do not use any of the steps followed in those protocols, such as magnetic separation, FACS or two-step enrichment exploiting differential adhesion properties. Nevertheless, we have indeed adapted the D-valine media and protocol used in *Kaewkhaw, R., A.M. Scutt, and J.W. Haycock, Integrated culture and purification of rat 510 Schwann cells from freshly isolated adult tissue. Nat Protoc, 2012. 7(11): p. 1996-2004* to obtain SCs from smaller human material than had ever been tested before. We believe that this concept is well explained in the main text, particularly in page 2, lines 41 to 57.

2. There are key studies already characterizing cultured human SCs using proteome and transcriptome analyses (Weiss et al 2016; Peng et al 2020). The first one was not cited in this manuscript, although the RNA-sequencing analysis was performed in a similar way, including primary SCs and fibroblast as well as nerve fascicle samples and resulting phenotypes were described and also functionally validated. A comparison of the human SC expression profiles generated by this and the other studies would be very interesting to identify possible PNP associated differences in the isolated pdSCs.

Author response: Thank you for bringing this study to our attention, we have included a citation of this article in the discussion, page 11, line 217 (reference 43). We absolutely agree that a comparison between our analysis and other published ones would be of great interest. Unfortunately, due to the size limitations of this article, we had to focus on the studies that we considered most relevant. The RNA-sequencing analysis performed in Weiss et al 2016 focuses on markers related to repair phenotype, debris clearance, phagocytosis, antigen presentation or T-cell related signaling. While interesting, our manuscript focuses on Schwann cell identity, and we will use future studies to focus on other signaling pathways or pathogenic phenotypes. Since the RNA-sequencing data is available, we also invite other authors to perform whichever comparisons may be more relevant or interesting for them.

3. The authors write in results line 173: “pdSCs are proliferating primary cells that can change their phenotype in culture and diverge from commonly understood SC found in vivo” or discussion line 234: “these results suggest that pdSC may be in a proliferative state rather than a myelinating state, and therefore lack the capacity to myelinate axons.”, which raises the question whether they are familiar with the repair phenotype of SCs (Jessen & Mirsky 2016 the repair Schwann cell; Jessen et al 2016 adaptive cellular reprogramming). As soon as SCs sense axon disruption or lose axon contact (such as upon nerve injury or isolation from the nerve environment), they will transform into repair cells. That repair phenotype is associated with the regain of a proliferative potential and with an upregulation of precursor/immature SC genes and repair associated genes (e.g. c-JUN see Arthur-Farraj et al 2012) as well as downregulation of genes involved in myelination. Thus, as soon as SCs are cultured, the SCs adapt a repair-associated cell state and significantly differ from adult SCs found in intact nerves (but still share the expression of the known SC lineage genes such as S100B, SOX10, …).

Also in cultured nerve fascicles, the SCs within will adapt the repair phenotype after several days (see Weiss et al 2016).

Taken together, the authors report a well-defined SC phenotype of cultured human SCs expressing already reported genes and known to differ from adult SCs found in vivo (Monje P. 2020: Schwann cell cultures; Jessen & Mirsky 2016: the repair Schwann cell; Jessen et al 2016: adaptive cellular reprogramming; Weiss T. et al 2016: the human repair Schwann cell phenotype). However, it would be interesting to which extent the pdSCs show a repair SC-associated expression profile and if it is different to healthy SCs.

Author response: We absolutely agree with the reviewer about the interest of exploring further the phenotype of pdSC and appreciate the clear and detailed information about the SC repair phenotype. We are aware that a repair phenotype is associated with the regain of a proliferative potential and could in theory match the proliferative capacity of our Schwann cells in culture. In particular, the proliferative phenotype described in our article is also present in cSC (as shown in Figure 5 C) and thus seems to characterize cultured human SCs in general, as mentioned by the reviewer. We agree that it would be particularly interesting to study the SC repair phenotype and other pathological ones related to axonal disruption, damage or neuropathy. This manuscript aims to describe only the isolation and culture method of pdSC and the phenotype resulting from the culture. However, we are currently working on a deeper analysis between pdSC and cSCs, as well as among patients, which will be included in a follow-up article. This analysis will further inform on the repair and pathological phenotypes of each patient-derived SC strain.

4. In the methods part, it is written, that the RNA-sequencing analysis was performed with 3 12wells of p3 pdSCs from 6 donors. This does not equal "n=18" as stated in the methods. “n” should indicate the donor number, which is 6, and from each donor, three 12wells of p3 pdSC cultures have been analyzed, this means, pdSCs from one donor have been sequenced in triplicates. In turn, the cSC cultures of one donor (n=1 not 8), have been analyzed in p4, p5 and p6, this means, three replicates of cSCs from one donor have been sequenced. As a consequence, the statistical analysis of the sequencing data could have been performed in a wrong way and should be checked by a bioinformatician.

Author response: The reviewer makes a very fair point noticing the limitations in n number and availability of donors. As mentioned in the previous question, the aim of this manuscript is to prove the identity of SCs isolated with the described method and their phenotype in culture. Thus, the individual differences between each patient-derived cell strain or between passages is not our main interest. Therefore, in our pdSC and cSC data set we have included all biological and technical replicates as part of the total n number in order to have a clearer vision of the common characteristic of all control and patient-derived SCs when cultured with this method. In future studies where we deepen into individual differences, we do cluster technical replicates as suggested by the reviewer.

Other concerns:

a. The authors write several times, that their protocol allows the isolation of proliferative human SC “lines”. A cell line is defined by an unlimited replicative potential but the pdSCs have not been cultured beyond p3.

Author response: The reviewer is absolutely right. We'd like to apologize for the miswording. We have rephrased the word 'lines' or haveremoved it from the text.

b. It is widely accepted amongst Schwann cell biologists that the commercial available human SCs have indeed less in common with fresh isolated SCs from human nerves. The cSC does not even express the SC lineage marker SOX10. Therefore, the label 'human SC control' should be questioned.

Author response: We agree with the reviewer that a primary culture of SCs, as are pdSCs, show more Schwann cell characteristics than commercially available human SCs, as we have proved in this manuscript. Unfortunately, as mentioned in the discussion and in our first answer, we were not able to obtain SCs from healthy donors as controls. Thus we were limited to the use of commercial SCs in order to prove the identity of our cells, which we further confirmed through comparisons with reference data sets. This very good point highlights the importance of our described protocol, which eases and widens their isolation and primary culture, leading the way to personalized medicine, while opening new possibilities for better commercially available SCs.

c. The methods part did not state where the human fibroblast culture was derived/purchased from. Indeed, human fibroblasts are as diverse as the tissue they are derived from and might differ from "nerve fibroblasts" (a collective term for 'no SC' including everything from endothelial cells, to pericytes, …). In addition, fibroblast cultures from at least 3 donors should have been included in the analysis.

Author response: This fibroblast sample was isolated from a skin biopsy of a healthy volunteer and has been previously described in Karl et al, 2019; Kress et al, 2021 and Egenolf et al, 2021 [1-3]. We have included this information in the method section, in page 16, lines 384-389, "*As control of a fibroblast cell type, a sample isolated from a skin biopsy of a healthy volunteer and previously described [78-80] was used. In summary, a 6-mm skin punch biopsy from the calf was cut into small dermal pieces and placed in fibroblast medium (DMEM/F-12 with 10% FCS and 1% penicillin/streptomycin) for 1hr to allow tissue adherence before adding additional medium. Cultures were maintained with medium changes every 2–3 days until fibroblast outgrowth, and cells were expanded and further processed at passage four*", including the three references in the text. We agree with the reviewer that the inclusion of one fibroblast sample may not be enough to perform a full statistical and robust analysis. In this manuscript, we aimed to include this one sample in order to have a clearer vision of the differences between SC and fibroblast cell types, and better identify pdSC.

d. In the last sentence of the abstract, it was written that the generated SC cultures could serve as tools for "therapeutic purpose", which one would that be?

Author response: As mentioned at the end of the discussion, we follow the idea that the isolation of pdSC may be used to investigate the proinflammatory, pro- or demyelinating phenotypes to which Schwann cells may contribute. This may help to improve treatment options for individual patients.

e. Was the immunostaining for SOX10 and S100B performed with all SC cultures of the 6 donors?

Author response: The immunostaining for SOX10, S100B and MBP was initially performed with 2 out of the 6 donors. However, we realized during the revision process that it would be beneficial to include the immunostainings from all donors, including the fibroblast cell line and the commercially

available SC used as control. This can now be found in Figure 2 B and as an additional supplementary figure (Supplementary figure 2). The new data was further explained in the text, in page 4, line 93 to 99, "*The immunofluorescence staining showed consistent expression of SOX10 and S100B in pdSC, with a lower expression in cSC and in a reference sample from a human fibroblast culture (Fibroblast_1) (Fig. 2B). This expression was heterogeneous among pdSC strains isolated from different patients (Suppl. Fig. 2 A). In addition, the MBP marker showed low and unspecific results in all samples (Suppl. Fig. 2 A). Moreover, we compared the expression of Fibronectin 1 (FN1), a marker commonly associated with fibroblasts, among cSC, pdSC and Fibroblast_1 (Suppl. Fig. 2 B). While results showed a similar expression among all samples, differences in morphology between Schwann cells (pdSC) and fibroblasts (Fibroblast_1) can be clearly identified (Fig. 2B)*" and in methods, section "Immunocytochemistry", page 16, line 392 and 399.

f. To prove whether the used antibodies are specifically targeting the desired protein, it is supportive to show a SC culture containing some fibroblasts to illustrate positive and negative SOX10 and S100B staining side by side.

Author response: As mentioned above, we have now included this immunostaining in Figure 2 B and as an additional supplementary figure (Supplementary figure 2).

g. The Immunostaining of MBP is presumably not a specific staining because: 1) the MBP protein is downregulated in cultured human SCs (Weiss et al 2016; Peng et al 2020). Please note that the MBP gene can still be expressed, 2) the MBP staining looks exactly like the S100B staining. A similar staining pattern of different proteins could indicate an unspecific antibody binding and should be investigated.

Author response: The reviewer makes a fair point questioning the specificity of the MBP antibody. The antibody used in this study was a monoclonal rat antibody to MBP #ab7349, Abcam, RRID:AB_305869, which had been used in over 308 publications (*https://www.abcam.com/en-us/products/primary-antibodies/myelin-basic-protein-antibody-12-oligodendrocyte-marker-ab7349?srsltid=AfmBOoppU6GUDtC2U5W_0Bjf44hIP-Iju1NkbbbTDxaHH3fW6s3M1fi7*), thus promising good specificity and results. However, as mentioned above, we repeated and expanded the immunostainings during this revision, including MBP. We agree that the results show a low specificity, possibly due to the low expression in all samples. Thus, we removed it from the main text (Figure 2) and included it only in Supplementary Figure 2.

h. In the discussion (line 276) it was written that a single-cell analysis would be necessary to determine the exact purity of the SC cultures. Actually, to define the purity of SC cultures, immunofluorescence stainings or flow cytometry against established SC markers would be sufficient.

Author response: The reviewer makes a good and fair suggestion, that would maybe help clarify the purity of the culture. However, our study has also proved that pdSC shows a fibroblast-like phenotype and can share proliferative markers with other cell types, as well as lower expression of Schwann cell markers depending on the strain. Thus, although immunofluorescence staining and flow cytometry may help differentiate cell types with clear and different phenotypes, a complete single-cell transcriptomic would be necessary to fully understand the different cell types and phenotypes they present. In addition, since the SC cultures are of multicellular origin, the advantage of this strategy is that it better reflects the natural variability of Schwann cell phenotypes. In contrast, selecting specific

Schwann cell line clones would likely limit the experimental and clinical potential of the cultures by reducing phenotypic diversity.

i. In the discussion (line 290) pdSCs were proposed as “tool to study the pathophysiology of individual neuropathies, leading the way to personalized medicine”. This statement is exaggerated as distinct PNP associated SC phenotypes / expression profiles / functional differences have not been identified and the repair phenotype, that SCs gain after culture, will also influence such a potential PNP phenotype.

Author response: The reviewer is absolutely right pointing out that the phenotype gained in culture may influence and mask the potential PNP phenotype. Nevertheless, the genetic study of each cell strain and their behavior upon different stimuli, as well as in different culture conditions or passages, can elucidate the PNP-related characteristics of that specific patient. We are at the moment working on analyzing the individual differences among cell strains and their correlations with the neuropathic symptoms seen in each patient. Furthermore, in the near future, we see the potential of adding pdSC to induced human sensory neurons, which are produced in our lab as well, and study their interaction and relationship.

The authors have generated precious RNA-sequencing data of PNP patient derived SC cultures. It would be interesting to compare them to the already existing RNA sequencing data of human SC cultures and try to retrieve information about the differences between dysfunctional PNP-associated SCs and control SCs. Based on that information, functional differences might be identified that could be validated in pdSC cultures. Such findings would be of high interest for the SC community.

Author response: We would like to thank you for the very helpful contribution that your comments have provided to our manuscript. In this article, we have already compared our results with existing RNA sequencing data of human SC (Human Protein Atlas[Ref 34]; Karlsson et al, 2021[Ref 35]; Peng et al, 2020[Ref 36]; Gerber et al; 2021[Ref 37] and Zhang et al, 2021[Ref 38]) in figures 3, 4 and 5. In addition, we also hope that our future studies on individualized PNP patient derived SCs and their PNP-associated phenotypes will indeed be of great interest for the SC community.

Reviewer #2 (Remarks to the Author):

The manuscript by García-Fernández et al. presents a methodology for isolating Schwann cells (SC) from small human sural nerve biopsy samples and a comprehensive transcriptome analysis to characterize their phenotype. This work represents a contribution to the field, particularly for studying patient-derived SC biology, which is critical for understanding PNS disorders. The study combines good experimental design with an in-depth analysis.

Author response: Thank you!

Suggestions for Additional Analysis:

qPCR Variability (Figure 2C): The relative expression of SC markers varies between the six patient-derived SCs. Could the authors comment on possible correlations between expression levels and patient characteristics such as age, disease duration, or diagnosis? Moreover, is there a correlation

between the expression levels of the three markers (SOX10, S100B, and MBP) within the same patient samples? Differentiating the dots in the graph and/or providing a supplementary table with the raw data would help.

Author response: The reviewer makes an important point. We have checked whether the expression of Schwann cell markers such as SOX10, S100B and MBP correlates with each other. We have found that S100B and MBP do correlate strongly ($p < 0.0001$), but not SOX10. Please see below a heatmap of the TPM of each gene and the correlation matrix after performing a Pearson correlation test:

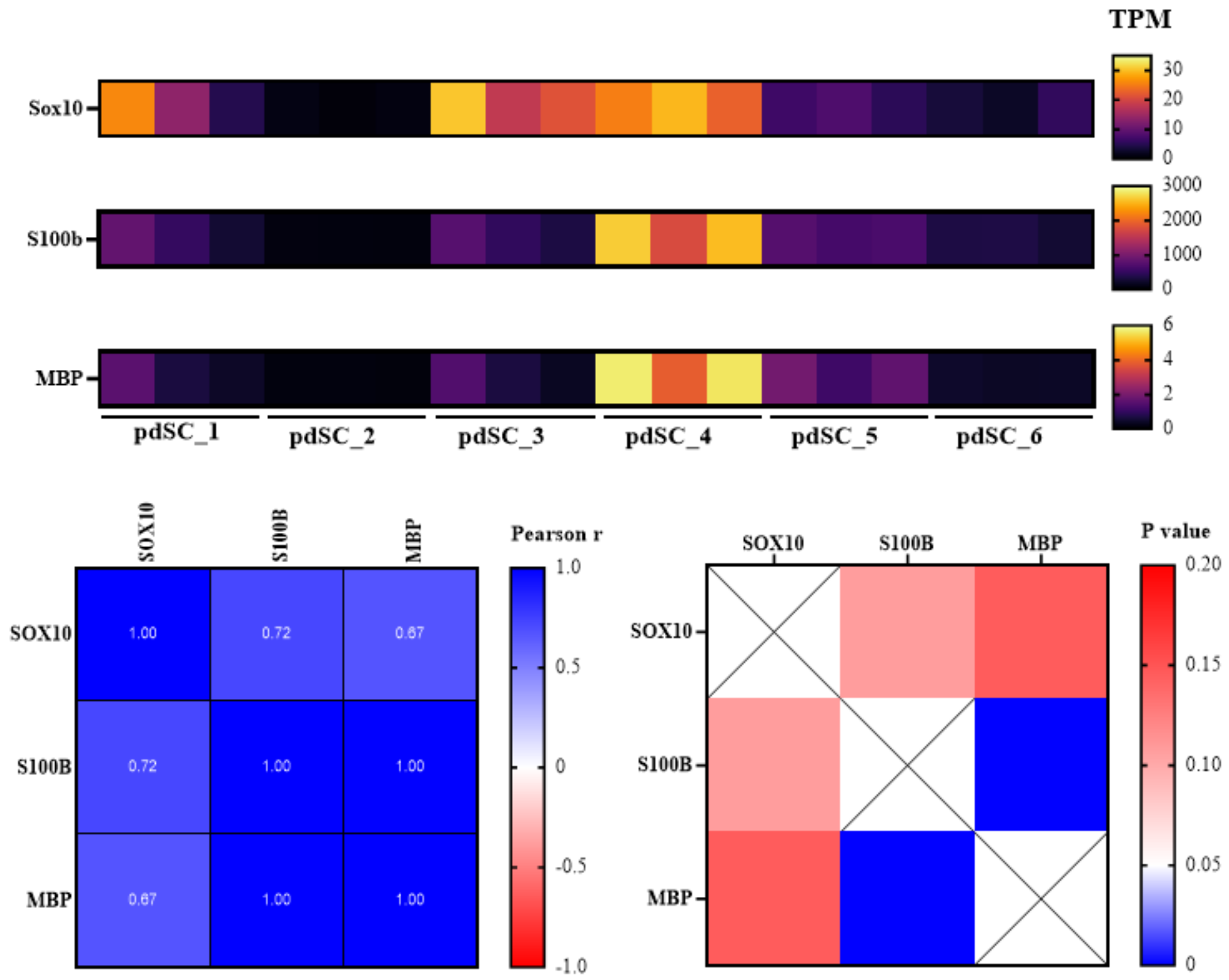

In addition, the raw human bulk RNA-seq data generated in this study is available at the European Genome-phenome Archive (EGA) under accession number EGAS50000000737 and the TPM tables have also been provided as supplementary.

Moreover, in this study, we have focused on the general identification and characterization of patient-derived Schwann cells isolated with this method, rather than the differences among cells obtained from different patients, and thus haven't included this particular information. However, we agree that this is of great interest and are working towards studying this in depth and will be presented in a follow-up article.

Purity Analysis: While the study clearly confirms the SC phenotype through transcriptome analysis, including a quantification of SOX10-negative nuclei in the cultures would strengthen the purity assessment. Additionally, staining for a fibroblast-specific marker could provide more insight into potential contamination levels.

Author response: The reviewer makes an excellent point. We have included a supplementary figure (supplementary figure 2) showing immunofluorescence stainings of SOX10, S100B and MBP among all

patient-derived Schwann cell strains, cSC and Fibroblast_1, as well as Fibronectin (FN1) as a commonly understood fibroblast-specific marker. We have also included a further explanation of this figure in the results section, in page 4, line 91 to 99, “*The immunofluorescence stainings showed consistent expression of SOX10 and S100B in pdSC, with a lower expression in cSC and in a reference sample from a human fibroblast culture (Fibroblast_1) (Fig. 2B). This expression was heterogeneous among pdSC strains isolated from different patients (Suppl. Fig. 2 A). In addition, the MBP marker showed low and unspecific results in all samples (Suppl. Fig. 2 A). Moreover, we compared the expression of Fibronectin 1 (FN1), a marker commonly associated with fibroblasts, among cSC, pdSC and Fibroblast_1 (Suppl. Fig. 2 B). While results showed a similar expression among all samples, differences in morphology between Schwann cells (pdSC) and fibroblasts (Fibroblast_1) can be clearly identified (Fig. 2B)”* and in methods, section “Immunocytochemistry”, page 16, line 392 and 399. We also understand and appreciate the suggestion of quantifying SOX10-negative nuclei as an additional purity assessment. However, it is not clear yet how to discriminate low abundance expression of Sox10 from high expression to correctly discriminate SCs. We believe this can only be accurately done with scRNA seq and clustering, which is out of scope at this stage of the study.

Supplementary Figure 2: This figure includes 18 samples which from the methods they represent triplicates at P3, please indicate in the graph which are triplicates (do they cluster together in the PCA plot?) and/or include in a supplementary table the PCA values for triplicates. This would help in understanding the reproducibility of the protocol across replicates.

Author response: The reviewer is right, pointing out that the 18 samples included in this figure represent triplicates from each pdSC strain and we agree that checking their clustering would be of great interest. However, as mentioned above, the aim of this manuscript is to prove the identity of SCs isolated with the described method rather than studying the differences between each patient-derived cell strain. Thus, in this figure and in our pdSC and cSC data set, we had included all biological and technical replicates as part of each data group in order to have a clearer vision of the common characteristic of all control and patient-derived SCs when cultured with this method. However, we have added the labels of each sample to the PCA graph and to the distance heat map (Suppl. Fig. 3 B, C) for more transparency. We hope this will help identify the clustering and distance between each technical and biological replicate, as well as between the different groups.

References

1. Karl, F., et al., *Patient-derived in vitro skin models for investigation of small fiber pathology.* Ann Clin Transl Neurol, 2019. **6**(9): p. 1797-1806.
2. Kress, L., et al., *Differential impact of keratinocytes and fibroblasts on nociceptor degeneration and sensitization in small fiber neuropathy.* Pain, 2021. **162**(4): p. 1262-1272.
3. Egenolf, N., et al., *Diagnosing small fiber neuropathy in clinical practice: a deep phenotyping study.* Ther Adv Neurol Disord, 2021. **14**: p. 17562864211004318.

Response to Reviewers

Manuscript COMMSBIO-24-7498-T

Dear Dr. Bu,
Thank you for considering our manuscript, *"Isolation, culturing, and gene expression profiling of Schwann cells from human sural nerve,"* and for giving us the opportunity to revise and resubmit an improved version. We sincerely appreciate the time and effort that you and the reviewers have invested in evaluating our work. The reviewers' insightful comments have been extremely helpful in strengthening the manuscript.

We have addressed most of the suggestions provided. Changes introduced in this second revision are highlighted in blue, while modifications from the first revision remain visible in yellow. Below, please find our point-by-point responses to the reviewers' comments and concerns, with new responses presented in blue.

In addition, we have updated the figures by converting the graphs to boxplots with individual data points to better illustrate the data distribution. We have also provided the numerical source data not only as supplementary PDF tables, as in the previous version, but now also as an Excel file ("Supplementary data_1"). This is noted in the manuscript under "Data availability" (lines 699–700): "All numeric data used in Figures 2, 3, 4, and 5 are included as supplementary tables provided with this article and as an additional Excel file." We have made every effort to comply with the Nature Portfolio's data deposition requirements and the policies for research involving human and animal subjects; if we have unintentionally overlooked any aspect of these policies, we would be grateful to be informed and are happy to make any necessary changes.

Reviewers' comments:

Reviewer #1 (Remarks to the Author):

The authors' efforts to revise the manuscript according to the reviewers' suggestions are appreciated. The idea and potential significance of this work are acknowledged and valued. Nevertheless, in its current form, the experimental evidence does not support the main conclusions drawn by the authors.

The authors write: "The data show that the culture of human SC obtained with this method presents a good preservation of SC phenotypes and is highly reproducible when applied to small biopsy samples from different patients." Unfortunately, the presented data do not support this statement. Immunofluorescence images show stainings for S100 and SOX10, that identified SCs in the pdSC cultures, but they also show that pdSC cultures contained different amounts S100/SOX10 negative cells (Fig.2B, S.Fig.2A). These SOX10 negative cells are not SCs, most likely, they represent fibroblasts. Bulk RNA sequencing performed on these cultures will inevitably yield a mixed gene expression profile, reflecting both SC and fibroblast populations. SC-specific transcripts will still be detected; however, their relative abundance decreases proportionally with the proportion of fibroblasts in the culture. Accordingly, the RNA sequencing data show that triplicates from one donor were negative for SOX10, and other SC markers were barely detected, whereas triplicates from another donor exhibited strong SOX10 expression, which correlated with the presence of additional SC-associated genes (see

triplicates in Fig. 3 and Fig. 4). Indeed, fibroblast-associated genes were also present in the pdSC cultures, as shown in Fig. 4 and Fig. 5. Nevertheless, the authors concluded that "… the expression of fibroblast markers seemed to be prominent in pdSC, indicating a fibroblast-like phenotype." while never considering that this signature is just a results of a contaminating fibroblast population. Even though their own immunofluorescence stainings and analyses show that pdSC cultures are not pure, the authors write that "In addition, the homogeneity of the culture observed in our immunostaining analyses suggested the elimination of other contaminating cells, such as fibroblasts, in the process."

A more thorough evaluation of the results and a more cautious interpretation are necessary to ensure that the conclusions accurately reflect the presented data.

Author response: Thank you for giving us this very important feedback. We understand the concern regarding the purity of the culture. As mentioned by the reviewer, cultures from some of the donors did show a homogeneous staining and high expression of SC markers such as SOX10 and S100B. All cultures were processed following the same protocol and cultured in the same medium, as well as the commercial SC used as control, thus suggesting that the differences we see are based on the origin of the biopsy and not the protocol itself. In addition, the cSC showed as well low expression of SOX10 in both the stainings and the RNAseq, but has been commercially proven and used in over 26 publications since 2005 to study Schwann cells characteristics and behavior. Furthermore, in Figure 2 B, the reader can see the clear difference, not only in expression but also in morphology, between a fibroblast culture and a pdSC culture. We thus believe that our cultures contain Schwann cells showing different stages and phenotypes depending on the origin of the cells, which lead to different expression of SC markers. However, we agree with the reviewer that then the sentence "*The data show that the culture of human SC obtained with this method presents a good preservation of SC phenotypes and is highly reproducible when applied to small biopsy samples from different patients*." wouldn't be entirely correct and have thus removed it from the text.

We also understand that despite our efforts and clear differences, there is the possibility of fibroblasts contaminating our cultures, which is why we had continued the sentence "*In addition, the homogeneity of the culture observed in our immunostaining analyses suggested the elimination of other contaminating cells, such as fibroblasts, in the process.*" with "*However, single-cell analyses would be necessary to determine the exact purity of the SC culture and verify the absence of other cell types*". We agree that due to the differences in SC phenotype and stages between donors, the cultures are not all homogeneous and have thus rephrased those statements with "*Moreover, the presence of different SC phenotype among cultures hinders the identification of contaminating cells. Thus, future work incorporating single-cell transcriptomic analyses will be essential to dissect the cellular composition in greater detail, including distinct Schwann cell subtypes and transitional states between clusters.*" We hope these changes towards a more humble conclusion will meet the expectations.

Reviewers' Comments to the Authors:

Reviewer #1:
In this manuscript, the authors show that they are able to culture human Schwann Cells (SCs) from about 4 mm sural nerve biopsies and performed bulk RNA sequencing analyses to confirm SC identity and describe their phenotype.
There is enthusiasm for the presented possibility to culture SCs from such small biopsies as it allows to compare the supposed dysfunctional SCs derived from PNP patients with those from healthy controls to reveal potential differences and affected pathways. Unfortunately, such an analysis was not performed but the manuscript only focused on characterizing the pdSC cultures based on their bulk sequencing data.

Author response: Thank you for appreciating the importance of our study and identifying one of its main limitations. Nerve biopsies are obtained through an invasive procedure that is only performed in patients for diagnostic purposes. This unfortunately means that we were not able to obtain SCs from healthy donors as controls and thus perform the analysis suggested by the reviewer. Commercially available SCs were used instead as the best available control. This limitation was expanded in the discussion in page 13, line 297-300, "In addition, due to the invasive nature of nerve biopsy, obtaining nerve samples from healthy volunteers was not ethically permissible. Under other circumstances, the isolation of Schwann cells from healthy donors could help clarify the contribution of neuropathy to the characteristics of each cell strain.".

Reviewer Response: There seems to have been a misunderstanding. It was suggested to compare the pdSC samples with publicly available RNA sequencing data from healthy human Schwann cells, not to obtain new biopsies from healthy donors for additional analysis.
When using commercially available "SCs", their SC identity has to be confirmed before using it as a control. By looking at the stainings and RNA sequencing data, the cSCs are negative for established SC markers. Thus, they cannot serve as a proper SC control.

Author response: Thank you for clarifying your recommendation. We understand that your intention was to encourage comparison of our pdSC transcriptomes with publicly available datasets from primary literature sources, not to suggest additional biopsies from healthy donors. We agree that such benchmarking represents a valuable approach.

In line with this, in the current manuscript we included comparisons with publicly available RNA-seq datasets of human Schwann cells, including (1) the Human Protein Atlas and (2) the dataset from Peng et al., 2020 (Scientific Reports 10:18433). These datasets were selected because they provide accessible curated datasets of human Schwann cell transcriptomes.

We appreciate that there are additional datasets in the literature that could, in principle, be integrated to extend these comparisons. Unfortunately, incorporating further datasets is not feasible within the current revision due to time and dataset processing constraints. To avoid implying that the scope of our comparison is comprehensive, we have revised the manuscript to make explicit that the datasets we used represent the best available and accessible resources for our aims, but not an exhaustive survey of all published Schwann cell datasets. This clarification appears in the revised Discussion ("*In addition, while additional primary literature datasets exist that could, in principle, be integrated into broader comparative analyses, these were beyond the feasible scope of the present study. Our aim was limited to confirming Schwann cell identity using accessible, curated datasets;*

therefore, the comparisons presented here should be interpreted as representative, rather than comprehensive.“ page 13, lines 288-292).

With regard to the commercially available SCs (cSCs), these cells were used as an additional reference because they have been characterized by the provider (S100β-positive, pathogen-free) and widely used in the literature for Schwann cell studies (>26 publications since 2005). However, we agree with the reviewer that these cells are not an ideal control for transcriptomic comparison in our context, thus our previous answer acknowledging and expanding this limitation in the text.

There are serious concerns about the study that are listed below.

1. The study is presented as novel protocol to isolate human SCs from small biopsies, but actually uses well established tissue digests and culture conditions. Therefore, it rather represents an adaption of existing protocols (e.g. Weiss et al 2018; Peng et al 2020) for the culture of human SCs but from small biopsies.

Author response: We absolutely agree with giving previous protocols the acknowledgement that they deserve for their contribution in inspiring this method. However, our protocol is not a direct modification of one or several of them, in particular not Weiss et al 2018 or Pent et al 2020, since we do not use any of the steps followed in those protocols, such as magnetic separation, FACS or two-step enrichment exploiting differential adhesion properties. Nevertheless, we have indeed adapted the D-valine media and protocol used in Kaewkhaw, R., A.M. Scutt, and J.W. Haycock, Integrated culture and purification of rat 510 Schwann cells from freshly isolated adult tissue. Nat Protoc, 2012.7(11): p. 1996-2004 to obtain SCs from smaller human material than had ever been tested before. We believe that this concept is well explained in the main text, particularly in page 2, lines 41 to 57.

Reviewer Response: There seems to have been a misunderstanding. The similarity with published protocols is the used dissociation solution for fascicle tissue digest and culture conditions, meaning the culture medium supplemented with FGF-basic and PDGF. It is correct that the D-valine medium was so far only described for rodent SC culture. Nevertheless, the presented approach appears to be an adaptation of several previously established SC isolation methods, rather than a novel protocol.

Author response: We thank the reviewer for pointing out the misunderstanding. Our intention is not to claim that the enzymatic dissociation or general culture supplements used here are entirely new—indeed, we acknowledge that our dissociation solution is based on Weiss et al. (2018), and that culture conditions such as bFGF and PDGF supplementation are widely used across several previously published Schwann cell protocols. As correctly noted by the reviewer, the present protocol was developed by systematically testing and combining elements from multiple established approaches. The resulting workflow therefore represents an adaptation and integration of previously described methods, optimized specifically to enable reliable isolation and expansion of Schwann cells from very small (~4 mm) diagnostic human sural nerve biopsies, which to our knowledge had not been previously demonstrated. To make this clearer to the reader, we have revised the sentence in the introduction (line 57) as follows: “*In this study, we describe a protocol that incorporates key elements of previously published culture conditions while enabling the successful use of D-valine medium to isolate and expand a pure Schwann cell culture from ~4 mm diagnostic human sural nerve biopsies.*”

2. There are key studies already characterizing cultured human SCs using proteome and transcriptome analyses (Weiss et al 2016; Peng et al 2020). The first one was not cited in this manuscript, although the RNA-sequencing analysis was performed in a similar way, including primary SCs and fibroblast as well as nerve fascicle samples and resulting phenotypes were described and also functionally validated. A comparison of the human SC expression profiles generated by this and the other studies would be very interesting to identify possible PNP associated differences in the isolated pdSCs.

Author response: Thank you for bringing this study to our attention, we have included a citation of this article in the discussion, page 11, line 217 (reference 43). We absolutely agree that a comparison between our analysis and other published ones would be of great interest. Unfortunately, due to the size limitations of this article, we had to focus on the studies that we considered most relevant. The RNA-sequencing analysis performed in Weiss et al 2016 focuses on markers related to repair phenotype, debris clearance, phagocytosis, antigen presentation or T-cell related signaling. While interesting, our manuscript focuses on Schwann cell identity, and we will use future studies to focus on other signaling pathways or pathogenic phenotypes. Since the RNA-sequencing data is available, we also invite other authors to perform whichever comparisons may be more relevant or interesting for them.

3. The authors write in results line 173: “pdSCs are proliferating primary cells that can change their phenotype in culture and diverge from commonly understood SC found in vivo” or discussion line 234: “these results suggest that pdSC may be in a proliferative state rather than a myelinating state, and therefore lack the capacity to myelinate axons.”, which raises the question whether they are familiar with the repair phenotype of SCs (Jessen & Mirsky 2016 the repair Schwann cell; Jessen et al 2016 adaptive cellular reprogramming). As soon as SCs sense axon disruption or lose axon contact (such as upon nerve injury or isolation from the nerve environment), they will transform into repair cells. That repair phenotype is associated with the regain of a proliferative potential and with an upregulation of precursor/immature SC genes and repair associated genes (e.g. c-JUN see Arthur-Farraj et al 2012) as well as downregulation of genes involved in myelination. Thus, as soon as SCs are cultured, the SCs adapt a repair-associated cell state and significantly differ from adult SCs found in intact nerves (but still share the expression of the known SC lineage genes such as S100B, SOX10, …). Also in cultured nerve fascicles, the SCs within will adapt the repair phenotype after several days (see Weiss et al 2016). Taken together, the authors report a well-defined SC phenotype of cultured human SCs expressing already reported genes and known to differ from adult SCs found in vivo (Monje P. 2020: Schwann cell cultures; Jessen & Mirsky 2016: the repair Schwann cell; Jessen et al 2016: adaptive cellular reprogramming; Weiss T. et al 2016: the human repair Schwann cell phenotype). However, it would be interesting to which extent the pdSCs show a repair SC-associated expression profile and if it is different to healthy SCs.

Author response: We absolutely agree with the reviewer about the interest of exploring further the phenotype of pdSC and appreciate the clear and detailed information about the SC repair phenotype. We are aware that a repair phenotype is associated with the regain of a proliferative potential and could in theory match the proliferative capacity of our Schwann cells in culture. In particular, the proliferative phenotype described in our article is also present in cSC (as shown in Figure 5 C) and thus seems to characterize cultured human SCs in general, as mentioned by the reviewer. We agree that it would be particularly interesting to study the SC repair phenotype and other pathological ones related to axonal disruption, damage or neuropathy. This manuscript aims to describe only the

isolation and culture method of pdSC and the phenotype resulting from the culture. However, we are currently working on a deeper analysis between pdSC and cSCs, as well as among patients, which will be included in a follow-up article. This analysis will further inform on the repair and pathological phenotypes of each patient-derived SC strain.

Reviewer response: Please note that it is published and well known that SCs enter a proliferative state and downregulate myelin-related genes when cultured. The sentence in line 245: "These results, along with our analyses of the different Schwann cell phenotypes and the observed proliferation in culture, suggest that pdSC may be in a proliferative state rather than in a myelinating state, and therefore lack the capacity to myelinate axons." implies that this observation is novel, although it reflects a well-established phenomenon of cultured SCs.
It is true that the commercial "SCs" used as control also proliferated in culture. However, so would most other cultured human cell types.

Author response: We thank the reviewer for this helpful comment. We fully agree that Schwann cells are well known to dedifferentiate and enter a proliferative, repair-like state when removed from axonal contact or placed in standard culture conditions, and that this transition is accompanied by downregulation of pro-myelin transcription factors and myelin genes. This phenomenon has been described extensively both in vivo after nerve injury and in vitro. Our intention was not to imply that this is a novel observation, and we have added another sentence to explicitly acknowledge that the phenotype we observe in pdSCs is consistent with this well-established Schwann-cell dedifferentiation response under culture conditions: "*This interpretation is consistent with the well-established observation that Schwann cells rapidly dedifferentiate, downregulate myelin-related genes and enter a proliferative repair-like state when cultured or deprived of axonal contact [51-55]*"

4. In the methods part, it is written, that the RNA-sequencing analysis was performed with 3 12wells of p3 pdSCs from 6 donors. This does not equal "n=18" as stated in the methods. "n" should indicate the donor number, which is 6, and from each donor, three 12wells of p3 pdSC cultures have been analyzed, this means, pdSCs from one donor have been sequenced in triplicates. In turn, the cSC cultures of one donor (n=1 not 8), have been analyzed in p4, p5 and p6, this means, three replicates of cSCs from one donor have been sequenced. As a consequence, the statistical analysis of the sequencing data could have been performed in a wrong way and should be checked by a bioinformatician.

Author response: The reviewer makes a very fair point noticing the limitations in n number and availability of donors. As mentioned in the previous question, the aim of this manuscript is to prove the identity of SCs isolated with the described method and their phenotype in culture. Thus, the individual differences between each patient-derived cell strain or between passages is not our main interest. Therefore, in our pdSC and cSC data set we have included all biological and technical replicates as part of the total n number in order to have a clearer vision of the common characteristic of all control and patient-derived SCs when cultured with this method. In future studies where we deepen into individual differences, we do cluster technical replicates as suggested by the reviewer.
Reviewer response: There seems to have been a misunderstanding. The presented n number of 6 is not a limitation of this study. However, defining triplicates from one donor as n = 3 is methodologically incorrect, as these do not represent independent biological replicates. This should be clarified in the methods section and in the respective figure legend to avoid misinterpretation of sample numbers and statistical validity.

Other concerns:

a. The authors write several times, that their protocol allows the isolation of proliferative human SC "lines". A cell line is defined by an unlimited replicative potential but the pdSCs have not been cultured beyond p3.

Author response: The reviewer is absolutely right. We'd like to apologize for the miswording. We have rephrased the word 'lines' or have removed it from the text.

b. It is widely accepted amongst Schwann cell biologists that the commercial available human SCs have indeed less in common with fresh isolated SCs from human nerves. The cSC does not even express the SC lineage marker SOX10. Therefore, the label 'human SC control' should be questioned.

Author response: We agree with the reviewer that a primary culture of SCs, as are pdSCs, show more Schwann cell characteristics than commercially available human SCs, as we have proved in this manuscript. Unfortunately, as mentioned in the discussion and in our first answer, we were not able to obtain SCs from healthy donors as controls. Thus we were limited to the use of commercial SCs in order to prove the identity of our cells, which we further confirmed through comparisons with reference data sets. This very good point highlights the importance of our described protocol, which eases and widens their isolation and primary culture, leading the way to personalized medicine, while opening new possibilities for better commercially available SCs.

Reviewer response: In order to prove the identity of pdSCs, the authors are not forced to use commercial SCs (with limited to non-existing SC characteristics), but should compare their pdSC with available data sets of characterized human SCs.

Author response: Thank you for this clarification. We fully agree that commercially available human SCs often show limited resemblance to freshly isolated primary human Schwann cells and that their use as "controls" is therefore problematic. Our intention was not to present the commercial cells as a gold-standard reference, but rather as an additional comparison point. To properly confirm pdSC identity, our main comparisons were instead made against well-characterized primary human Schwann cell datasets, including those from the Human Protein Atlas and Peng et al., 2020, as shown in Figures 3 and 4. These datasets provide a far more biologically appropriate benchmark for Schwann cell lineage markers such as SOX10, and we relied primarily on these resources—not the commercial line—to establish pdSC identity.

c. The methods part did not state where the human fibroblast culture was derived/purchased from. Indeed, human fibroblasts are as diverse as the tissue they are derived from and might differ from "nerve fibroblasts" (a collective term for 'no SC' including everything from endothelial cells, to pericytes, …). In addition, fibroblast cultures from at least 3 donors should have been included in the analysis.

Author response: This fibroblast sample was isolated from a skin biopsy of a healthy volunteer and has been previously described in Karl et al, 2019; Kress et al, 2021 and Egenolf et al, 2021 [1-3]. We have included this information in the method section, in page 16, lines 384-389, "As control of a fibroblast cell type, a sample isolated from a skin biopsy of a healthy volunteer and previously described [78-80] was used. In summary, a 6-mm skin punch biopsy from the calf was cut into small dermal pieces and placed in fibroblast medium (DMEM/F-12 with 10% FCS and 1% penicillin/streptomycin) for 1hr to allow tissue adherence before adding additional medium. Cultures were maintained with medium changes every 2–3 days until fibroblast outgrowth, and cells were expanded and further processed at passage four", including the three references in the text. We agree with the reviewer that the inclusion of one fibroblast sample may not be enough to perform a full statistical and robust analysis. In this manuscript, we aimed to include this one sample in order to have a clearer vision of the differences between SC and fibroblast cell types, and better identify pdSC.

d. In the last sentence of the abstract, it was written that the generated SC cultures could serve as tools for "therapeutic purpose", which one would that be?

Author response: As mentioned at the end of the discussion, we follow the idea that the isolation of pdSC may be used to investigate the proinflammatory, pro- or demyelinating phenotypes to which Schwann cells may contribute. This may help to improve treatment options for individual patients.

Reviewer response: This should be clarified in the manuscript because the sentence "…serve as tools for diagnostic and therapeutic purposes" could be misunderstood in that way, that the intention is to use the pdSCs as therapy.

Author response: Thank you for pointing out this potential misunderstanding. Our intention was not to imply that pdSCs themselves would be used as a therapeutic product, but rather that they could be used as an experimental tool to study disease-related Schwann cell phenotypes that may ultimately inform therapeutic decision-making. To avoid any ambiguity, we have revised the abstract to read: "*...serve as tools for diagnostic purposes*" removing the reference to therapeutic use.

e. Was the immunostaining for SOX10 and S100B performed with all SC cultures of the 6 donors?

Author response: The immunostaining for SOX10, S100B and MBP was initially performed with 2 out of the 6 donors. However, we realized during the revision process that it would be beneficial to include the immunostainings from all donors, including the fibroblast cell line and the commercially available SC used as control. This can now be found in Figure 2 B and as an additional supplementary figure (Supplementary figure 2). The new data was further explained in the text, in page 4, line 93 to 99, "The immunofluorescence staining showed consistent expression of SOX10 and S100B in pdSC, with a lower expression in cSC and in a reference sample from a human fibroblast culture (Fibroblast_1) (Fig. 2B). This expression was heterogeneous among pdSC strains isolated from different patients (Suppl. Fig. 2 A). In addition, the MBP marker showed low and unspecific results in all samples (Suppl. Fig. 2 A). Moreover, we compared the expression of Fibronectin 1 (FN1), a marker commonly associated with fibroblasts, among cSC, pdSC and Fibroblast_1 (Suppl. Fig. 2 B). While results showed a similar expression among all samples, differences in morphology between Schwann cells (pdSC) and fibroblasts (Fibroblast_1) can be clearly identified (Fig. 2B)" and in methods, section "Immunocytochemistry", page 16, line 392 and 399.

Reviewer response: The additional stainings are appreciated. They show that pdSC cultures contain a significant amount of cells that are negative for established SC markers SOX10 and S100B. The most obvious conclusion would be, that the cultures contain different amounts of fibroblasts but the authors never commented about the fibroblast contamination in their pdSC cultures.
The FN1 staining shows that FN1 is stronger expressed in pdSCs than in the fibroblasts. Why did the authors not use Thy.1 as a fibroblast marker?

Author response: We thank the reviewer for these comments. As mentioned in previous answers, while some pdSCs appear negative for classical SC markers, we believe these cells represent Schwann cells in alternative phenotypic states rather than contaminating fibroblasts. All cultures were processed identically, including cSCs, which also show low SOX10 expression but have been validated as Schwann cells and used extensively in the literature. Overall, our data indicate that the cultures contain Schwann cells with donor-dependent phenotypic variability rather than fibroblast contamination.

At the time of the original submission, the staining panel for fibroblasts was limited, and FN1 was the only validated antibody available to us. Although Thy1 is frequently used as a fibroblast marker, its expression in glial cells and neurons reduces its specificity. During the revision period, we followed the reviewer's suggestion and evaluated Thy1 expression both at the transcriptomic level (RNA-seq; panel A) and by immunostaining (panel B). A higher-magnification view comparing the morphology of Schwann cells (pdSC) and fibroblasts (Fibroblast_1) is also included. These analyses demonstrate that while Thy1 expression varies among pdSC and cSC cultures and lacks specificity as a single marker—similar to FN1—it nevertheless highlights clear morphological differences between Schwann cells and fibroblasts.

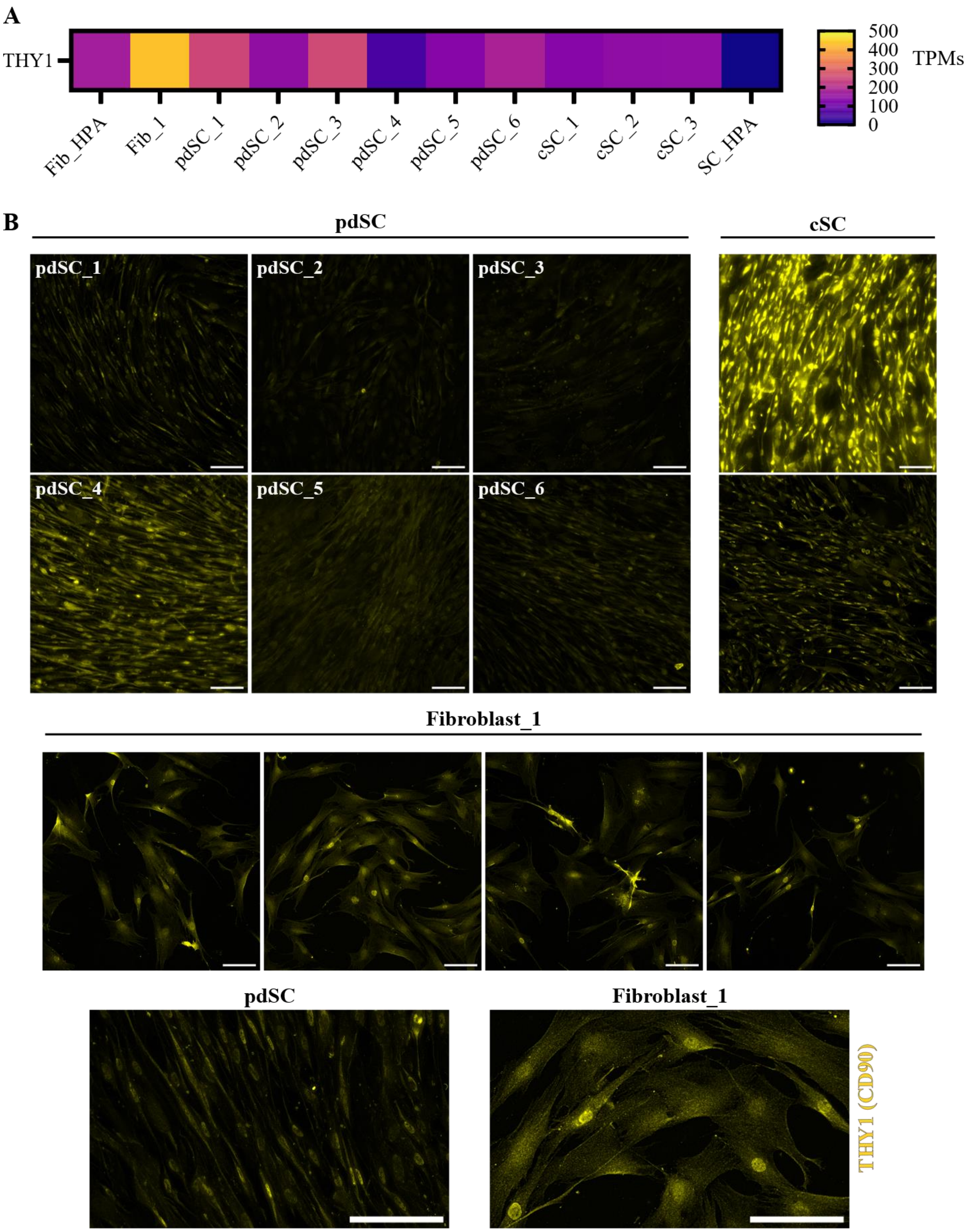

Figure. A) THY1 expression from the RNA-sequencing dataset presented in this study, among all samples including Fib_HPA and SC_HPA from the Human Protein Atlas (HPA), in transcripts per million (TPMs). **B**) THY1 (yellow) IF staining performed in all samples included in the study. cSC, control human Schwann cell; pdSC, patient-derived Schwann cell. Bar:100µm.

f. To prove whether the used antibodies are specifically targeting the desired protein, it is supportive to show a SC culture containing some fibroblasts to illustrate positive and negative SOX10 and S100B staining side by side.

Author response: As mentioned above, we have now included this immunostaining in Figure 2 B and as an additional supplementary figure (Supplementary figure 2).

g. The Immunostaining of MBP is presumably not a specific staining because: 1) the MBP protein is downregulated in cultured human SCs (Weiss et al 2016; Peng et al 2020). Please note that the MBP gene can still be expressed, 2) the MBP staining looks exactly like the S100B staining. A similar staining pattern of different proteins could indicate an unspecific antibody binding and should be investigated.

Author response: The reviewer makes a fair point questioning the specificity of the MBP antibody. The antibody used in this study was a monoclonal rat antibody to MBP #ab7349, Abcam, RRID:AB_305869, which had been used in over 308 publications (https://www.abcam.com/en-us/products/primary-antibodies/myelin-basic-protein-antibody-12-oligodendrocyte-marker-ab7349?srsltid=AfmBOoppU6GUDtC2U5W_0Bjf44hIP-Iju1NkbbbTDxaHH3fW6s3M1fi7), thus promising good specificity and results. However, as mentioned above, we repeated and expanded the immunostainings during this revision, including MBP. We agree that the results show a low specificity, possibly due to the low expression in all samples. Thus, we removed it from the main text (Figure 2) and included it only in Supplementary Figure 2.

h. In the discussion (line 276) it was written that a single-cell analysis would be necessary to determine the exact purity of the SC cultures. Actually, to define the purity of SC cultures, immunofluorescence stainings or flow cytometry against established SC markers would be sufficient.

Author response: The reviewer makes a good and fair suggestion, that would maybe help clarify the purity of the culture. However, our study has also proved that pdSC shows a fibroblast-like phenotype and can share proliferative markers with other cell types, as well as lower expression of Schwann cell markers depending on the strain. Thus, although immunofluorescence staining and flow cytometry may help differentiate cell types with clear and different phenotypes, a complete single-cell transcriptomic would be necessary to fully understand the different cell types and phenotypes they present. In addition, since the SC cultures are of multicellular origin, the advantage of this strategy is that it better reflects the natural variability of Schwann cell phenotypes. In contrast, selecting specific Schwann cell line clones would likely limit the experimental and clinical potential of the cultures by reducing phenotypic diversity.

Reviewer response: If the purity of pdSC cultures is not clarified, then the authors don't know if the 'fibroblast-like' phenotype they describe for their pdSC cultures is just a results of a contaminating fibroblast population.
In addition, propagating single SC "clones" will face the challenge of the limited live span of cultured human cells and the acquisition of a senescent phenotype, thus, multiple passaged human SCs will likely diverge from their original phenotype and not be useful for functional studies. How will the authors address that?

Author response: We understand the reviewer's concern that a fibroblast-like phenotype could theoretically arise from a contaminating fibroblast subset. As mentioned above, several donor-

derived cultures showed homogeneous staining and high expression of SC markers, indicating that our protocol can yield highly pure Schwann cell cultures and that the variability observed is donor-dependent rather than methodological. In addition, as shown in Figure 2B, there is a clear difference in both marker expression and cell morphology between fibroblast cultures and pdSC cultures. We therefore believe that our preparations contain Schwann cells exhibiting different phenotypic states, while acknowledging that we cannot confirm 100% purity. For this reason, we explicitly included this limitation in the discussion (line 288): "*In addition, the presence of different SC phenotypes among cultures difficulties the identification of contaminating cells. Thus, future work incorporating single-cell transcriptomic analyses will be essential to dissect the cellular composition in greater detail, including distinct Schwann cell subtypes and transitional states between clusters.*"

Regarding the reviewer's point about propagating single SC clones, we fully agree that this approach is limited by the finite lifespan and senescence of cultured human cells. To mitigate this, we cryopreserved pdSC at passage 2 immediately after isolation (Methods, lines 365–373) and used them at early passage (P3) after thawing. Although we have not yet observed reduced proliferation at later passages, the onset of senescence in human Schwann cells is not fully defined. Based on previous studies (e.g., Monje et al., Human Schwann Cells in vitro), we recommend caution when working beyond passage 5.

i. In the discussion (line 290) pdSCs were proposed as "tool to study the pathophysiology of individual neuropathies, leading the way to personalized medicine". This statement is exaggerated as distinct PNP associated SC phenotypes / expression profiles / functional differences have not been identified and the repair phenotype, that SCs gain after culture, will also influence such a potential PNP phenotype.

Author response: The reviewer is absolutely right pointing out that the phenotype gained in culture may influence and mask the potential PNP phenotype. Nevertheless, the genetic study of each cell strain and their behavior upon different stimuli, as well as in different culture conditions or passages, can elucidate the PNP-related characteristics of that specific patient. We are at the moment working on analyzing the individual differences among cell strains and their correlations with the neuropathic symptoms seen in each patient. Furthermore, in the near future, we see the potential of adding pdSC to induced human sensory neurons, which are produced in our lab as well, and study their interaction and relationship.

Reviewer response: It is a meaningful goal to compare the individual differences between the pdSC cultures and associations to neuropathic syndromes and the myelination potential towards iPSC-derived sensory neurons. Because your work is so important, the purity of pdSC cultures should be very high to draw relevant conclusions.

Author response: We thank the reviewer for these valuable comments and for emphasizing how important high-purity cultures will be for future patient-specific analyses. While single-cell approaches would indeed provide the most comprehensive assessment of purity, they often bear new questions reflecting broader issues beyond the scope and objectives of the present study. Within these limitations, we have used all feasible methods available to us—including immunostaining for established SC markers, morphological assessment, and comparison with published human SC transcriptomic datasets—to characterize pdSC identity as rigorously as possible. We are grateful for the reviewer's encouragement and will continue refining these approaches as resources allow.

The authors have generated precious RNA-sequencing data of PNP patient derived SC cultures. It would be interesting to compare them to the already existing RNA sequencing data of human SC cultures and try to retrieve information about the differences between dysfunctional PNP-associated SCs and control SCs. Based on that information, functional differences might be identified that could be validated in pdSC cultures. Such findings would be of high interest for the SC community.

Author response: We would like to thank you for the very helpful contribution that your comments have provided to our manuscript. In this article, we have already compared our results with existing RNA sequencing data of human SC (Human Protein Atlas[Ref 34]; Karlsson et al, 2021[Ref 35]; Peng et al, 2020[Ref 36]; Gerber et al; 2021[Ref 37] and Zhang et al, 2021[Ref 38]) in figures 3, 4 and 5. In addition, we also hope that our future studies on individualized PNP patient derived SCs and their PNP-associated phenotypes will indeed be of great interest for the SC community

Reviewer response: The single-cell type transcriptomics map of human tissue does not include nerve tissue. How were the SC_HPA and fibroblast_HPA cell specific markers identified?

Author response: We thank the reviewer for this accurate comment. As noted, the Human Protein Atlas single-cell resource does not contain data from peripheral nerve. Instead, it provides transcriptomic profiles for 154 cell types from 36 databases across multiple human tissues. This can be found in https://www.proteinatlas.org/humanproteome/single+cell/single+cell+type/data#datasets

From this dataset, we extracted the entries corresponding to the "Schwann cell" and "fibroblast" cell types and identified genes that were differentially expressed between these two annotated populations. Because these markers represent cell-type–specific signatures aggregated across multiple tissues and datasets, they offer increased robustness and specificity.

Other:

Line 48: "Fibroblasts do not express the D-amino acid oxidase (DAAO) required to use up D-valine and therefore survive in this particular medium. This results in their elimination and promotes a pure SC culture." If it is true, that fibroblast shall not survive in this medium, why are most pdSCs contaminated with a significant amount of S100-/SOX10- cells as shown in the IF stainings?

Author response: We thank the reviewer for this important question. As mentioned above, we believe that the $S100^{-}/SOX10^{-}$ cells observed in some pdSC cultures are not fibroblast contaminants, but Schwann cells displaying a less mature or fibroblast-like phenotype. This interpretation is supported by the fact that the commercial SCs (cSC) also show low expression of these markers in both our IF stainings and RNAseq, despite having been validated as Schwann cells by the provider and used extensively in the literature.

Regarding the D-valine medium, the function of DAAO in this protocol and in eliminating fibroblasts is further explained in the discussion (lines 268–285) to clarify its mechanism and limitations. In addition, although D-valine medium substantially reduces contaminating fibroblasts, it does not prevent Schwann cells from adopting phenotypic variability, including reduced expression of classical markers. Our results thus support that the cultures are composed of Schwann cells with heterogeneous phenotypes rather than fibroblast contamination.

Line 90-91: "SC typically express specific markers like …, and the myelin binding protein (MBP)." This statement is not true because only in myelinating SCs express MBP. However, upon isolation, myelinating SCs downregulate MBP and other myelin genes because they adapt a repair phenotype.

Author response: We thank the reviewer for this clarification. We agree that MBP is restricted to myelinating Schwann cells and that its expression can be rapidly downregulated after isolation as cells may adopt a repair-like phenotype. Our intention was to list commonly recognized markers associated with different SC phenotypes, not to suggest that MBP is universally expressed in cultured SCs.

Reviewer #2 (Remarks to the Author):
The authors have responded all issues raised. This is an important work for the field.

Response to Reviewers

Manuscript COMMSBIO-24-7498-T

Dear Dr. Bu,

we sincerely thank you and the reviewer for the thoughtful evaluation of our manuscript. We have carefully revised the manuscript to address all points raised. We also apologize for the missing question not addressed in the previous revision.

Regarding the manuscript title, we understand that the isolation of Schwann cells specifically may be one of the possible interpretations of our data, but not the only one. Thus, we have rephrased it to more accurately describe the study presented in this manuscript: "Culture and gene expression profiling of Schwann cells from human sural nerve". We also understand the possibility of a mixed population present in our culture and have therefore discussed extensively the possible interpretations of our data regarding purity.

We appreciate your consideration and believe that the manuscript now more accurately reflects both the strengths and limitations of the study.

Please find our response in blue and the changes in the manuscript in *green (italics)*. In the main text, all changes are highlighted in yellow.

Reviewers' comments:

Reviewer #1 (Remarks to the Author):

Dear authors,

Thank you for addressing most of the points. Again, be ensured that this work is acknowledged and valued. You demonstrated that it is possible to culture Schwann cells from very small nerve biopsies as the immunofluorescence staining showed SOX10 and S100B double positive Schwann cells in the cultures. However, the nerve biopsy cultures also contained SOX10/S100B negative cells, some cultures more, some less. Based on the absence of established Schwann cell markers and the fibroblast-like morphology, the data do not support the interpretation that these cells represent Schwann cells with a fibroblast phenotype. Instead, the findings are more consistent with a contaminating fibroblast population.

The argument that "All cultures were processed following the same protocol and cultured in the same medium, as well as the commercial SC used as control, thus suggesting that the differences we see are based on the origin of the biopsy and not the protocol itself." does not hold because using the same protocol and culture conditions does not exclude differences in cellular composition between samples. Variability in the proportion of Schwann cells versus contaminating cells can arise from differences in the cellular composition or origin of the original biopsy, Schwann cell viability (due to the PNP), donor-dependent variabilities, or selective expansion during culture. This is why a systematic assessment of Schwann cell culture purity is commonly performed when working with cultured Schwann cells.

Concerning the assessment of SC purity the authors write "single-cell analyses would be necessary to determine the exact purity of the SC culture and verify the absence of other cell types". While single-cell RNA sequencing can provide high-resolution insights into cellular heterogeneity, it is not a

prerequisite for determining Schwann cell culture purity. The presence of Schwann cells can be evaluated using standard immunostainings, including the SC marker-based immunostaining against SOX10 and S100B as the authors performed. Those stainings show that the different nerve biopsy cultures contain varying amounts SOX10 and S100B negative cells. SOX10 is a core lineage transcription factor of SCs present in immature, mature and repair SCs; its absence in Schwann cells is unlikely.

Nevertheless, the authors assume that their isolation results in pdSC cultures composed of different Schwann cell phenotypes, including a dominant fibroblast-like phenotype, rather than a much likelier explanation: a contaminating fibroblast population. The statement: "Overall, our data indicate that the cultures contain Schwann cells with donor-dependent phenotypic variability rather than fibroblast contamination." is in fact not demonstrated by the data. The presented data show that the nerve biopsy cultures from different donors contain different amounts of SOX10/S100B positive Schwann cells. This is also nicely reflected in the bulk sequencing data. Then, the expression profiles of pdSC cultures are compared to different Schwann cell and fibroblast associated markers derived from different sources but all showing the same, dependent on the donor, some nerve biopsy cultures have more markers in common with Schwann cells, some have more markers in common with fibroblasts. While these data would usually suggest that there are different amounts of fibroblasts present in the pdSC cultures, the authors conclude that the established nerve biopsy cultures represent Schwann cells with a fibroblast phenotype "supported by the fact that the commercial SCs (cSC) also show low expression of these markers in both our IF stainings and RNAseq". In turn, it is not questioned that commercially available Schwann cell cultures show that strong resemblance with fibroblasts.

It all ends in one question: how would the data look like if the pdSC cultures would contain different amounts of contaminating fibroblasts? The answer is: exactly the same. Thus, the authors cannot discriminate if the isolated cells are Schwann cells with a fibroblast phenotype or a mixture of Schwann cells and fibroblasts.

Authors:

We sincerely thank you for your careful evaluation and for your kind recognition that our work demonstrates the feasibility of culturing Schwann cells from very small human nerve biopsies. We are grateful for the opportunity to clarify and refine our interpretations. We very much discussed your concerns in our team and carefully addressed all points:

We have changed the title to:

"Culture and gene expression profiling of Schwann cells from human sural nerve"

1) We now address the fact that the nerve biopsy–derived cultures contain SOX10/S100B-negative cells and that their relative abundance varies between donors.

Line 238 "Our multiclonal primary cultures also contained varying proportions of SOX10- and S100B-negative cells."

2) We also agree that using the same protocol and culture conditions does not exclude differences in cellular composition between samples, possibly arising from donor variability. We also note that differences may also arise from technical reproducibility limitations or intrinsic cell-to-cell heterogeneity, regardless of standardized protocols [1, 2]. Please find the following change in the text:

line 328: "Furthermore, while all cultures were generated using identical culture conditions, variability between samples may reflect donor-dependent biological factors, as well as arise from technical limitations or intrinsic cell-to-cell heterogeneity".

---

3) We appreciate as well the view on single-cell analyses not being necessary for the completion of this study, while agreeing that it can provide high-resolution insights into cellular heterogeneity.

Our statement was deleted: "Moreover, future work incorporating single-cell transcriptomic analyses will be essential to dissect the cellular composition in greater detail, including distinct SC subtypes and transitional states between clusters."

---

4) Reviewer: *SOX10 is a core lineage transcription factor of SCs present in immature, mature and repair SCs; its absence in Schwann cells is unlikely. …much likelier explanation: a contaminating fibroblast population…*

Indeed, with the present data, we cannot exclude the possibility of fibroblast-derived cells contaminating the multiclonal culture, but we need to consider other options as well. To balance between both points of view, we have carefully reformulated references to purity and homogeneity across the manuscript to avoid subjective or biased language and consider contaminating fibroblasts as a potential limitation. Our both points of view are now carefully addressed and discussed as follows:

Line 239: "Cells lacking these canonical SC markers in SC cultures are frequently interpreted as contaminating fibroblasts [36, 47]. Peripheral nerve biopsies contain several non-SC populations, including endoneurial fibroblasts, which may be co-isolated during tissue dissociation and subsequently expand under in vitro conditions.

However, the presence of SOX10- and S100B-negative cells does not necessarily indicate fibroblast contamination. Schwann cells are known to exhibit considerable phenotypic plasticity, and loss of canonical markers may also arise from in vitro dedifferentiation or rejuvenation processes [48-52]. When glial cells from both the central and peripheral nervous systems regain proliferative capacity in culture, they can acquire stem-cell–like features reminiscent of developmental multipotency [53-55]. Consistent with this notion, Schwann cells have been reported to undergo marked phenotypic changes under in vitro conditions. For instance, proliferative culture environments, including high serum concentrations or mitogenic stimulation such as those used in the present protocol, can promote cell division while simultaneously downregulating canonical Schwann cell markers, including SOX10 [11, 56]. Moreover, experimental loss of SOX10 in SC has been associated with increased proliferation, enhanced focal adhesions, and a flattened morphology similar to that observed in a subset of our cultures [57].

Taken together, these observations make it difficult to determine whether the fibroblast-like phenotypes observed here primarily reflect contaminating fibroblasts co-isolated from the original nerve biopsy or Schwann cell–derived populations that have adopted alternative phenotypic states under the applied culture conditions. It is therefore likely that both mechanisms contribute to the SOX10/S100B-negative cell population detected in our cultures."

5) **Reviewer:** *Then, the expression profiles of pdSC cultures are compared to different Schwann cell and fibroblast associated markers derived from different sources but all showing the same, dependent on the donor, some nerve biopsy cultures have more markers in common with Schwann cells, some have more markers in common with fibroblasts.*

We answer to this point as follows:

Line 112: To molecularly characterize pdSCs, we performed bulk RNA sequencing on patient-derived pdSCs and compared their transcriptomes with those of cSCs at three passages (DIV7). Nerve fascicles (NF) cultured for 48 h post-harvest were included as source tissue controls. Given potential fibroblast contamination and donor-specific variability in SOX10/S100B double-positive cells (Suppl. Fig. 2), a human fibroblast culture (Fibroblast_1) was analyzed as a reference. Sequencing yielded 2–4 × 10^7 reads per sample (Suppl. Fig. 3A). Principal component analysis and sample distance heatmaps demonstrated strong within-group consistency (Suppl. Fig. 3B, C). Despite variability in fibroblast- and Schwann cell–associated signatures across individual cultures, all pdSC samples clustered closely together and were clearly separated from cSC, NF, and Fibroblast_1.

---

6) The reviewer also points out that "*it is not questioned that commercially available Schwann cell cultures show that strong resemblance with fibroblasts.*".

The statement reminds us to add a point of view to the discussion:

Lines 312: "We used commercially available Schwann cells (cSCs) as a reference in our study. Previous reports have shown that these cells display key hallmarks of Schwann cells [87-89]. In our hands, cSCs exhibited proliferation behavior and morphology comparable to those observed in our pdSCs. However, transcriptome analysis revealed that these cell preparations formed a distinct cluster, clearly separated from both fibroblasts and our patient-derived SCs. These findings suggest that morphological assessment, proliferation characteristics, and antibody staining alone may not be sufficient to reliably define cellular identity in vitro. Instead, bulk mRNA sequencing provides a robust molecular approach for the characterization and classification of cell preparations and how they behave in individual laboratories. Given the increasing accessibility of sequencing technologies and open science datasets, transcriptomic profiling represents a valuable tool for verifying cell identity and for comparing cell lines across laboratories with high transparency."

Other:

The authors state that "Fibroblasts (Fibroblast_HPA) and SC (SC_HPA) single-cell transcriptomic consensus from Human Protein Atlas version 23.0 (HPA), obtained from 31 different tissues were compared in order to find in vivo specific markers for each cell type". However, the Human Protein Atlas does not provide a curated or validated consensus Fibroblast or Schwann cell gene signature. It remains unclear which tissue, dataset, cell clusters, and selection criteria were used to define this signature. Please clarify this in the methods part.

Please find a new paragraph in the method describing the dataset, cell clusters, and selection criteria used to define the cell culture signature.

Line 516: "Transcriptomic analyses were conducted using TPM-normalized gene expression values derived from RNA sequencing. Gene expression patterns were visualized with heatmaps in GraphPad Prism and radar charts in Microsoft Excel. Single-cell consensus profiles for fibroblasts (Fibroblast_HPA) and Schwann cells (SC_HPA) were obtained from the Human Protein Atlas. [34, 35]. Eight genes with the highest (fibroblast markers) and lowest (Schwann cell markers) Log2FC between these cell types were selected and compared with our datasets.

To assess similarity to fibroblast or Schwann cell phenotypes, mean TPM values of selected marker genes were visualized in heatmaps. Log2FC values between group averages were used to calculate a similarity score ranging from −10 to 10. Samples with Log2FC ≤ −5 were classified as fibroblast-like, whereas those with Log2FC ≥ 5 were classified as Schwann cell–like (Fig. 3D, Supplementary Table 1).

Representative in vitro marker genes were defined using datasets from primary cultures of adult human peripheral nerve cells. [36]. Identified Schwann cell– and fibroblast-related markers were mapped to our TPM data and visualized in heatmaps. Additional published datasets on peripheral nerve–resident cell types informed classification of human-derived Schwann cell cultures. [37, 38]. For each subtype, 20 genes were selected based on reported Log2FC values, and their TPM expression in pdSC samples was visualized using radar charts and heatmaps (Fig. 5, Supplementary Tables 3–4)."

The Thy.1 staining, while appreciated, looks very strange because Thy.1 should be expressed at the cell membrane but shows a nuclear staining. How do the authors explain this?

We checked for reference data and agree that we cannot trust the label.

One reviewer comment about the n number was not addressed: There seems to have been a misunderstanding. The presented n number of 6 is not a limitation of this study. However, defining triplicates from one donor as n = 3 is methodologically incorrect, as these do not represent independent biological replicates. This should be clarified in the methods section and in the respective figure legend to avoid misinterpretation of sample numbers or statistics.

We are sorry. We have overlooked this reviewer comment. Please note our clarification here:

lines 451: *"The cSCs were commercial and derived from a single donor (n = 1 biological replicate). The pdSC cultures derived from 6 independent donors (n = 6 biological replicates). Technical replicates and pdSCs at different passages were included in the final analyses as part of the total n-number."*

References

1. Reddin, I.G., et al., *Large inherent variability in data derived from highly standardised cell culture experiments.* Pharmacol Res, 2023. **188**: p. 106671.
2. Kusena, J.W.T., et al., *The importance of cell culture parameter standardization: an assessment of the robustness of the 2102Ep reference cell line.* Bioengineered, 2021. **12**(1): p. 341-357.

Response to Reviewers

Manuscript COMMSBIO-24-7498-T

Dear Dr. Bu,

we sincerely thank you and the reviewer for the thoughtful evaluation of our manuscript. We have carefully revised the manuscript to address all points raised. We appreciate your consideration and believe that this version of the manuscript reflects both the strengths and limitations of the study. Please find our response in blue and the changes in the manuscript in *green (italics)*. In the main text, we marked our responses in yellow.

To the Editor: Regarding the reviewer's main concern:

In our manuscript, we explicitly report the limitation that we cannot exclude that fibroblasts contribute to the transcriptome data and are present in the cultures.

Line 256 – 259: "Given these considerations, we acknowledge the limitation that fibroblasts co-isolated from the original biopsy may be present in the cultures. In addition, fibroblast-like phenotypes may reflect Schwann cells or other co-isolated cell types adopting alternative states under the applied culture conditions."

Comments from Reviewer #1:

Dear authors,

the revised wording helps to introduce the hypothesis that a varying population of $SOX10^-/S100B^-$ Schwann cells may exist in neuropathic sural nerves, or that Schwann cells isolated from such tissue may adopt a SOX10-/S100B- fibroblast-like phenotype in culture, concepts that are not yet established.

However, the manuscript would need to be revised accordingly, as several sections still imply the generation of a pure, bona fide Schwann cell culture using this method.

Several methodological aspects still need to be clarified, including the correct use of "n"-numbers, how the different "Schwann cell subtypes" were defined and extracted from the published scRNA-seq datasets, and how the curated or validated consensus fibroblast and Schwann cell gene signatures were derived from the Human Protein Atlas (see points below).

If the authors conclude that the initial isolation yields a pure Schwann cell population that subsequently undergoes pronounced reprogramming toward a fibroblast-like phenotype in culture, the culture conditions should be optimized to prevent or minimize this effect. Otherwise, it remains questionable whether these cultures can serve as a reliable tool for studying neuropathy pathophysiology and for advancing personalized medicine approaches.

Finally, the question of how the data would appear if the pdSC cultures contained varying proportions of contaminating fibroblasts remains a central point of concern. The current data do not allow discrimination between Schwann cells adopting a fibroblast-like phenotype and a mixed population of Schwann cells and fibroblasts. Therefore, careful wording and balanced argumentation are essential when introducing this hypothesis.

Response:

"…several sections still imply the generation of a pure, bona fide Schwann cell culture…"

Authors: We thank the reviewer for highlighting this point. In our manuscript, we explicitly report the limitation that we cannot exclude that fibroblasts contribute to the transcriptome data and are present in the cultures.

Line 243 – 259: "Our multiclonal primary cultures also contained varying proportions of SOX10- and S100B negative cells. Cells lacking these markers in SC cultures are frequently interpreted as contaminating fibroblasts [37, 48]. Peripheral nerve biopsies contain several non-SC populations, including endoneurial fibroblasts, which may be co-isolated during tissue dissociation and subsequently expand under in vitro conditions. On the other hand, SC are also known to display considerable phenotypic plasticity in culture. In particular, proliferative culture conditions, including high serum concentrations or mitogenic stimulation such as those used in this protocol, have been reported to promote proliferation and to downregulate canonical SC markers, including SOX10 [11, 49]. Moreover, experimental loss of SOX10 in SC has been associated with increased proliferation, enhanced focal adhesions, and a flattened morphology similar to that observed in some of our cultures [50]. Lastly, during early protocol optimizations, fibroblasts were clearly present in our cultures and they rapidly overgrew under high-serum conditions, hindering SC isolation, which is no longer observed.

Given these considerations, we acknowledge the limitation that fibroblasts co-isolated from the original biopsy may be present in the cultures. In addition, fibroblast-like phenotypes may reflect Schwann cells or other co-isolated cell types adopting alternative states under the applied culture conditions."

We have revised the manuscript throughout to avoid wording implying homogeneous Schwann cell cultures. The wording was adjusted to consistently reflect heterogeneous cultures.

lines 50 and 59: "pure" was removed

lines 21–23 (Abstract)

lines: 57–61 (Introduction)

lines 225–230 (Discussion)

lines 309–311 (Discussion)

---

1. To strengthen the title while maintaining transparency regarding the heterogeneous cell populations, the following title is suggested: "Transcriptomic profiling reveals heterogeneity in primary cultures derived from human polyneuropathic sural nerve biopsies". This title would provide room to address

the authors' hypothesis of a not yet established view that Schwann cells may adopt a fibroblast-like phenotype in culture.

Authors: We thank the reviewer for this thoughtful suggestion and agree that the proposed title appropriately reflects the heterogeneous nature of the cell populations observed in our cultures. However, we feel that the suggested title does not fully capture the primary focus and main finding of our study, namely the isolation and transcriptomic characterization of Schwann cells derived from very small human sural nerve biopsies. While cellular heterogeneity is an important aspect of our results and is discussed in the manuscript, we believe that our current title more accurately reflects the overall scope and central message of the work. Therefore, we respectfully prefer to retain the original title.

2: Even though the authors the authors know of all the limitations, they write that "In this study, we present a novel protocol to culture human SC from ~4-mm diagnostic sural nerve biopsies. Cells isolated with this method were characterized and expanded, in order to create well-defined primary Schwann cell cultures derived from patients with PNP that could serve as tools for diagnostic and therapeutic purposes. The application of this protocol resulted in the establishment of a culture of spindle-shaped cells that tested positive for the transcription factor SOX10 in their nuclei, and for the proteins S100B and MBP in their cytoplasm, resembling previously described SC hallmarks [39-43]. The analysis of their transcriptome confirmed the expression of S100B and SOX10, but not MBP, in cells cultured with this method." or "In this study, we describe a protocol that incorporates key elements of previously published culture conditions while enabling the successful use of D-valine medium to isolate and expand a pure Schwann cell culture from ~4 mm diagnostic human sural nerve biopsies."

These paragraphs are misleading as it suggests the culture of pure, SOX10+/S100B+ Schwann cell cultures.

Authors: We thank the reviewer for this important comment. We agree that the original wording could be misinterpreted as implying the generation of purely Schwann cell–enriched cultures. The corresponding changes are already listed above:

(lines 50 and 59: "pure" was removed; lines 21–23 (Abstract); lines: 57–61 (Introduction); lines 220–225 (Discussion); lines 305–307 (Discussion)

---

3. The description of sample size is still misleading. While the authors state that Schwann cell cultures were derived from 6 independent donors (n = 6 biological replicates), they also indicate that technical replicates and cells from different passages were included in the total n-number. Technical replicates and repeated measurements from the same donor (e.g. different passages) do not constitute independent biological replicates and should not be included in the reported n. This approach artificially inflates sample size and may lead to incorrect statistical inference. The authors should clearly distinguish between biological replicates, technical replicates, and repeated measurements in every figure and revise any statistical analysis accordingly.

Authors: The distinction between biological and technical replicates can be found in the Methods section.

Lines 454-457: *"The cSCs were commercial and derived from a single donor (n = 1 biological replicate). The pdSC cultures were derived from 6 independent donors (n = 6 biological replicates). Technical replicates and pdSCs at different passages were included in the final analyses as part of the total n-number."*

---

4. The Human Protein Atlas does not provide a curated or validated consensus Fibroblast or Schwann cell gene signature. It remains unclear which tissue, dataset, cell clusters, and selection criteria were used to define this signature. The revised methods part does not include that information.

Authors: We thank the reviewer for this comment. A curated or experimentally validated consensus gene signature for fibroblasts or Schwann cells in adult human sural nerve is not available. In addition, obtaining sural nerve biopsies from healthy individuals is not ethically feasible, which precludes the establishment of a true healthy reference dataset for this tissue.

We therefore used the Human Protein Atlas marker gene sets as a widely used reference framework, for cell-type–associated expression rather than as a tissue-specific or ground-truth signature. We now cite the original publication (Uhlén et al., Science, 2015), which has been cited for more than 18,000 times until now. These datasets represent one of the few systematically curated resources applicable to human tissues in the absence of a validated sural nerve–specific benchmark.

We would like to pronounce that we explicitly clarify the origin of the datasets, the tissue sources on which they are based, and the criteria by which marker genes were selected for downstream analyses.

(Lines 522–540): *"Human Protein Atlas (HPA) single-cell transcriptomic data (version 23.0) were used as a reference dataset for in vivo gene expression profiles [34-36]. The HPA single-cell type atlas integrates and re-processes publicly available single-cell and single-nucleus RNA sequencing (scRNA-seq/snRNA-seq) datasets from healthy adult human tissues using a unified processing pipeline. Version 23.0 comprises expression profiles from 81 annotated cell types across 31 human tissues. The underlying datasets originate from publicly available repositories, including the Human Cell Atlas, Gene Expression Omnibus (GEO), Single Cell Expression Atlas, EMBL-EBI BioStudies, and Tabula Sapiens.*

Normalized gene expression values (nCPM) were obtained from the Human Protein Atlas single-cell type data portal and the HPA file "rna_single_cell_type.tsv.zip" (available at: (https://www.proteinatlas.org/humanproteome/single+cell/single+cell+type/data#cell_type_data). This file contains normalized transcript expression values for all annotated cell types, including Schwann cells and fibroblasts. Schwann cell and fibroblast expression profiles were extracted based on HPA cell-type annotations. Putative cell type–enriched marker genes were identified by calculating the Log2 fold change (Log2FC) between these two cell types, and selecting the eight genes with the highest Log2FC (fibroblast-enriched markers) and the eight genes with the lowest Log2FC (Schwann cell-enriched markers). These gene sets were subsequently applied to TPM-normalized pdSC transcriptomic data for comparative analyses. All selected genes are provided in Supplementary Table 1."

In addition, we have already included a detailed list of all marker genes used, together with their source annotation, in the Supplementary Materials (Supplementary Table 1). These additions are intended to improve transparency and reproducibility of our marker-based comparisons.

In addition, the methods part does not contain how the genes for the different "SC subtypes" have been selected. The authors wrote "To identify the distinct phenotype of pdSC, markers from previous studies performing single-cell RNA sequencing of peripheral nerve specimens were analyzed. From two reference data sets [37, 38], twenty genes corresponding to each SC subtype were selected and their TPMs in pdSC were represented in radar charts (Fig. 5A, B, Suppl. Table 3)." In Zhang et al, they defined SC subpopulations of day 1 of age neonatal Sprague–Dawley rats. In Gerber et al, the extracted nerve cells from newborn mice at P1, and cells at a young adult stage at P60. The authors did not specify from which datasets the "SC subtypes" were extracted. In addition, those publications use newborn or young naïve nerves as source. With regard to the patient derived data, it would be more appropriate to use scRNA-seq data from older rodents and might also involve an injury context, e.g: Lovatt D, Tamburino A, Krasowska-Zoladek A, Sanoja R, Li L, Peterson V, Wang X, Uslaner J. scRNA-seq generates a molecular map of emerging cell subtypes after sciatic nerve injury in rats. Commun Biol. 2022 Oct 19;5(1):1105. doi: 10.1038/s42003-022-03970-0. PMID: 36261573; PMCID: PMC9581950.

Authors: We thank the reviewer for this helpful suggestion. In response, we have now clarified in the Methods section (lines 538–557) the origin and biological context of the reference datasets used in this study, as well as the selection criteria for Schwann cell subtype markers. These markers are also explicitly listed in the Supplementary Materials (Supplementary Tables 3-4), to improve transparency and reproducibility.

Line 548 – 566 (Methods): *"Published scRNA-seq datasets of peripheral nerve–resident cell types were used as reference frameworks for Schwann cell subtype classification. From Zhang et al. [38], marker genes for cell types identified in neonatal (P1) rat sciatic nerves and DRG were obtained from Supplementary Table 1 ("glia23928-sup-0001-TableS1.xlsx"), excluding the erythrocyte cluster. Marker genes for four Schwann cell subtypes were obtained from Supplementary Table 2 ("glia23928-sup-0002-TableS2.xlsx"). For each annotated cell type, the top twenty marker genes were selected by ranking genes within each cluster according to their reported average log2 fold-change (avg_logFC) values. From Gerber et al. [39], we extracted differentially expressed marker genes from Supplementary file 3 ("elife-58591-supp3.xls"). Schwann cell markers were obtained from the "SmartSeq2 related to Figure 4" sheet, which contains clusters from mouse sciatic nerves spanning postnatal day 1 (P1) to adulthood (P60). Markers for additional cell types at P1 were obtained from the "10x_P1 related to Figure 6" sheet. Fibroblast markers were derived from the fibroblast-related cluster (FbRel*); the Schwann cell clusters and the proliferating fibroblast-like (prol. Fb) cluster were not included. Otherwise, cell types were defined according to the reported cluster annotations. For each cluster, the top twenty differentially expressed marker genes were selected by ranking genes within each cluster according to their reported average natural-log fold-change (avg_logFC) values.*

These gene sets were used for comparative analysis of pdSC transcriptomes and do not represent direct biological equivalents to the human samples analyzed here."

With regard to the additional dataset suggested by the reviewer, we agree that it may provide valuable complementary information. However, we were unable to access the original data, as the provided GEO link appears to be invalid and the corresponding contact email was no longer functional. Despite our attempts to obtain the dataset by contacting the authors, we were not successful. Therefore, we were unfortunately unable to include this dataset in our analyses.
