# Supplementary material for: Culture and gene expression profiling of Schwann cells from human sural nerve
Source: Commun Biol. 2026 Sep 28;9:1273. doi: 10.1038/s42003-026-11023-z (PMC13627658; doi:10.1038/s42003-026-11023-z)
Supplement: Supplementary file 2 [file 42003_2026_11023_MOESM2_ESM.pdf]

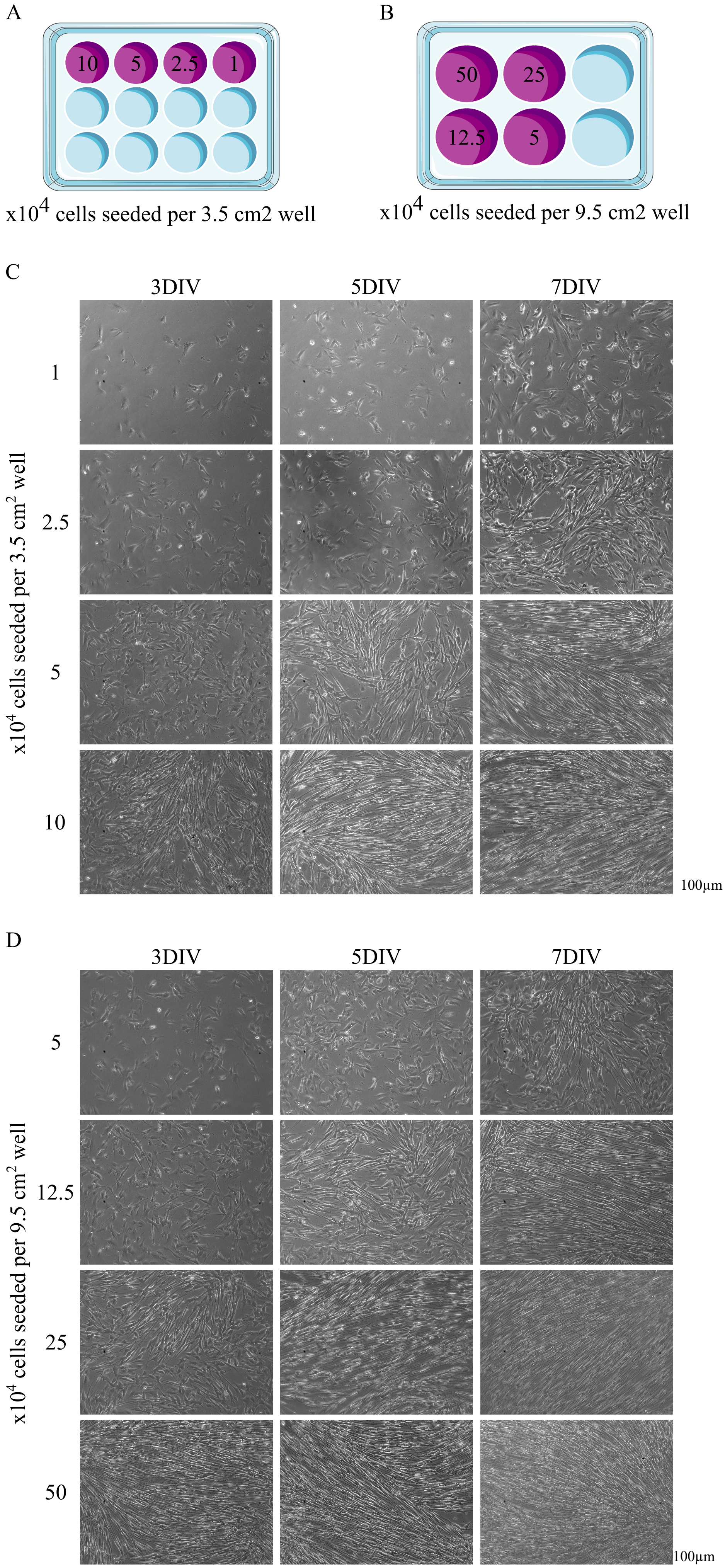

Supplementary Figure 1. Patient-derived Schwann cells (pdSC) morphology and confluence. Graphical representation of the seeding densities of pdSC per well in a 12-well (**A**) and 6-well (**B**) plate. Phase con trast images of pdSC cultured in a 12-well (**C**) and 6-well (**D**) plate, upon different seeding densities, after 3, 5 or 7 days in vitro (DIV).

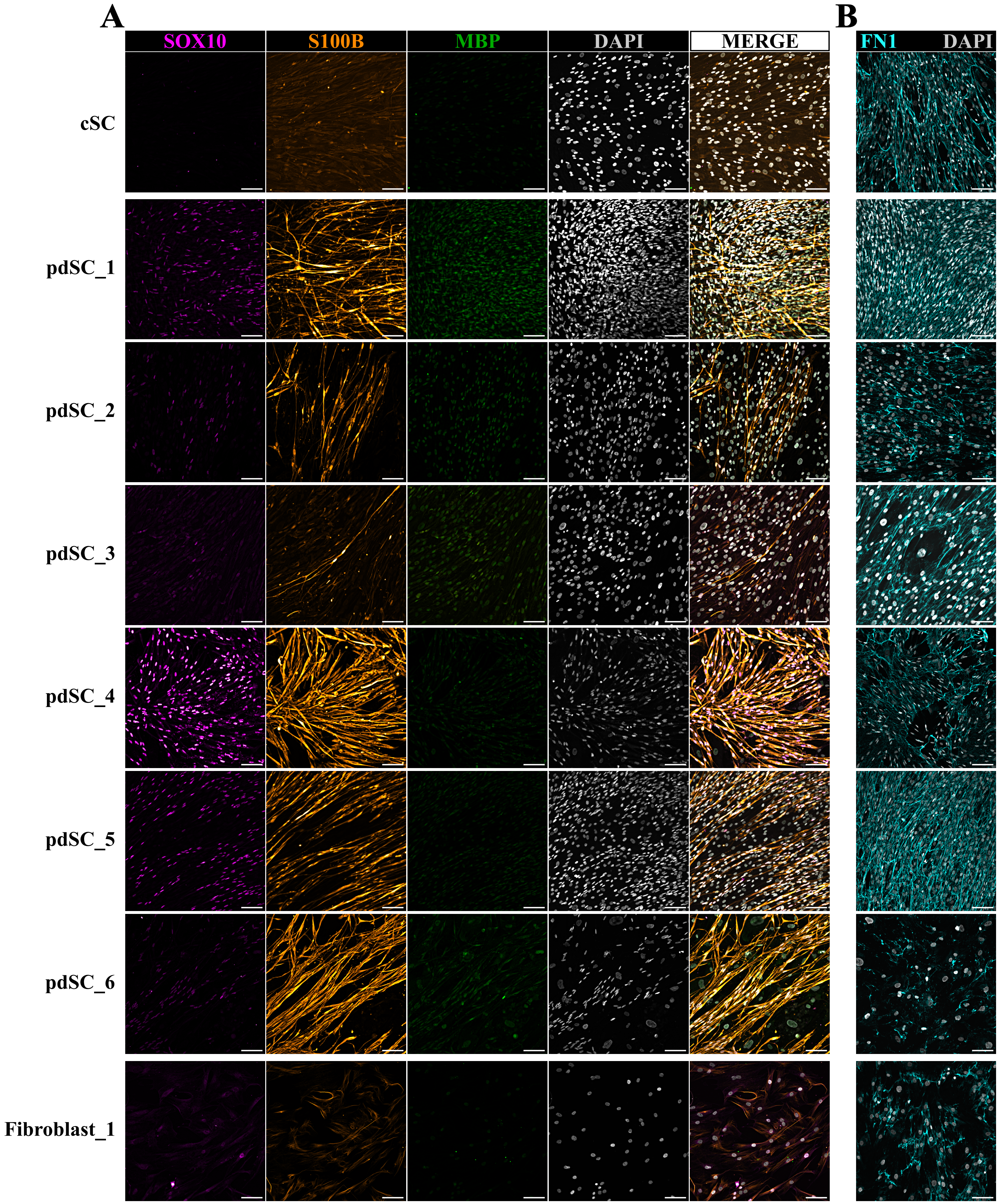

Supplementary Figure 2. Schwann cell marker characterization of patient-derived SC. A) Immunocytochemistry staining of SOX10 (magenta), S100B (orange) and MBP (green) in control human SC (**cSC**), patient-derived SC (**pdSC**) and fibroblast (**Fibroblast_1**) cell cultures at DIV7. **B)** Immunocytochemistry staining of FN1 (cyan) in control human SC (**cSC**), patient-derived SC (**pdSC**) and fibroblast (**Fibroblast_1**) cell cultures at DIV7. Nuclei were stained with DAPI (grey). Scale bar: 100µm.

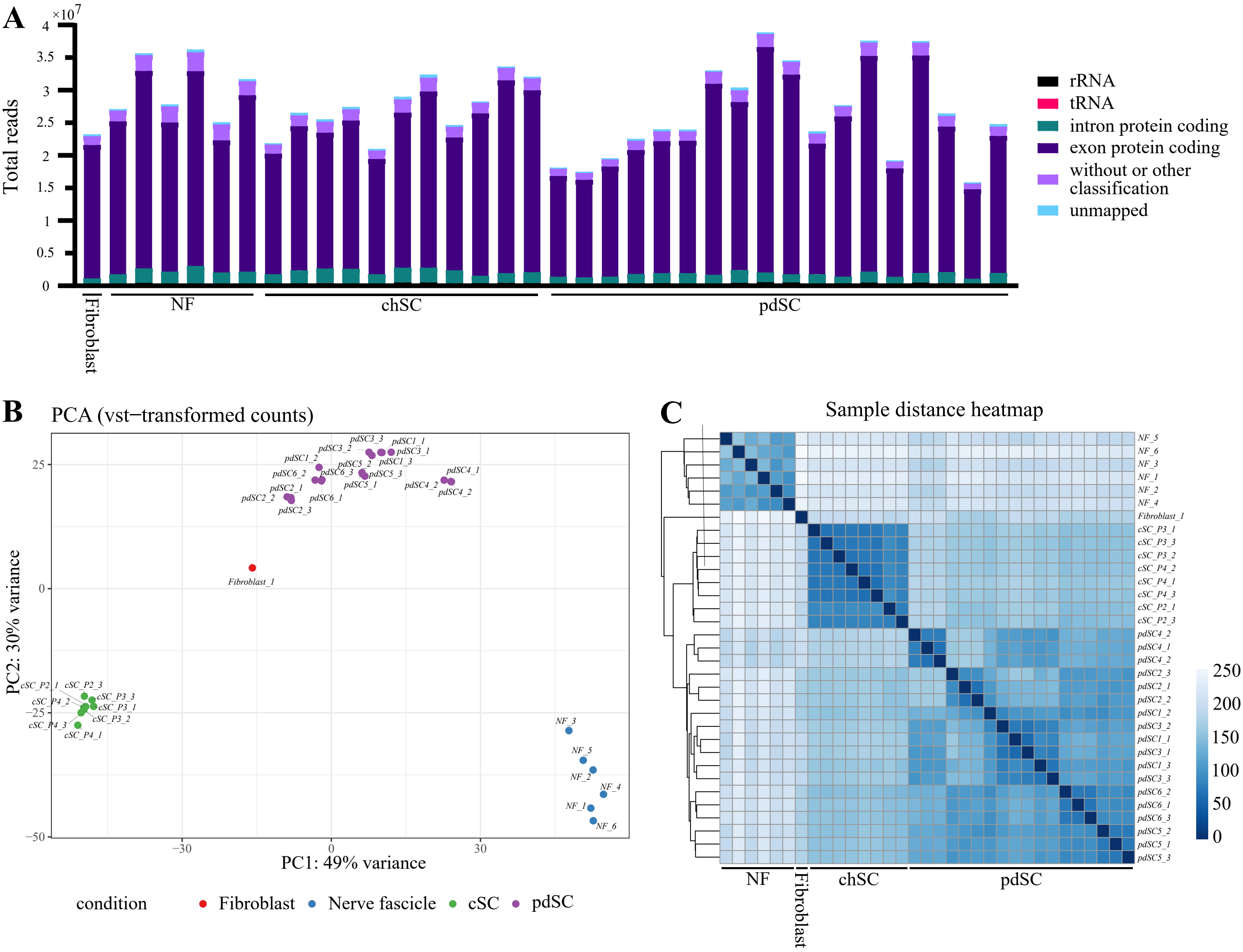

Supplementary Figure 3. Bulk RNA-seq analysis. A) Number of reads per sample and their distribution as rRNA, tRNA, intron protein coding, exon protein coding, without or other classification and unmapped. Principal component analysis (**B**) and sample distance heatmap (**C**) separating each group of samples included in the analysis into distinct clusters. NF, nerve fascicle; cSC, control human Schwann cells; pdSC, patient-derived Schwann cells.
