# Supplementary material for: Culture and gene expression profiling of Schwann cells from human sural nerve
Source: Commun Biol. 2026 Sep 28;9:1273. doi: 10.1038/s42003-026-11023-z (PMC13627658; doi:10.1038/s42003-026-11023-z)
Supplement: Supplementary file 3 [file 42003_2026_11023_MOESM3_ESM.pdf]

Description of Additional Supplementary Files:

File: Supplementary Data

Description: Raw data sets of Figures 2-5.
