# Supplementary material for: Culture and gene expression profiling of Schwann cells from human sural nerve
Source: Commun Biol. 2026 Sep 28;9:1273. doi: 10.1038/s42003-026-11023-z (PMC13627658; doi:10.1038/s42003-026-11023-z)
Supplement: Supplementary file 4 [file 42003_2026_11023_MOESM5_ESM.pdf]

nature portfolio

Corresponding author(s): Patricia García-Fernández

Last updated by author(s): Dec 18, 2025

Reporting Summary

Nature Portfolio wishes to improve the reproducibility of the work that we publish. This form provides structure for consistency and transparency in reporting. For further information on Nature Portfolio policies, see our Editorial Policies and the Editorial Policy Checklist.

Statistics

For all statistical analyses, confirm that the following items are present in the figure legend, table legend, main text, or Methods section.

n/a	Confirmed	
☐	☒	The exact sample size (n) for each experimental group/condition, given as a discrete number and unit of measurement
☐	☒	A statement on whether measurements were taken from distinct samples or whether the same sample was measured repeatedly
☐	☒	The statistical test(s) used AND whether they are one- or two-sided<br>*Only common tests should be described solely by name; describe more complex techniques in the Methods section.*
☐	☒	A description of all covariates tested
☐	☒	A description of any assumptions or corrections, such as tests of normality and adjustment for multiple comparisons
☐	☒	A full description of the statistical parameters including central tendency (e.g. means) or other basic estimates (e.g. regression coefficient) AND variation (e.g. standard deviation) or associated estimates of uncertainty (e.g. confidence intervals)
☒	☐	For null hypothesis testing, the test statistic (e.g. F, t, r) with confidence intervals, effect sizes, degrees of freedom and P value noted<br>*Give P values as exact values whenever suitable.*
☒	☐	For Bayesian analysis, information on the choice of priors and Markov chain Monte Carlo settings
☒	☐	For hierarchical and complex designs, identification of the appropriate level for tests and full reporting of outcomes
☒	☐	Estimates of effect sizes (e.g. Cohen's d, Pearson's r), indicating how they were calculated

Our web collection on statistics for biologists contains articles on many of the points above.

Software and code

Policy information about availability of computer code

Data collection	*Provide a description of all commercial, open source and custom code used to collect the data in this study, specifying the version used OR state that no software was used.*
Data analysis	DESeq2 v1.24.0 was used to conduct principle component analysis by applying the DESeq function with “betaPrior=TRUE” to normalize the data followed by variance stabilizing transformation by applying the vst function. The transformed data was plotted using the plotPCA function together with methods from R package ggplot2 v3.3.2 (Suppl. Fig. 3B). In addition, sample to sample distances were calculated using the R dist function on vst-transformed counts and the resulting distance matrix was plotted using the R package pheatmap v1.0.12. Statistical analysis was performed in SPSS 27 (IBM, Armonk, NY, USA) and Data results were plotted in GraphPad Prism 9 (GraphPad Software, Inc., La Jolla, CA, USA) for visualization.

For manuscripts utilizing custom algorithms or software that are central to the research but not yet described in published literature, software must be made available to editors and reviewers. We strongly encourage code deposition in a community repository (e.g. GitHub). See the Nature Portfolio guidelines for submitting code & software for further information.

Data

Policy information about availability of data

All manuscripts must include a data availability statement. This statement should provide the following information, where applicable:

- Accession codes, unique identifiers, or web links for publicly available datasets
- A description of any restrictions on data availability
- For clinical datasets or third party data, please ensure that the statement adheres to our policy

A step-by-step technical description of the method presented in this article is available at protocols.io, DOI: dx.doi.org/10.17504/protocols.io.36wgqnxj3gk5/v1 (Private link for reviewers: https://www.protocols.io/private/10F45001A2A511F0B9B00A58A9FEAC02 to be removed before publication.). Raw human bulk RNA-seq data generated in this study have been deposited in the European Genome-phenome Archive (EGA) under accession number EGAS50000000737. All the numeric data used in Figures 2, 3, 4 and 5 are included as supplementary tables provided with this article and as an additional excel file.

Research involving human participants, their data, or biological material

Policy information about studies with human participants or human data. See also policy information about sex, gender (identity/presentation), and sexual orientation and race, ethnicity and racism.

Reporting on sex and gender	Sex and gender were not considered in this manuscript.
Reporting on race, ethnicity, or other socially relevant groupings	We did not consider race, ethnicity or any other groupings.
Population characteristics	Six patients with painful PNP who were seen for diagnostic work-up and who underwent a diagnostic sural nerve biopsy were included. Diagnosis was made based on medical history, neurological examination, laboratory, and nerve conduction studies (Table 1). The median age of the patients (2F/4M) was 68.5 years (range 57–87), and median disease duration was 3.5 years (range 0.017–15 years).
Recruitment	In 2021, six patients with PNP were recruited at the Department of Neurology, University Hospital Würzburg, Germany, where they were seen for diagnostic work-up. Diagnoses were based on history taking and neurological examination, laboratory studies, and nerve conduction examinations.
Ethics oversight	Our study was approved by the Würzburg Medical Faculty Ethics Committee (# 15/19) and all participants gave written informed consent prior to inclusion.

Note that full information on the approval of the study protocol must also be provided in the manuscript.

Field-specific reporting

Please select the one below that is the best fit for your research. If you are not sure, read the appropriate sections before making your selection.

☒ Life sciences ☐ Behavioural & social sciences ☐ Ecological, evolutionary & environmental sciences

For a reference copy of the document with all sections, see nature.com/documents/nr-reporting-summary-flat.pdf

Life sciences study design

All studies must disclose on these points even when the disclosure is negative.

Sample size	Sample size was determined upon availability. 3 technical replicates were used for each biological replicate. For cSC, 3 passages were used as biological replicates, leading to a total sample size of 9, from which one data point was excluded. For pdSC, cell strains were collected from 6 different patients, leading to a total sample size of 18. From each patient, there was one nerve fascicle (NF) sample, thus leading to a sample size of 6. Finally, from fibroblast there was only one sample available.
Data exclusions	Only one data point was excluded from the study due to error in the analysis.
Replication	3 technical replicates were used for each biological replicate. For cSC, 3 passages were used as biological replicates, leading to a total sample size of 9, from which one data point was excluded. For pdSC, cell strains were collected from 6 different patients, leading to a total sample size of 18. From each patient, there was one nerve fascicle (NF) sample, thus leading to a sample size of 6. Finally, from fibroblast there was only one sample available.
Randomization	Not relevant.
Blinding	Not relevant.

Reporting for specific materials, systems and methods

We require information from authors about some types of materials, experimental systems and methods used in many studies. Here, indicate whether each material, system or method listed is relevant to your study. If you are not sure if a list item applies to your research, read the appropriate section before selecting a response.

Materials & experimental systems

n/a	Involved in the study
☐	☒ Antibodies
☒	☐ Eukaryotic cell lines
☒	☐ Palaeontology and archaeology
☒	☐ Animals and other organisms
☒	☐ Clinical data
☒	☐ Dual use research of concern
☒	☐ Plants

Methods

n/a	Involved in the study
☒	☐ ChIP-seq
☒	☐ Flow cytometry
☒	☐ MRI-based neuroimaging

Antibodies

Antibodies used

monoclonal mouse to SOX10 (1:50, #SC-365692, Santa Cruz, RRID: AB_10844002), monoclonal rabbit to S100B (1:500, #ab52642, Abcam, RRID:AB_882426), monoclonal rat to MBP (1:100, #ab7349, Abcam, RRID:AB_305869) and monoclonal mouse to FN1 (1:100, #SC-8422, Santa Cruz, RRID: AB_627598)

Validation

monoclonal mouse to SOX10 (1:50, #SC-365692, Santa Cruz, RRID: AB_10844002): https://www.scbt.com/p/sox-10-antibody-a-2?srsltid=AfmBOoq8qI1je6ZwKjNm-t3C8Sdta0oB6fupJYRvVOPIqhf_AFnnwFPp
Validated for the detection of sox10 in mouse, rat and human, in the following applications: WB, IP, IF, IHC(P), ELISA.
Cited in 96 publications.

monoclonal rabbit to S100B (1:500, #ab52642, Abcam, RRID:AB_882426):https://www.abcam.com/en-us/products/primary-antibodies/s100-beta-antibody-ep1576y-astrocyte-marker-ab52642?srsltid=AfmBOop8J3mydBRZcRDI1ZDDxvjUtrxMAQw7VyHKgLOsxwFe3o-qOx-r
Validated for the detection of S100B in mouse, rat and human, in the following applications: Western Blot, IHC-P, IHC-Fr, ICC/IF.
Cited in over 390 publications.

monoclonal rat to MBP (1:100, #ab7349, Abcam, RRID:AB_305869):https://www.abcam.com/en-us/products/primary-antibodies/myelin-basic-protein-antibody-12-oligodendrocyte-marker-ab7349?srsltid=AfmBOopR5GuFHB2H1E47cZaghns8Nfz17TaFkmB-p2oMY7AotZRwKobn
Validated for the detection of MBP in mouse, rat and human, in the following applications: Western Blot, IHC-P, ICC/IF.
Cited in over 310 publications.

monoclonal mouse to FN1 (1:100, #SC-8422, Santa Cruz, RRID: AB_627598):https://www.scbt.com/p/fibronectin-antibody-ep5?srsltid=AfmBOopJxpB3IeMHQuTMlMsFcCVZMZLOWGC2nYQpKK6MJPkxqDRXKfyd
Validated for the detection of FN1in mouse, rat and human, in the following applications: WB, IP, IF, IHC(P) and ELISA.
Cited in 325 publications.

Plants

Seed stocks

Report on the source of all seed stocks or other plant material used. If applicable, state the seed stock centre and catalogue number. If plant specimens were collected from the field, describe the collection location, date and sampling procedures.

Novel plant genotypes

Describe the methods by which all novel plant genotypes were produced. This includes those generated by transgenic approaches, gene editing, chemical/radiation-based mutagenesis and hybridization. For transgenic lines, describe the transformation method, the number of independent lines analyzed and the generation upon which experiments were performed. For gene-edited lines, describe the editor used, the endogenous sequence targeted for editing, the targeting guide RNA sequence (if applicable) and how the editor was applied.

Authentication

Describe any authentication procedures for each seed stock used or novel genotype generated. Describe any experiments used to assess the effect of a mutation and, where applicable, how potential secondary effects (e.g. second site T-DNA insertions, mosiacism, off-target gene editing) were examined.
